# Sequential Targeting Chondroitin Sulfate‐Bilirubin Nanomedicine Attenuates Osteoarthritis via Reprogramming Lipid Metabolism in M1 Macrophages

**DOI:** 10.1002/advs.202411911

**Published:** 2025-01-10

**Authors:** Caifeng Deng, Yongbing Xiao, Xuan Zhao, Hui Li, Yuxiao Chen, Kelong Ai, Ting Jiang, Jie Wei, Xiaoyuan Chen, Guanghua Lei, Chao Zeng

**Affiliations:** ^1^ Department of Orthopaedics, Xiangya Hospital Central South University Changsha Hunan 410008 China; ^2^ Key Laboratory of Aging‐related Bone and Joint Diseases Prevention and Treatment Ministry of Education, Xiangya Hospital Central South University Changsha Hunan 410008 China; ^3^ Hunan Key Laboratory of Joint Degeneration and Injury, Xiangya Hospital Central South University Changsha Hunan 410008 China; ^4^ Xiangya School of Pharmaceutical Sciences Central South University Changsha Hunan 410008 China; ^5^ Departments of Diagnostic Radiology, Surgery, Chemical and Biomolecular Engineering and Biomedical Engineering, Yong Loo Lin School of Medicine and College of Design and Engineering National University of Singapore Singapore 119074 Singapore; ^6^ Clinical Imaging Research Centre, Centre for Translational Medicine, Yong Loo Lin School of Medicine National University of Singapore Singapore 117599 Singapore; ^7^ Nanomedicine Translational Research Program, Yong Loo Lin School of Medicine National University of Singapore Singapore 117597 Singapore; ^8^ National Clinical Research Center for Geriatric Disorders, Xiangya Hospital Central South University Changsha Hunan 410008 China

**Keywords:** M1 macrophages repolarization, nanoparticles, osteoarthritis, reprogramming of lipid metabolism, targeted therapy

## Abstract

The infiltration and excessive polarization of M1 macrophages contribute to the induction and persistence of low‐grade inflammation in joint‐related degenerative diseases such as osteoarthritis (OA). The lipid metabolism dysregulation promotes M1 macrophage polarization by coordinating the compensatory pathways of the inflammatory and oxidative stress responses. Here, a self‐assembling, licofelone‐loaded nanoparticle (termed LCF‐CSBN), comprising chondroitin sulfate and bilirubin joined by an ethylenediamine linker, is developed to selectively reprogram lipid metabolism in macrophage activation. LCF‐CSBN is internalized by M1 macrophages via CD44‐mediated endocytosis and targets the Golgi apparatus accompanied with the reactive oxygen species‐responsive release of licofelone (LCF, dual inhibitor of arachidonic acid metabolism). LCF‐CSBN effectively promotes M1 to M2 macrophage transition by reprogramming the Golgi apparatus‐related sphingolipid metabolism and arachidonic acid metabolism. Intra‐articularly injected LCF‐CSBN retains in the joint for up to 28 days and accumulates into M1 macrophages. Moreover, LCF‐CSBN can effectively attenuate joint inflammation, oxidative stress, and cartilage degeneration in OA model rats. These findings indicate the promising potential of lipid‐metabolism‐reprogramming LCF‐CSBN in the targeted therapy of OA.

## Graphical abstract

M1 macrophages are excessively infiltrated and activated in the development of osteoarthritis. Here, a self‐assembled nanocarrier (designated as CSBN) based on chondroitin sulfate (CS)‐bilirubin (BR) conjugate is developed to reprogram lipid metabolism in macrophage activation by targeting the Golgi apparatus. LCF‐CSBN indicates long‐term therapeutic efficacy in effectively delaying osteoarthritis progression.

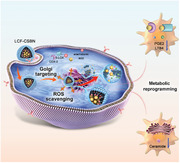

## 1 Introduction

Macrophages are essential components of the innate immune system and display functional plasticity as they respond to different stimuli in physiological and pathophysiological settings.^[^ ^1^, ^2^ ^]^ Macrophages fulfil various homeostatic functions, including maintenance of tissue homeostasis, resolution of inflammation, inflammatory mediation during host defense, tissue remodeling, and metabolic regulation. Two distinct macrophage polarization states exist: the M1 (classically activated) and M2 (alternatively activated) macrophages. Chronic low‐grade inflammation, characterized by the infiltration and activation of M1 macrophages, plays a vital role in the pathogenesis of joint‐related degenerative diseases such as osteoarthritis (OA).^[^ ^3^, ^4^ ^]^ Low‐grade inflammation is primarily characterized by the increase in the levels of several inflammatory mediators.^[^ ^5^ ^]^ The excessive polarization of M1 macrophages helps establish an inflammatory microenvironment and contributes to the maintenance of inflammation.^[^ ^6^ ^]^ Thus, selectively enhancing the M1 to M2 phenotypic transition of macrophages may inhibit low‐grade inflammation and suppress the progression of OA.

Cellular metabolism has emerged as a critical regulator of macrophage activation, function, and phenotype. Indeed, distinct metabolic programs are necessary to control macrophage polarization, leading to the transcriptional activation of pro‐inflammatory or anti‐inflammatory genes.^[^ ^1^ ^]^ Changes in macrophage metabolism have emerged as a key regulator of inflammation, with macrophage function and polarization being closely linked to metabolic shift.^[^ ^7^, ^8^, ^9^ ^]^ Targeting the metabolic regulatory pathways of macrophages, rather than focusing solely on their traditional immune phagocytosis function, can play a more significant role in maintaining tissue homeostasis. Lipid metabolism plays a crucial role in the activation of both M1 and M2 macrophages. Golgi apparatus is a pivotal organelle in the regulation of lipid metabolism.^[^ ^10^, ^11^ ^]^ Golgi apparatus has been proposed to be a sensor of stress and its stress response disrupts cell metabolic homeostasis.^[^ ^12^ ^]^ Golgi stress affects Golgi apparatus‐related sphingolipid metabolism in the progression of disease.^[^ ^13^ ^]^ Ceramide (CER) is a core lipid in the metabolism of a fairly large class of lipids called, sphingolipids.^[^ ^14^ ^]^ CER is synthesized in the endoplasmic reticulum, from where it is translocated to the Golgi apparatus for conversion to sphingomyelin (SM).^[^ ^15^ ^]^ However, the Golgi stress response damages the structure and function of this organelle, leading to an increase in CER levels.^[^ ^16^ ^]^ Previous studies demonstrated that dysregulated sphingolipid metabolism was intimately linked with M1 macrophage activation.^[^ ^17^, ^18^ ^]^ For instance, the accumulation of CER promotes the production of reactive oxygen species (ROS), which exacerbates inflammation.^[^ ^19^ ^]^ Additionally, Arachidonic acid (AA) is a critical lipidic metabolite and a major component of cell membrane lipids, which can be converted into various pro‐inflammatory mediators. AA is mainly metabolized by cyclooxygenase (COX) and lipoxygenase (LOX) into prostaglandin E2 (PGE2) and leukotriene B4 (LTB4), respectively. Both PGE2 and LTB4 are key mediators of the inflammatory cascade. As such, they regulate the intensity and duration of inflammation, as well as stimulate M1 macrophage activation.^[^ ^20^ ^]^ AA metabolic pathways also trigger oxidative stress during cell activation.^[^ ^21^ ^]^ Accordingly, dysregulation of sphingolipid and AA metabolism coordinates the compensatory pathways associated with ROS production and the inflammatory response during M1 macrophage activation, limiting the efficacy of the treatment regimen that modulates either of these pathways.^[^ ^22^, ^23^ ^]^ Therefore, we speculated that the simultaneously reprogramming sphingolipid and AA metabolism could promote the repolarization of M1 macrophages into the M2 phenotype. However, promoting the repolarization of M1 macrophages into the M2 phenotype through simultaneously reprogramming sphingolipid and AA metabolism remains challenging.

In recent decades, nanoparticle‐based targeting drug delivery systems have been extensively employed to safely and effectively regulate the therapeutic targets of various diseases.^[^ ^24^ ^]^ In particular, a self‐assembled nanodrug delivery system that relies on the combination of nanometer drug delivery and self‐assembly technology can deliver drugs without using drug excipients and labor‐intensive preparation protocols.^[^ ^25^ ^]^ By tailoring the structural parameters of its building blocks, a self‐assembled nanodrug delivery system can be engineered for stimuli‐responsive drug release and targeted drug delivery.^[^ ^26^ ^]^ The natural polysaccharide chondroitin sulfate (CS) preferentially targets cells with high CD44 expression levels, including M1 macrophages, due to the high affinity of CS for the CD44 receptor.^[^ ^27^ ^]^ Moreover, T. Gong's group and our lab demonstrated that CS‐based self‐assembled nanoparticles can specifically target the Golgi apparatus in cells mediated by acting as a substrate for N‐acetylgalactosaminyl transferase (GalNAc‐T).^[^ ^28^, ^29^, ^30^ ^]^ Bilirubin (BR) is a hydrophobic byproduct of broken hemoglobin, known for its ROS‐scavenging, antioxidant, and cytoprotective effects.^[^ ^31^, ^32^ ^]^ BR has been used in the construction of self‐assembled nanoparticles, where it exerts both a therapeutic effect and scavenges ROS.^[^ ^33^, ^34^ ^]^ The self‐activating function of BR‐based nanomedicines is induced by the bioreductive microenvironment, whereby the hydrophobic BR portion is converted to the hydrophilic biliverdin on exposure to ROS,^[^ ^35^, ^36^ ^]^ bringing about the disassembly of nanoparticles to release the encapsulated drug. Therefore, we hypothesized that BR‐modified CS could form the basis of a self‐assembling nanocarrier to target the CD44‐overexpressing M1 macrophages and scavenge the intracellular ROS after reaching the Golgi apparatus. Nevertheless, the potential of this self‐assembling nanocarrier for metabolic reprograming in M1 macrophage has been barely explored.

**Figure 1:**
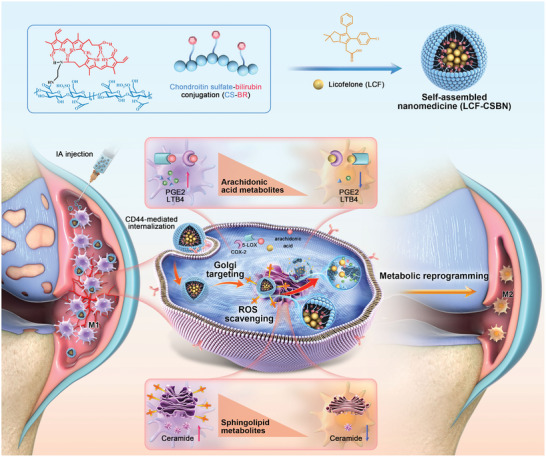
Schematic representation of LCF‐CSBN targeting the Golgi apparatus in M1 macrophages and promoting M1 macrophages to M2 phenotype by reprogramming sphingolipid and arachidonic acid metabolism.

Here, we report a self‐assembled nanocarrier (designated as CSBN) based on CS‐BR conjugate for the targeting delivery of licofelone (LCF), a dual inhibitor of COX‐2 and 5‐LOX.^[^ ^37^, ^38^ ^]^ The potent antioxidant BR, as a hydrophobic moiety, was modified on the CS polymer chain via an ethylenediamine (EDA) linker to yield CS‐BR conjugate. The resulting amphiphilic polymer could then self‐assembled as a nanocarrier for the efficient encapsulation of LCF. LCF loaded CSBN (LCF‐CSBN) was intra‐articularly injectable and prolonged the retention time of LCF in the joints. After being internalized by M1 macrophages via CD44‐mediated endocytosis, LCF‐CSBN was selectively delivered to the Golgi apparatus. Once inside this organelle, LCF‐CSBN alleviated the Golgi stress by a BR‐mediated ROS scavenging mechanism, contributing to the reprogramming of Golgi apparatus‐related sphingolipid metabolism. Subsequently, the released LCF could bind to the intracellular COX‐2 and 5‐LOX, leading to the reprogramming of AA metabolism. Accordingly, LCF‐CSBN could effectively enhance M1 to M2 phenotypic transition of macrophages, thereby suppressing low‐grade inflammation in the OA joint (**Figure** **1**). RNA sequencing, lipidomic, and enzyme‐linked immunosorbent assay (ELISA) revealed that LCF‐CSBN repolarized macrophage from the M1 to the M2 phenotype by decreasing the levels of PGE2, LTB4, and CER, as well as by inhibiting inflammatory signaling pathways. Finally, the therapeutic efficacy and safety profiles of LCF‐CSBN were confirmed in two animal models of OA, whereby we showed that LCF‐CSBN improved OA pathology by suppressing joint inflammation and alleviating cartilage damage. In summary, the constructed multifunctional nanomedicine LCF‐CSBN represents a promising strategy for treating joint‐related degenerative diseases such as OA.

## 2 Results

### 2.1 Golgi Stress is Positively Associated with OA Synovial Inflammation

**Figure 2:**
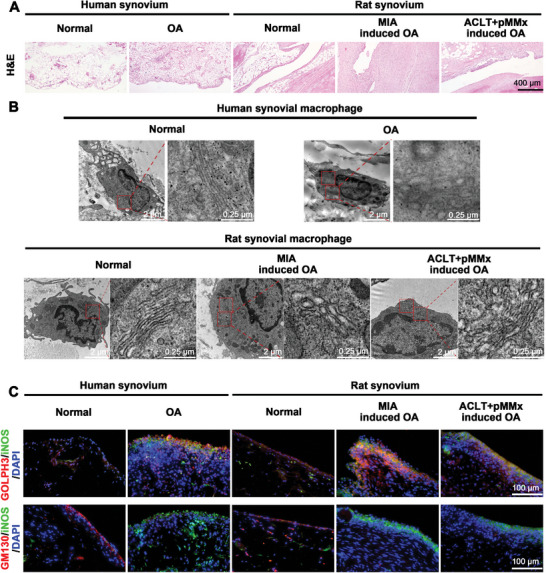
M1 macrophages accumulate in OA synovium, accompanying with Golgi stress, expressing high levels of GOLPH3 and low levels of GM130. A) Representative H&E staining images of synovial tissues from healthy controls, OA patients and OA rats. B) Representative TEM images of the Golgi apparatus in synovial macrophages from healthy controls, OA patients and OA rats, red box indicates the Golgi apparatus. C) Representative coimmunostaining images of GOLPH3 or GM130 in synovial M1 macrophages from human and rats, synovial M1 macrophages were stained with iNOS antibody.

Excessive accumulation and M1 polarization of macrophages in synovial tissues contribute to the progression of OA by triggering inflammation.^[^ ^3^, ^4^ ^]^ In this study, hematoxylin and eosin (H&E) staining revealed that both OA patients and OA rats had significant synovial inflammation that was not present in healthy controls and normal rats (**Figure** **2**A). Furthermore, morphological structures of the Golgi apparatus that were observed through transmission electron microscopy (TEM) exhibited significant swelling in synovial macrophages of OA patients compared to those in synovial macrophages of healthy controls (Figure 2B). Moreover, similar morphological changes in Golgi apparatus were displayed in synovial macrophages of OA rats (Figure 2B). A previous study has shown that M1 macrophages coincided with an upregulated expression of Golgi phosphoprotein 3 (GOLPH3), a stress‐inducible Golgi membrane protein with heightened expression indicative of Golgi stress.^[^ ^39^ ^]^ Notably, the knockdown of GOLPH3 effectively reduced the inflammatory response of M1 macrophages.^[^ ^39^ ^]^ Besides, decreased expression of Golgi matrix protein 130 (GM130) was reported under oxidative stress.^[^ ^40^, ^41^ ^]^ According to previous studies, inducible nitric‐oxide synthase (iNOS) is a classical marker of M1 macrophages,^[^ ^42^, ^43^ ^]^ iNOS‐positive cells in synovium were recognized as synovial M1 macrophages. Therefore, we conducted immunofluorescence (IF) staining to investigate the protein levels of GOLPH3 and GM130 coimmunostaining with iNOS in the synovium of OA patients and healthy controls. The IF staining results indicated a higher presence of M1 macrophages in OA synovium compared to the normal synovium, aligning with previous studies.^[^ ^3^, ^4^ ^]^ Additionally, there was a notable increase in the protein level of GOLPH3 (Figure 2C; Figure S1A,B, Supporting Information) and reduction in the protein level of GM130 (Figure 2C; Figure S2A,B, Supporting Information) expressed by these M1 macrophages in the OA synovium. Additionally, elevated protein levels of GOLPH3 (Figure 2C; Figure S1C,D, Supporting Information) and decreased protein levels of GM130 (Figure 2C; Figure S2C,D, Supporting Information) were expressed by M1 macrophages in synovium of both monosodium iodoacetate (MIA) rats and anterior cruciate ligament transection plus partial medial meniscectomy (ACLT+pMMx) rats compared to normal rats, paralleling the results in M1 macrophages within human synovium. These findings indicate that Golgi stress exists in synovial M1 macrophages and that is positively associated with synovial inflammation in OA.

### 2.2 Preparation and Characterization of LCF‐CSBN

**Figure 3:**
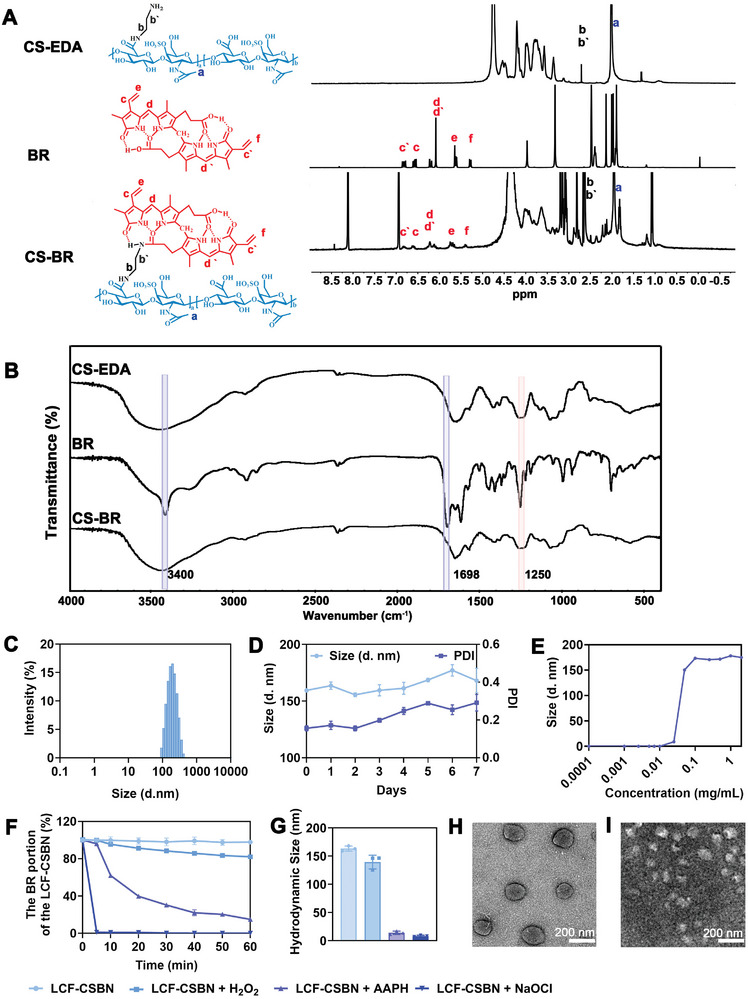
Physicochemical characterization and ROS‐mediated decomposition of LCF‐CSBN. A) The ^1^H NMR spectra of CS‐EDA, BR, and CS‐BR. B) The FTIR spectra of CS‐EDA, BR, and CS‐BR. C) Size distribution of LCF‐CSBN. D) The storage stability of LCF‐CSBN, hydrodynamic size and PDI changes of LCF‐CSBN in PBS at 4 °C (*n* = 3, mean ± SD). E) The critical micelle concentrations of CSBN. F) Changes in BR portion of LCF‐CSBN induced by ROS stimuli. LCF‐CSBN in PBS incubated with 5 mM of H_2_O_2_, 100 mM of AAPH, and 1 mM of NaOCl, respectively (*n* = 3, mean ± SD). G) The hydrodynamic sizes of LCF‐CSBN when exposed to 5 mM of H_2_O_2_, 100 mM of AAPH or 1 mM of NaOCl for 1 h, respectively (*n* = 3, mean ± SD). H,I) Representative TEM images of LCF‐CSBN (H) and LCF‐CSBN incubated with 100 mM of AAPH (I) for 1 h.

We constructed a CSBN nanoplatform by self‐assembling the amphiphilic block conjugated by CS and BR with ethylenediamine conjugate (EDA) as a linker (Figure S3, Supporting Information). The recovery of chondroitin sulfate‐ ethylenediamine conjugate (CS‐EDA) was ≈13.8%, while the yield of CS‐BR was ≈68.7%, with a purity of ≈93.7% (Figure S4, Supporting Information). The successful synthesis of CS‐BR was confirmed by the proton magnetic resonance spectrum (^1^H NMR), and Fourier transform infrared spectrophotometer (FTIR). The characteristic proton signals around the chemical shift of 1.92 ppm and 3.27 to 4.55 ppm in the CS structure were assigned to N‐acetyl group and the sugar rings, meanwhile, the typical peaks of terminal alkenes in BR structure were distributed from 5.28 to 6.87 ppm, which were presented in the ^1^H NMR spectrum of CS‐BR (**Figure** **3**A). Moreover, the FTIR spectrum of CS‐BR displayed the obvious absorption peak at 1250 cm^−1^ assigned to S‐O stretching vibration of CS and the characteristic peaks at 3400 and 1698 cm^−1^ belonged to the N‐H stretch band in the pyrrole ring and the carboxyl group of BR (Figure 3B).

CSBN was applicated to deliver hydrophobic LCF, harvesting the nanomedicine termed LCF‐CSBN, and the control nanomedicine LCF‐loaded polyethylene glycolylated (PEGylated) BR was termed as LCF‐PEGBN (Table S1 and Figure S5, Supporting Information). According to the results of the dynamic light scattering (DLS) assay (Figure 3C; Table S1, Supporting Information), the particle size of LCF‐CSBN was 163 ± 3.29 nm with a uniform size distribution, of which the polydispersity index (PDI) was 0.176 ± 0.019. Zeta potential of LCF‐CSBN was −35.3 ± 3.30 mV. Additionally, the morphology of LCF‐CSBN was observed by TEM, which revealed a generally spherical shape with good monodispersity (Figure 3H). Drug loading efficiency and encapsulation efficiency of LCF in LCF‐CSBN were ≈4.75 ± 0.76% and 94.3 ± 2.74% (Table S1, Supporting Information). Moreover, LCF‐CSBN was found stable over a 7 days storage period in phosphate‐buffered saline (PBS) at 4 °C, with marginal increase in size and PDI (Figure 3D). The critical micelle concentration (CMC) of CSBN was ≈25 µg mL^−1^ (Figure 3E). The content of BR in LCF‐CSBN dropped after exposure to ROS (Figure 3F), especially in the presence of NaOCl or 2,2′‐azobis(2‐amidinopropane) dihydrochloride (AAPH). Particle size of LCF‐CSBN was changed from 163 nm to ≈10 nm (Figure 3G) determined by DLS. The change in LCF‐CSBN size in the presence or absence of AAPH was confirmed by TEM (Figure 3I), indicating that LCF‐CSBN rapidly dissociated upon exposure to ROS. Moreover, the portion of BR in LCF‐CSBN showed a slight decrease during a 7‐day incubation with synovial fluid from OA patients, suggesting no obvious disassembly of nanoparticles in OA microenvironment (Figure S6, Supporting Information). Additionally, the drug release profile in Figure S7 (Supporting Information) revealed that LCF‐CSBN exhibited a sustained release for LCF in PBS, but more rapidly release in the presence of H_2_O_2_, indicating that LCF‐CSBN promotes ROS‐triggered rapid drug release.

### 2.3 LCF‐CSBN Targets the Golgi Apparatus after Being Internalized by M1 Macrophages

**Figure 4:**
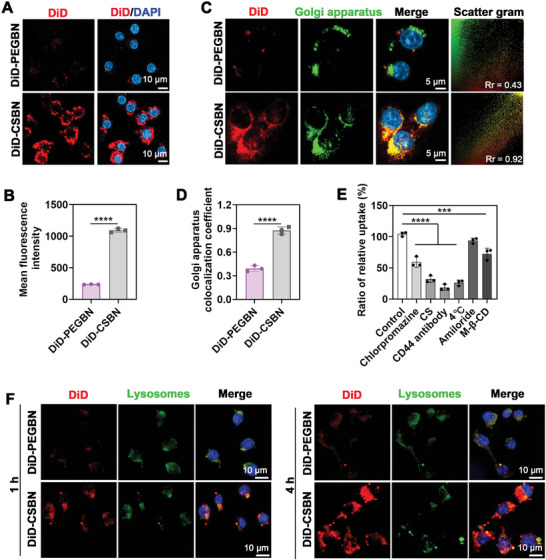
LCF‐CSBN accumulates in the Golgi apparatus after being internalized by M1 macrophages. A) Representative confocal images indicated the cellular uptake of DiD‐PEGBN or DiD‐CSBN in M1 macrophages. B) Flow cytometric analysis of cellular uptake in M1 macrophages (*n* = 3, mean ± SD). C) Representative colocalization images of DiD‐labeled nanomedicines with the Golgi apparatus in M1 macrophages. D) Colocalization efficiency between DiD‐labeled nanomedicines and the Golgi apparatus presented as Pearson's correlation coefficient (*n* = 3, mean ± SD). E) Endocytosis pathway analysis of DiD‐CSBN in M1 macrophages (*n* = 3, mean ± SD). F) Representative fluorescence images of DiD‐labeled nanomedicines and lysosomes in M1 macrophages at 1 and 4 h. *** *p* < 0.001, **** *p* < 0.0001, as determined by student's two‐sided t test (B and D) or one‐way ANOVA with Tukey's post hoc test (E).

To investigate the targeting potential of LCF‐CSBN to M1 macrophages, confocal imaging and flow cytometry were conducted. In these assays, CSBN was labeled with the fluorescent probe 1,1′‐dioctadecyl‐3,3,3′,3′‐tetramethyl indodicarbocyanine, 4‐chlorobenzenesulfonate salt (DiD) to yield DiD‐CSBN. Meanwhile, PEG, as a hydrophilic polymer material, is not the substrate of CD44 receptor and has been usually used as the control for the other hydrophilic polymer materials in the CD44‐mediated cell uptake test.^[^ ^44^, ^45^ ^]^ Confocal imaging showed a substantial increase in the red fluorescence signal in M1 macrophages co‐cultured with DiD‐CSBN for 4 h (**Figure** **4**A). This outcome was corroborated by flow cytometry, highlighting the enhanced cellular uptake of DiD‐CSBN in M1 macrophages compared to DiD‐PEGBN (Figure 4B; Figure S8, Supporting Information). Moreover, flow cytometry analysis revealed a greater cellular uptake of DiD‐CSBN by M1 macrophages compared to M0 macrophages, M2 polarized macrophages and activated fibroblasts (Figure S9, Supporting Information).

Our previous study has found that CS‐modified nanoparticles have a strong affinity to the Golgi apparatus in tumor cells.^[^ ^30^ ^]^ Subsequently, we conducted IF staining to explore the Golgi apparatus‐targeting capability of LCF‐CSBN in M1 macrophages. As shown in Figure S10 (Supporting Information), the green fluorescence signals of endoplasmic reticulum tracker or mitochondria tracker were obviously separated from the red fluorescence of DiD‐CSBN, demonstrating that LCF‐CSBN was not preferentially localized in these organelles. However, the confocal imaging results showed negligible accumulation of DiD‐PEGBN in the Golgi apparatus. By contrast, the considerable overlap of DiD‐CSBN (red) and Golgi apparatus (green) fluorescence signal was observed in M1 macrophages treated with DiD‐CSBN (Figure 4C). The statistical correlation between the green and red fluorescence signal was presented by color scatter plots and corresponding Pearson's correlation coefficient (PCC) (Figure 4D). LCF‐CSBN had higher PCC value than LCF‐PEGBN, indicating its effective targeting potential of the Golgi apparatus in M1 macrophages.

Additionally, an endocytosis inhibition experiment was conducted to elucidate the internalization pathway of LCF‐CSBN in M1 macrophages. The results indicated that the cellular uptake of DiD‐CSBN in M1 macrophages was significantly inhibited by pre‐treatment with 4 °C, CS and CD44 antibody, suggesting that LCF‐CSBN endocytosis is temperature‐dependent and predominantly mediated by CD44 receptors (Figure 4E). We further assessed the time‐dependent colocalization of LCF‐CSBN with lysosomes. The results revealed a high colocalization of DiD‐CSBN and DiD‐PEGBN with lysosomes in M1 macrophages after 1 h of cell culture. However, after 4 h of cell culture, DiD‐CSBN exhibited reduced colocalization with lysosomes compared to DiD‐PEGBN, suggesting that LCF‐CSBN possesses a degree of lysosomal escape capacity (Figure 4F; Figure S11A,B, Supporting Information).

### 2.4 LCF‐CSBN Effectively Mitigates Golgi Stress in M1 Macrophages

**Figure 5:**
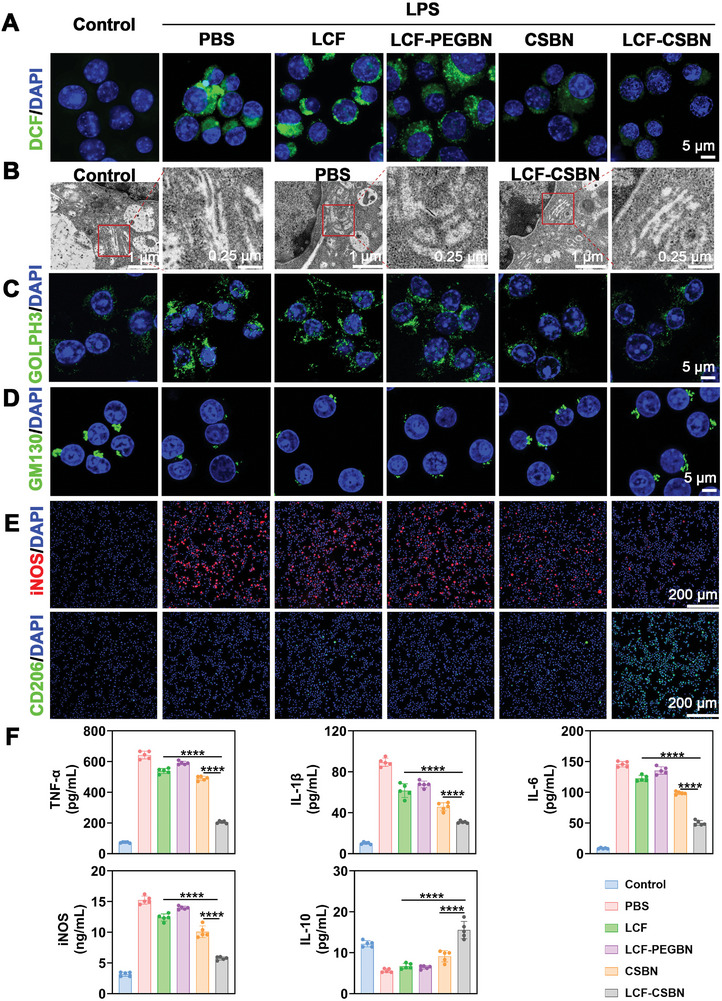
LCF‐CSBN alleviates Golgi stress and repolarizes M1 macrophages to M2 phenotype. A) Intracellular ROS stained by DCFH‐DA (green) in M1 macrophages with different treatments. B) Representative TEM images of the Golgi apparatus in M1 macrophages incubated with PBS and LCF‐CSBN for 24 h, red box indicates the Golgi apparatus. C,D) Representative confocal images of GOLPH3 (C) and GM130 (D) in M1 macrophages from different groups. E) Representative coimmunostaining images of iNOS (M1 phenotype) and CD206 (M2 phenotype) illustrating the repolarization efficiency in macrophages with different treatments. F) The protein levels of TNF‐α, IL‐1β, IL‐6, iNOS, and IL‐10 in the supernatant of M1 macrophages with different treatments were detected by ELISA assay (*n* = 5, mean ± SD). **** *p* < 0.0001, as determined by one‐way ANOVA with Tukey's post hoc test (F).

ROS‐induced Golgi apparatus oxidative stress can impair its morphology and function.^[^ ^46^ ^]^ BR, a bioactive moiety of CS‐BR, was demonstrated to have potent intracellular ROS‐scavenging capacity.^[^ ^47^ ^]^ Hence, we performed the 5‐(and‐6)‐chloromethyl‐2′,7′‐dichlorofluorescein diacetate (DCFH‐DA) assay to evaluate the ROS‐scavenging property of LCF‐CSBN in M1 macrophages. A notable decrease in intracellular ROS levels was observed in M1 macrophages treated with LCF‐CSBN (**Figure** **5**A; Figure S12A, Supporting Information), indicating the effective intracellular ROS scavenging by LCF‐CSBN.

Furthermore, we investigated the capacity of LCF‐CSBN to mitigate Golgi stress in M1 macrophages. Therefore, we conducted TEM assay and IF staining for GOLPH3 and GM130. In line with previous study,^[^ ^40^ ^]^ the Golgi apparatus in PBS‐treated M1 macrophages exhibited dilated and swollen cisternae, whereas the Golgi apparatus of LCF‐CSBN‐treated M1 macrophages displayed flat cisternae, which were the least swollen among the treatment groups (Figure 5B; Figure S13, Supporting Information). Consistent with the results of intracellular ROS detection experiments in M1 macrophages, the green signal of GOLPH3 was significantly reduced in M1 macrophages treated with LCF‐CSBN (Figure 5C; Figure S12B, Supporting Information), and an enhanced fluorescence signal of GM130 in M1 macrophages treated with LCF‐CSBN was observed (Figure 5D; Figure S12C, Supporting Information). Collectively, these findings indicate that LCF‐CSBN effectively reduced the intracellular ROS levels and mitigated Golgi stress in M1 macrophages.

### 2.5 LCF‐CSBN Regulates M1 Macrophages Repolarization In Vitro

Intracellular ROS production and Golgi stress play crucial roles in lipid metabolism. Furthermore, previous studies have established an association between lipid metabolism and macrophage polarization.^[^ ^11^, ^48^ ^]^ Since we established that LCF‐CSBN could scavenge ROS and alleviate Golgi stress, we hypothesized that it could repolarize M1 macrophages. Therefore, we conducted flow cytometry by utilizing bone marrow‐derived macrophages (BMDMs) as the model cells for macrophage repolarization study, and the result of lipopolysaccharide (LPS) stimulated BMDMs demonstrated that M1 macrophages were substantially transformed to M2 phenotype by LCF‐CSBN (Figure S14, Supporting Information). Subsequently, we investigated the repolarization of LPS stimulated RAW 264.7 cells treated with PBS, LCF, LCF‐PEGBN, CSBN, or LCF‐CSBN. The IF staining results showed that LCF, LCF‐PEGBN, and CSBN barely decreased the expression of iNOS and modestly increased the expression of CD206 in LPS‐stimulated RAW 264.7 cells. In contrast, a significant decrease in the iNOS fluorescence signal and a notable increase in the CD206 fluorescence signal were observed in LPS‐stimulated RAW 264.7 cells treated with LCF‐CSBN (Figure 5E). Subsequent ELISA results demonstrated that M1 macrophages treated with LCF, LCF‐PEGBN, or CSBN secreted slightly lower levels of pro‐inflammatory cytokines, including tumor necrosis factor‐alpha (TNF‐α), interleukin‐1 beta (IL‐1β), interleukin‐6 (IL‐6), and iNOS than those treated with PBS. In particular, LCF‐CSBN exhibited the most substantial effect on reducing the secretion of pro‐inflammatory cytokines among all treatment groups. Moreover, the increased secretion of IL‐10, an anti‐inflammatory cytokine, was observed only in LCF‐CSBN‐treated M1 macrophages among all treatment groups (Figure 5F). Additionally, results of quantitative real‐time polymerase chain reaction (qRT‐PCR) demonstrated that only LCF‐CSBN could robustly suppress the mRNA expressions of pro‐inflammatory cytokines and stimulate the mRNA expression of anti‐inflammatory cytokine (IL‐4) in M1 macrophages among all treatment groups (Figure S15, Supporting Information). Notably, compared with single or dual inhibitor of COX‐2 or 5‐LOX, LCF exhibited stronger ability to induce the repolarization of M1 macrophages to M2 macrophages (Figures S16 and S17, Supporting Information). Previous studies revealed that the fibroblast growth factor receptor 1 (FGFR1) was essentially involved in macrophage activation and macrophage‐specific FGFR was associated with lipid metabolism.^[^ ^49^, ^50^ ^]^ Decreasing the expression of FGFR1 could reduce lipid accumulation in macrophages.^[^ ^49^, ^50^, ^51^ ^]^ The mRNA expression of FGFR1 in M1 macrophages treated with LCF were significantly lower than those in M1 macrophages treated with PBS (Figure S18, Supporting Information). Immunofluorescence staining results showed that the protein expressions of FGFR1 and p‐FGFR1 were significantly decreased in M1 macrophages treated with LCF (Figure S19, Supporting Information). Additionally, LCF significantly reduced the production of phosphatidic acid (PA) in M1 macrophages (Figure S20, Supporting Information). PA, as a key intermediate in lipid metabolism, can induce rapid production of proinflammatory cytokines by macrophages.^[^ ^52^, ^53^, ^54^ ^]^ Moreover, reduced expression levels of FGFR1 and p‐FGFR1 were observed in synovial macrophages in two OA rat models treated with LCF. LCF‐CSBN was more efficient in regulating the production of these proteins (Figures S21 and S22, Supporting Information). In addition to restoring the AA metabolism by acting on COX‐2 and 5‐LOX, LCF regulates lipid metabolism partially through decreasing FGFR1 expression, which may contribute to the repolarization of M1 macrophages. Taken together, these results demonstrate that LCF‐CSBN effectively repolarizes M1 macrophages toward the M2 phenotype. This might be attributed to the ROS scavenging ability of CSBN, which alleviates Golgi stress, as well as the better therapeutic effects of LCF compared to single or dual inhibitor of COX‐2 and 5‐LOX.

### 2.6 LCF‐CSBN Reprograms Lipid Metabolism and Inhibits Inflammation in M1 Macrophages

**Figure 6:**
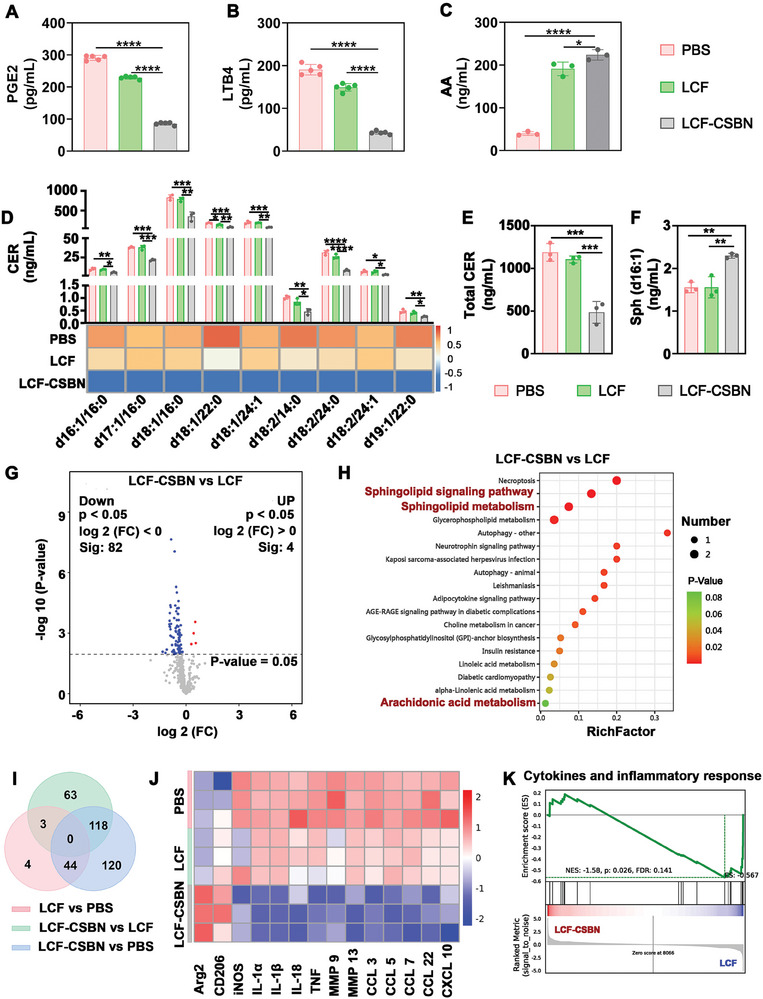
LCF‐CSBN reprograms sphingolipid and arachidonic acid metabolism and inhibits inflammatory response in M1 macrophages. A,B) Quantification of PGE2 A) and LTB4 B) levels in the supernatant of M1 macrophages in PBS, LCF, and LCF‐CSBN group by ELISA assay (*n* = 5, mean ± SD). C–F) The levels of AA C), CER metabolites D), total CER E), and Sph F) in M1 macrophages treated with PBS, LCF, and LCF‐CSBN were measured by targeted HPLC‐ESI‐MS/MS (*n* = 3, mean ± SD). G) Significantly upregulated (red) and downregulated (blue) lipid species between LCF‐CSBN group and LCF group. H) The top 20 KEGG pathways of significantly downregulated lipid species between LCF‐CSBN group and LCF group. I) Venn diagram showing the relationship and numbers of DEGs among groups. J) Heatmap showing decreased expression levels of genes encoding M1 markers and pro‐inflammatory factors, and increased expression levels of genes encoding M2 markers in macrophages treated with LCF‐CSBN compared with those of M1 macrophages treated with LCF or PBS. K) GSEA showcasing downregulation of cytokines and inflammatory response in M1 macrophages treated with LCF‐CSBN. * *p* < 0.05, ** *p* < 0.01, *** *p* < 0.001, **** *p* < 0.0001, as determined by one‐way ANOVA with Tukey's post hoc test (A‐F).

Previous studies have demonstrated that the dysregulation of lipid metabolism contributes to an imbalance in macrophage polarization.^[^ ^48^, ^55^ ^]^ As we known, AA serves as a pivotal source of pro‐inflammatory factors in OA.^[^ ^56^ ^]^ Consequently, ELISA was conducted to assess the levels of AA metabolites (specifically, PGE2 and LTB4) in the supernatant of M1 macrophages treated with PBS, LCF, or LCF‐CSBN. The ELISA results revealed that M1 macrophages treated with LCF‐CSBN produced markedly lower levels of PGE2 and LTB4 compared to those treated with PBS or LCF (**Figure** **6**A,B). Subsequently, targeted lipidomic analysis was conducted to explore the reprogramming of lipid metabolism in M1 macrophages treated with PBS, LCF, or LCF‐CSBN. Overall, a total of 502 lipid species, including phosphatidylcholine (PC), SM, phosphatidic acid (PA), CER, dihexosyl ceramide (HexCer), dihydroceramide (dhCer), etc., were detected in M1 macrophages (Figure S23, Supporting Information). Notably, the level of AA was significantly elevated in M1 macrophages treated with LCF and LCF‐CSBN, attributable to the dual inhibition of COX‐2 and 5‐LOX by LCF (Figure 6C). Meanwhile, LCF‐CSBN treatment significantly decreased CER levels (Figure 6D,E) and elevated sphingosine (Sph, the CER‐derived metabolites) levels in M1 macrophages (Figure 6F). In total, 189 and 178 individual lipid species were significantly altered in M1 macrophages treated with LCF and LCF‐CSBN, respectively, relative to those treated with PBS (Figure S24, Supporting Information). Moreover, 82 downregulated and 4 upregulated individual lipid species were observed in LCF‐CSBN treated M1 macrophages compared to those treated with LCF (Figure 6G). Kyoto Encyclopedia of Genes and Genomes (KEGG) analysis was subsequently conducted to further delineate the pathways of lipid species regulated by LCF‐CSBN. The top 20 enriched KEGG pathways associated with the downregulated lipid species in both the LCF‐CSBN versus LCF (Figure 6H) and LCF‐CSBN versus PBS (Figure S25, Supporting Information) comparisons included sphingolipid metabolism (where CER is the central core lipid) and AA metabolism. This finding indicates that LCF‐CSBN had a dramatic effect on the reprogramming of CER and AA metabolism in M1 macrophages.

To elucidate how LCF‐CSBN regulates macrophage lipid metabolism and induces a phenotypic shift from M1 to M2, we conducted RNA sequencing to analyze the gene expression patterns of M1 macrophages treated with PBS, LCF or LCF‐CSBN. A Venn diagram was used to identify the 51 and 282 significant differentially expressed genes (DEGs, *p* < 0.05 and |log_2_FoldChange| > 1) in M1 macrophages treated with LCF and LCF‐CSBN, respectively, relative to those treated with PBS (Figure 6I). Moreover, the gene expression levels of macrophage polarization markers and pro‐inflammatory factors were significantly different between the LCF‐CSBN‐treated M1 macrophages and controls (Figure 6J). For instance, we found that LCF‐CSBN‐treated M1 macrophages expressed considerably lower levels of genes encoding pro‐inflammatory factors such as TNF‐α, IL‐1α, IL‐1β, and matrix metalloproteinase 13 (MMP13) and M1 marker (i.e., iNOS), but higher levels of genes encoding M2 markers (e.g., CD206 and arginase 2). This finding indicates that LCF‐CSBN treatment inhibited the expression of pro‐inflammatory factors in M1 macrophages and repolarized M1 macrophages toward the M2 phenotype. Furthermore, downregulated DEGs in M1 macrophages treated with LCF‐CSBN was extensively enriched in the KEGG clusters described to be associated with lipid metabolism and macrophage M1 polarization in previous studies,^[^ ^57^, ^58^, ^59^ ^]^ such as mitogen‐activated protein kinase (MAPK) signaling pathway and phosphatidylinositol‐3‐kinase (PI3K) / protein kinase B (Akt) signaling pathway (Figure S26, Supporting Information). In line with the KEGG analysis findings, the results of gene set enrichment analysis (GSEA) revealed that LCF‐CSBN reduced the enrichment scores for the gene sets of “cytokines and inflammatory response”, “matrix metalloproteinases”, and “IL‐1 signaling pathway” (Figure 6K; Figure S27, Supporting Information). These results indicate that LCF‐CSBN could enhance the repolarization of M1 macrophages toward the M2 phenotype by reprogramming their lipid metabolism and inhibiting their pro‐inflammatory signaling pathways.

### 2.7 LCF‐CSBN Extends Joint Retention and Promotes M1 Macrophages Repolarization in OA Joints

**Figure 7:**
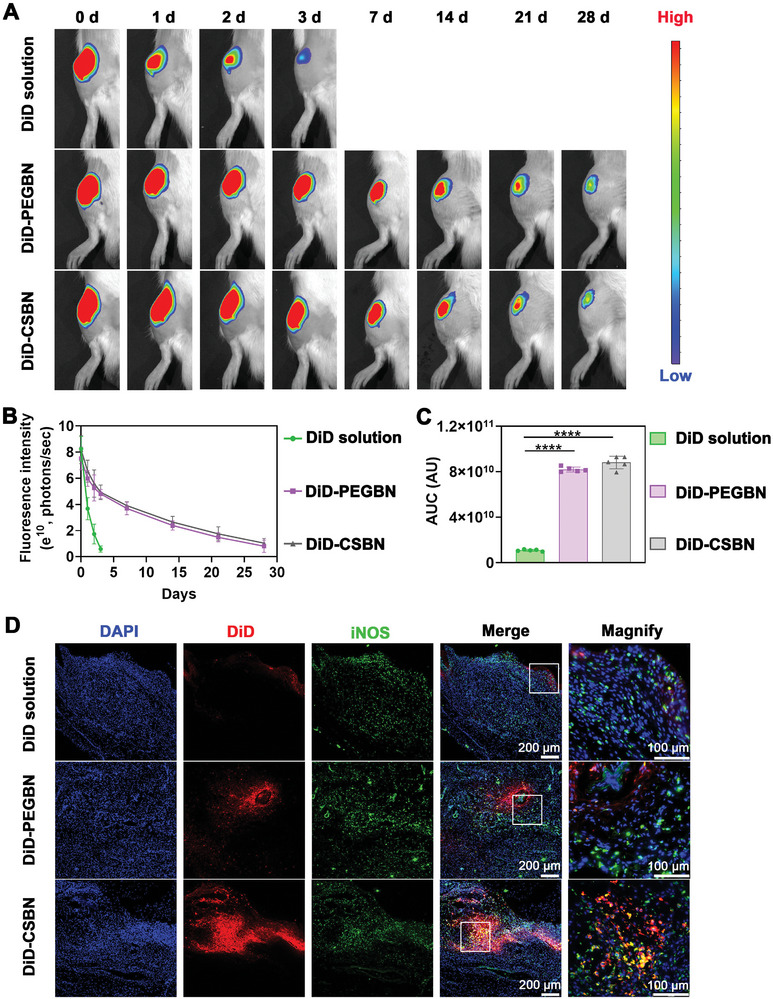
LCF‐CSBN extends residence time in joints and selectively enriches in M1 macrophages in OA synovium. A) Representative IVIS images of rat knee joints after receiving a single intra‐articular injection of DiD solution, DiD‐PEGBN, and DiD‐CSBN. B) The fluorescence intensity of knee joints based on the semi‐quantitative analysis of the IVIS images from different groups (*n* = 5, mean ± SD). C) Quantitative analysis of AUC based on fluorescence intensity results in (B) (*n* = 5, mean ± SD). D) Representative fluorescence images showing the distribution of different DiD‐labeled nanomedicines in synovial M1 macrophages, synovial M1 macrophages were stained with iNOS antibody. **** *p* < 0.0001, as determined by one‐way ANOVA with Tukey's post hoc test (C).

Intra‐articular (IA) drug delivery systems should address the problem of the short residence time due to the rapid uptake of the injected drugs within the joint cavity.^[^ ^60^, ^61^ ^]^ We assessed the retention time of DiD‐CSBN after IA injection into the OA knee joint using an in vivo imaging system (IVIS). The results demonstrated a rapid decline in fluorescence intensity for the DiD solution within 3 days after IA injection, while those for DiD‐PEGBN and DiD‐CSBN persisted for up to 28 days post‐injection (**Figure** **7**A–C).

We next determined the capacity of LCF‐CSBN to target synovial M1 macrophages through IF staining. As shown in Figure 7D, the results showed that OA rats subjected to IA injection of DiD‐PEGBN or DiD‐CSBN exhibited significantly higher fluorescence signals compared to those treated with DiD solution. Moreover, the OA synovium of rats treated with DiD‐CSBN exhibited the most pronounced colocalization of DiD with M1 macrophages among all groups, suggesting that LCF‐CSBN effectively targeted M1 macrophages in synovium of OA knee joints. Furthermore, we investigated whether LCF‐CSBN could target M1 macrophages via CD44 receptor in vivo. The colocalization of DiD and iNOS was notably lower in the synovium of OA rats pretreated with CD44 polyclonal antibody than that of OA rats only treated with DiD‐CSBN, suggesting that LCF‐CSBN targets M1 macrophages in synovium of OA rats via CD44 receptor (Figure S28, Supporting Information). Additionally, the coimmunostaining images displayed high colocalization of red, magenta and green fluorescence signals in synovium of OA rats after IA injection with LCF‐CSBN, demonstrating LCF‐CSBN accumulates in Golgi apparatus of M1 macrophages in synovium of OA rats (Figure S29, Supporting Information).

**Figure 8:**
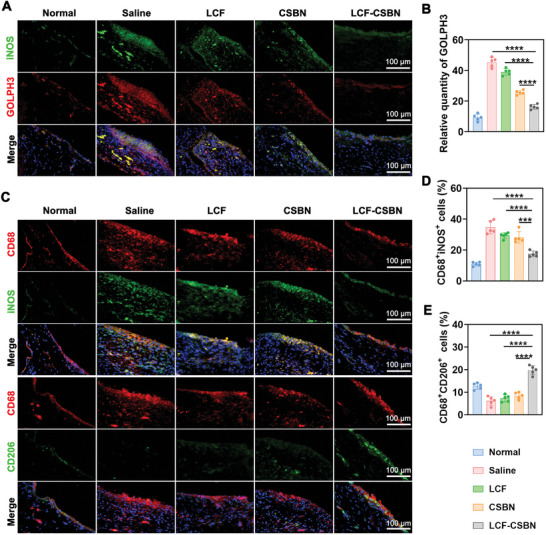
LCF‐CSBN downregulates the expression of GOLPH3 and transforms M1 macrophages into M2 phenotype in synovium of MIA rats. A) Representative coimmunostaining images of GOLPH3 in synovial M1 macrophages of MIA rats from different groups, synovial M1 macrophages were stained with iNOS antibody (green). B) Relative quantity of GOLPH3 in synovium from different groups (*n* = 5, mean ± SD). C) Representative coimmunostaining images of CD68 and iNOS or CD206 in rat synovium indicating the repolarization efficiency in synovial macrophages from different groups, M1 and M2 macrophages were stained with iNOS (green) and CD206 (green), respectively. D,E) Quantitative analysis of M1 D) and M2 E) macrophages in synovium from different groups, respectively (*n* = 5, mean ± SD). *** *p* < 0.001, **** *p* < 0.0001, as determined by one‐way ANOVA with Tukey's post hoc test (B, D, and E).

Considering the capacity of ROS scavenging and reprogramming of lipid metabolism in M1 macrophages by LCF‐CSBN in vitro, we further used L‐012 (a luminescent probe) to evaluate changes of ROS levels in OA knee joints via IVIS, and conducted IF staining to determine the expression levels of GOLPH3 and GM130. Results of IVIS demonstrated strong luminescent signals of L‐012 emitted from OA knee joints of saline‐treated rats compared to those of normal rats, LCF‐CSBN treatment considerably reduced ROS in knee joints of OA rats (Figure S30A,B, Supporting Information), indicating LCF‐CSBN had a strong ROS scavenging capacity in vivo. The IF staining revealed a reduction in GOLPH3 protein levels and an increase in GM130 protein levels in M1 macrophages in synovium of OA rats following IA injection of LCF, CSBN, or LCF‐CSBN. Notably, LCF‐CSBN exhibited the most significant reduction in GOLPH3 expression (**Figure** **8**A,B; Figure S31A,B, Supporting Information), while GM130 expression levels were considerably increased in M1 macrophages within synovium of OA rats treated with LCF‐CSBN (Figure S32A,B, Supporting Information), demonstrating LCF‐CSBN could alleviate Golgi stress in M1 macrophages in synovium of OA rats. We further conducted immunohistochemical (IHC) staining for COX‐2 and 5‐LOX (two key enzymes for AA catabolism), and IF staining of neutral sphingomyelinases (N‐SMase, an enzyme for CER synthesis) expressed by M1 macrophages in synovium of OA rats. The results revealed LCF‐CSBN had potential capacity of inhibiting COX‐2, 5‐LOX, and N‐SMase (Figure S33A–E, Supporting Information).

Having demonstrated that LCF‐CSBN effectively repolarized M1 macrophages to the M2 phenotype in vitro, we next investigated the capacity of LCF‐CSBN to repolarize M1 macrophages in synovium of OA rats. Initially, we validated a notable increase in M1 macrophage infiltration in OA synovium compared to normal synovium (Figure 8C; Figure S34A, Supporting Information). Notably, among all the treatment groups, OA rats those received IA injection of LCF‐CSBN exhibited the most significant reduction in the proportion of M1 macrophages and the most substantial increase in M2 macrophages in the synovium (Figure 8D,E; Figure S34B,C, Supporting Information). These results indicate the effective repolarization of M1 macrophages to the M2 phenotype by LCF‐CSBN in the OA synovium in vivo.

### 2.8 LCF‐CSBN Suppresses Synovial Inflammation and Relieves Pain in MIA Rats

**Figure 9:**
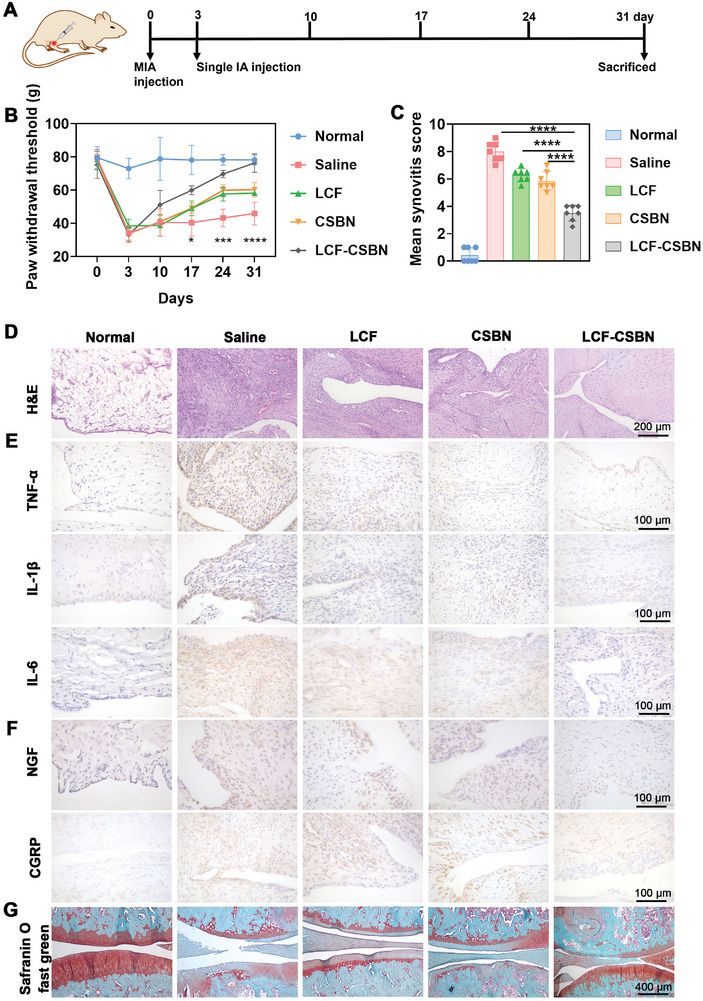
LCF‐CSBN relieves pain and suppresses synovial inflammation in MIA rats. A) Overview of the experimental set‐up with IA injections of MIA and the indicated treatments. B) Pain‐related behavior measurement using the apparatus and represented as paw withdrawal threshold (*n* = 7, mean ± SD). C) Quantification of synovitis score in synovium of MIA rats (*n* = 7, mean ± SD). D) Representative H&E staining images of synovium tissues from different groups. E,F) Representative IHC staining images of TNF‐α, IL‐1β, IL‐6, NGF, and CGRP in synovium from different groups. G) Representative Safranine O‐Fast green staining images of knee joints from different groups. * *p* < 0.05, *** *p* < 0.001, **** *p* < 0.0001, as determined by two‐way ANOVA with Tukey's post hoc test (B) or one‐way ANOVA with Tukey's post hoc test (C).

To investigate the anti‐inflammatory and analgesic effect of intra‐articularly injected LCF‐CSBN, we established a classical pain model of OA by IA injecting 8‐week‐old Sprague Dawley (SD) rats with MIA (2 mg per knee). MIA induced OA model, which is the model of choice in the pain field due to its demonstration of long‐lasting hyperalgesia and weight bearing asymmetry, has been used primarily to assess the analgesic efficacy of potentially new therapeutic agents for OA.^[^ ^62^ ^]^ Three days post MIA injection, rats received IA injections of saline, LCF, CSBN or LCF‐CSBN. Rats that not subjected to MIA and left untreated served as controls (**Figure** **9**A). Subsequent pain‐related behavior assessments were conducted to gauge the analgesic impact of LCF‐CSBN administration. Von Frey results demonstrated markedly reduced paw withdrawal threshold values in OA rats compared to controls. Notably, the low paw withdrawal thresholds were most significantly reversed in OA rats treated with IA injection of LCF‐CSBN, followed by those receiving CSBN or LCF (Figure 9B). Consistent with the von Frey results, the saline group exhibited significantly reduced spontaneous activities, while the most prominent increase was observed in the LCF‐CSBN group (Figure S35, Supporting Information). H&E staining was conducted to assess the severity of synovial inflammation in OA rats and controls. The H&E staining results showed that the histological features of synovitis (i.e., synovial tissue hyperplasia, neovascularization, and inflammatory cells infiltration) were significantly reduced in the LCF‐CSBN group, followed by the CSBN and LCF groups, compared to the saline group (Figure 9D); the synovitis scores for each group are presented in Figure 9C. Although higher synovitis scores in OA groups compared to the control group, the mean synovitis score of LCF‐CSBN‐treated group was the lowest among OA groups. Additionally, IHC staining was performed to evaluate the protein levels of pro‐inflammatory factors in synovial tissue. In line with the severity of synovial inflammation observed in OA groups, the results of IHC staining for TNF‐α, IL‐1β, and IL‐6 revealed higher proportions of pro‐inflammatory factors positive cells in saline group compared to control group. However, significantly lower proportions of these factors positive cells were detected in the LCF‐CSBN group, followed by the LCF and CSBN groups, relative to the saline group (Figure 9E; Figure S36A–C, Supporting Information). Similar results were observed in IHC staining for pain‐related factors, e.g., nerve growth factor (NGF) and calcitonin‐gene‐related peptide (CGRP) (Figure 9F; Figure S36D,E, Supporting Information). Furthermore, Safranine O‐Fast green staining was performed to assess changes in articular cartilage degradation. The results showed that although severe cartilage degradation was observed in OA rats treated with saline, cartilage integrity was most significantly improved in those treated with LCF‐CSBN among all OA rats (Figure 9G). Collectively, these results demonstrate that IA injection of LCF‐CSBN exhibited a significant and prolonged anti‐inflammatory and analgesic effects in treating MIA rats and potentially attenuated OA cartilage degradation in these animals.

### 2.9 LCF‐CSBN Attenuates Cartilage Degradation and Inhibits Osteophyte Formation in ACLT+pMMx Rats

**Figure 10:**
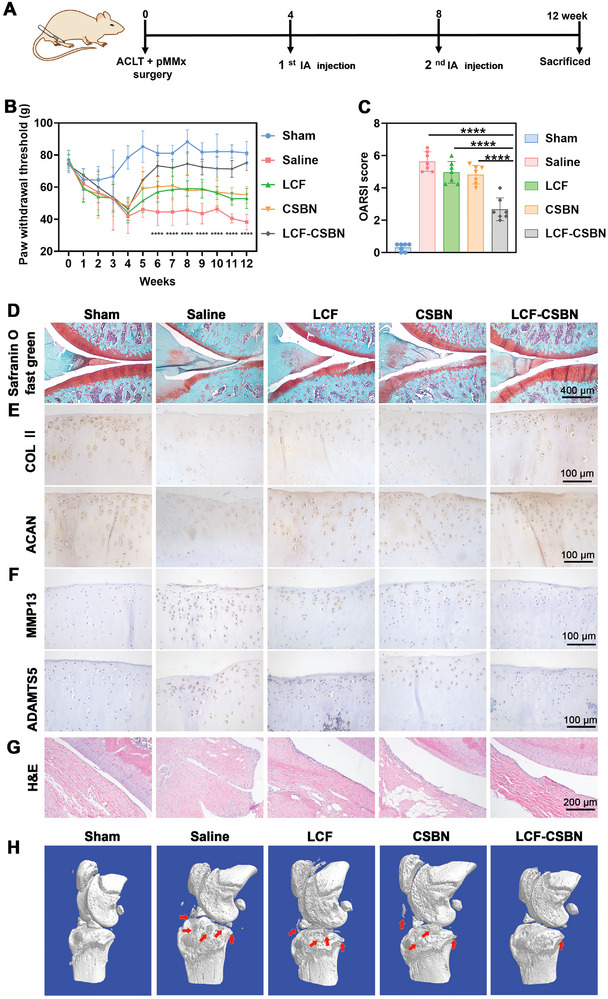
LCF‐CSBN alleviates cartilage degradation and inhibits osteophyte formation in knee joints of ACLT+pMMx rats. A) Overview of the experimental set‐up with surgery of ACLT+pMMx and IA injections of indicated treatments. B) Pain‐related behavior measurement using the von Frey apparatus and represented as paw withdrawal threshold (*n* = 7, mean ± SD). C) OARSI score of knee joints of ACLT+pMMx rats from different groups was quantitatively analyzed (*n* = 7, mean ± SD). D) Representative Safranine O‐Fast green staining images of knee joints from different groups. E,F) Representative IHC staining images of COL II, ACAN, MMP13 and ADAMTS5 in cartilage from different groups. G) Representative H&E staining images of synovium from different groups. H) µCT images of pathological structural changes in knee joints from different groups, red arrows indicate osteophytes. **** *p* < 0.0001, as determined by two‐way ANOVA with Tukey's post hoc test (B) or one‐way ANOVA with Tukey's post hoc test (C).

To further investigate the therapeutic effect of LCF‐CSBN within the knee joint, we performed ACLT+pMMx surgery to establish a surgically induced OA rat model. ACLT+pMMx induced OA is recognized as a biomechanical model of OA, characterized by a much slower progression compared to the MIA induced OA. This surgically induced OA model effectively mimics the pathogenesis of traumatic OA, including cartilage degradation, subchondral bone sclerosis, and osteophyte formation.^[^ ^63^ ^]^ Rats with 4 weeks of disease induction were stipulated as ACLT+pMMx rats with established OA according to previous studies.^[^ ^62^, ^63^, ^64^ ^]^ Hence, OA rats were received IA injections of saline, LCF, CSBN, or LCF‐CSBN twice over an 8‐week period at four weeks post the surgery, investigating treatment efficiency of LCF‐CSBN for established OA. Sham‐operated rats served as controls (**Figure** **10**A). The mechanical allodynia test was performed to evaluate the analgesic effect of IA injection of LCF‐CSBN in ACLT+pMMx rats, with consistent results compared to MIA rats (Figure 10B), highlighting the potent analgesic effect of LCF‐CSBN in OA rats, irrespective of the OA induction method. H&E and Safranine O‐Fast green staining were performed to evaluate changes in synovial tissue and cartilage. H&E staining revealed significant hyperplasia of synovial lining cells in saline group, followed by LCF and CSBN groups, while this pathological feature was significantly inhibited in LCF‐CSBN group (Figure 10G; Figure S37, Supporting Information). Meanwhile, Safranine O‐Fast green staining showed significantly higher degrees of cartilage degradation in saline group than sham group. Although IA injection of LCF or CSBN attenuated cartilage degradation to some extent, the most prominent improvement in cartilage degradation was observed in OA rats treated with LCF‐CSBN (Figure 10D). This result echoed the findings gathered from the MIA induced OA model. The osteoarthritis research society international (OARSI) score, used to quantify cartilage degradation severity, corroborated these observations (Figure 10C). Additionally, IHC staining was conducted to investigate the protective effect of LCF‐CSBN against cartilage degradation. Compared to the sham group, the saline group exhibited significantly decreased protein expression levels of synthetic factors such as type II collagen (COL II) and aggrecan (ACAN) and increased expression levels of catabolic factors such as MMP13 and a disintegrin and metalloproteinase with thrombospondin motifs 5 (ADAMTS5). However, the LCF‐CSBN group showed a marked increase in COL II and ACAN expression and a significant reduction in MMP13 and ADAMTS5 expression compared with other OA groups (Figure 10E,F; Figure S38A–D, Supporting Information). Taken together, the results of Safranine O‐Fast green and IHC staining indicate that IA injection of LCF‐CSBN played a protective role in maintaining cartilage matrix integrity in OA rats. To further explore the impact of LCF‐CSBN on bone tissue within the knee joint, micro‐computerized tomography (µCT) was conducted to evaluate osteophyte formation in the right knee joints of OA rats. We observed significantly increased osteophyte formation in saline group compared to the sham group, however, osteophyte formation was notably inhibited following IA injection of LCF‐CSBN (Figure 10H; Figures S39 and S40, Supporting Information). Collectively, these findings suggest that IA injection of LCF‐CSBN effectively relieved OA pain and delayed OA progression in rats with surgically induced OA.

### 2.10 IA Injection of LCF‐CSBN Displays Good Biocompatibility

To systematically evaluate the biocompatibility of IA injection of LCF‐CSBN, terminal deoxynucleotidyl transferase‐mediated dUTP‐biotin nick end labeling (TUNEL) assay was conducted on cartilage samples obtained from MIA rats or ACLT+pMMx rats to evaluate potential cartilage toxicity. As shown in Figures S41–S43 (Supporting Information), LCF‐CSBN exhibited the lowest proportion of apoptotic chondrocytes among all treatment groups. Additionally, we analyzed serum enzyme levels in OA rat models to evaluate the systemic toxicity of IA injection of LCF‐CSBN. The results of serum enzyme analysis showed the levels of liver function biomarkers alanine transaminase (ALT) and aspartate aminotransferase (AST), the kidney function biomarker, blood urea nitrogen (BUN), and the heart function biomarker, lactate dehydrogenase 1 (LDH1), in the serum of OA rats treated with saline, LCF, CSBN or LCF‐CSBN were comparable to those of rats in the control group (Figures S44A and S45A, Supporting Information). Furthermore, histological analysis of the major organs (including heart, spleen, liver, lung, and kidney) in OA rats treated with saline, LCF, CSBN or LCF‐CSBN indicated no obvious toxicity (Figures S44B and S45B, Supporting Information). Additionally, there was no significant cytotoxicity of LCF‐CSBN at concentrations of LCF equivalents ranging from 0.0098−2.5 µg mL^−1^ (Figure S46, Supporting Information), demonstrating LCF‐CSBN exhibits good biocompatibility. Therefore, these results demonstrate that IA injection of LCF‐CSBN displayed favorable biocompatibility in vivo.

## 3 Discussion

Increased M1 macrophage polarization promotes chronic low‐grade inflammation, leading to the progression of age‐related degenerative diseases such as OA.^[^ ^4^, ^65^, ^66^ ^]^ Cellular lipid metabolism plays a critical role in macrophage polarization.^[^ ^1^, ^55^, ^67^ ^]^ Moreover, OA is considered a metabolic disease, and a lipid metabolic disorder was observed in OA samples.^[^ ^68^ ^]^ AA, as the main source of pro‐inflammatory mediators (e.g., PGE2 and LTB4), could trigger M1 macrophage activation. Besides, previous studies revealed that elevated CER level was associated with macrophage M1 polarization.^[^ ^17^, ^69^, ^70^ ^]^ Therefore, we proposed that repolarizing M1 macrophages toward the M2 phenotype by reprogramming lipid metabolism could be a promising therapeutic strategy for delaying the progression of OA.

The Golgi apparatus plays a vital role in lipid metabolism, as it is the site of key enzymes (e.g., CER kinase) implicated in lipid metabolism and orchestrates the transport of lipids across membranes.^[^ ^10^, ^11^, ^71^ ^]^ Activating macrophages with LPS induces excessive ROS production, which disrupts the structure and function of the Golgi apparatus in a process, termed Golgi stress.^[^ ^13^ ^]^ We also showed that the expression of GOLPH3, a Golgi stress‐inducible protein, was increased in M1 macrophages. Besides, the synovial macrophages of OA patients and OA model rats displayed high GOLPH3 expression. Previous studies revealed that Golgi stress disrupted sphingolipid metabolism, as indicated by the elevated CER levels in cells.^[^ ^15^, ^17^ ^]^ In the present study, we also found that the CER levels were significantly elevated in M1 macrophages. We therefore synthesized a CS‐BR conjugate using an EDA linker, to generate a self‐assembling nanocarrier, potentially capable of delivering LCF to the Golgi apparatus in M1 macrophages, alleviating Golgi stress, and restoring lipid homeostasis. The resulting LCF‐CSBN was equipped with the CD44‐binding and Golgi‐apparatus‐targeting properties of CS as well as the ROS‐scavenging function of BR. According to previous reports,^[^ ^28^, ^29^, ^72^ ^]^ CS‐modified nanocarriers are potential Golgi‐specific drug delivery system. The subcellular distribution of LCF‐CSBN demonstrated that it did not preferentially accumulate in mitochondria or the endoplasmic reticulum, but instead localized specifically in the Golgi apparatus. Moreover, we found that LCF‐CSBN could partially escape from the lysosomes, which might be attributed to the caveolin‐dependent endocytosis mechanism.^[^ ^73^, ^74^ ^]^ The decreased lysosomes distribution of nanomedicines could improve the therapy outcome by protecting loaded drug from exposure to acidic pH and a hydrolytic environment.^[^ ^75^ ^]^ Accordingly, LCF‐CSBN effectively reduced intracellular ROS production and Golgi stress, while improving the efficacy of LCF in attenuating M1 macrophage activation. The lipidomic analysis results showed that the lipid metabolism in M1 macrophages (especially sphingolipid and AA metabolism) was significantly regulated by LCF‐CSBN. We found that LCF‐CSBN treatment significantly reduced the levels of CER and AA metabolites (i.e., PGE2, LTB4) while increasing that of AA. Therefore, LCF‐CSBN not only attenuated oxidative stress in the Golgi apparatus in M1 macrophages but also facilitated the lipid metabolic reprogramming of M1 macrophages toward M2 phenotype.

Due to OA is typically confined to one or a few joints, local IA therapies bring several advantages over systemic drug administrations, including increased local bioavailability, reduced systemic drug exposure, and a lower risk of systemic side effects.^[^ ^60^, ^61^ ^]^ Therefore, IA injection has become a widely adopted approach in both the clinical treatment of knee OA and preclinical studies.^[^ ^76^, ^77^ ^]^ Nevertheless, drug retention time in the joint cavity greatly contributes to treatment variability. Intra‐articularly injected therapies are cleared from the synovial fluid by small blood vessels and the lymphatic system at a rate which is likely influenced by the molecular size of the injected therapeutic agent. Because IA injection is an invasive procedure, which may cause the patients discomfort and introduce infection into the joint, strategies that prolong the retention of the injected drug in the joint cavity are needed to reduce the frequency of IA injection. However, the retention time of conventional drugs used in OA treatment is short, ranging from a few hours to several days. In accordance, we observed that the IA injection of the aqueous LCF formulation had a short retention time in the joint cavity, as the drug entered blood circulation immediately after injection; thus, frequent injections and increased dosages of the drug would be required to maintain the desired therapeutic effect. Nanoparticle‐based drug delivery systems have been designed to prolong drug retention in the joint. Previous studies using in vivo imaging to determine the retention of IA‐injected sustained release formulations in the joints reported retention times ranging from several days to 28 days.^[^ ^78^, ^79^, ^80^ ^]^ In the present study, the LCF‐CSBN was retained in the joint for up to 28 days. This is likely because its relatively large size (≈160 nm) prevented the rapid clearance from synovial tissue via blood vessels and the lymphatic system. Additionally, drawing inspiration from the recently reported inflammatory‐cell‐mediated sequestration mechanism identified in the arthritic joint,^[^ ^81^ ^]^ we speculate that the selective endocytosis of LCF‐CSBN by M1 macrophages could also be responsible for its prolonged retention in the joints. Thus, the favorable pharmacokinetics of LCF‐CSBN could reduce the frequency of IA injections, which may improve OA treatment outcomes and increase patient compliance.

Structural alterations in the articular cartilage and subchondral bone are involved in OA progression, eventually leading to disability.^[^ ^82^ ^]^ Unfortunately, a disease‐modifying treatment for OA is not yet available as the pharmacological agents for OA therapy only relieve pain in a short time and hardly protect against cartilage degeneration at the same time.^[^ ^83^ ^]^ By contrast, we showed that IA injection of LCF‐CSBN significantly relieved both joint pain and synovial inflammation for nearly a month in rats with MIA or ACLT+pMMx induced OA. Additionally, LCF‐CSBN effectively attenuated cartilage damage in OA rats by increasing the expression of the chondrocyte anabolic markers and decreasing the expression of the chondrocyte catabolic markers. Importantly, LCF‐CSBN was markedly more effective at improving OA symptoms and tissue morphology than either LCF or CSBN. The superior efficacy of LCF‐CSBN over either LCF or CSBN alone can likely be attributed to the synergy between the biological nanocarrier CSBN, which effectively delivers LCF to its target site and increases its retention, and the pharmacological agent LCF, which simultaneously reprograms AA and sphingolipid metabolism in M1 macrophages. Therefore, LCF‐CSBN effectively repolarizes synovial macrophages from M1 to M2 phenotypes and suppresses synovial inflammation, which may ameliorate cartilage degeneration by reduction the amounts of pro‐inflammatory cytokines and cartilage‐degrading enzymes secreted by M1 macrophages.^[^ ^84^, ^85^ ^]^ Moreover, LCF‐CSBN treatment induced no obvious systemic or cartilage toxicity, likely because the mode of IA drug delivery reduces systemic exposure and the CSBN specifically target M1 macrophages. Consequently, the LCF‐CSBN designed and tested in the present study represent a promising disease‐modifying drug candidate in OA.

In this study, we constructed CSBN from the biocompatible and biodegradable CS and BR moieties. CS has already been approved in the clinical treatment of OA, while BR is an endogenous antioxidant with a well‐characterized safety profile. Although LCF‐CSBN demonstrated good therapeutic efficacy and biosafety in rats with chemically or surgically induced OA, scale‐up tests for preparing LCF‐CSBN for translation into the clinic and evaluating their long‐term toxicity are warranted. Besides, the efficacy of LCF‐CSBN should be further evaluated in knockout models (e.g., CD44 knockout rats), which will be crucial in the clinical translation of this promising nanomedicine. Furthermore, we simply categorized macrophages into M1/M2 phenotypes. The future clinical application of LCF‐CSBN warrants a more precise identification of macrophages subtypes, thus helping to clarify the roles of different subtypes of these macrophages in the treatment of OA.

## 4 Conclusion

In this study, we developed an injectable nanomedicine platform called LCF‐CSBN. This platform demonstrated efficient suppression of synovial inflammation and mitigation of cartilage degradation by reprogramming of synovial M1 macrophages' lipid metabolism in OA. LCF‐CSBN exhibited remarkable efficacy in targeting the Golgi apparatus in M1 macrophages and subsequently reinstating homeostasis in sphingolipid and AA metabolism, ultimately transforming M1 macrophages into the M2 phenotype in vitro. In addition, LCF‐CSBN presented extended joint‐retention time and effectively relieved OA pain, attenuated synovitis, and delayed cartilage degeneration in both MIA induced and surgically induced OA models. Furthermore, no apparent local or systemic toxicity was observed. In summary, LCF‐CSBN represents a previously unexplored OA treatment strategy that dually reprogrammed sphingolipid and AA metabolism in M1 macrophages by targeting the Golgi apparatus.

## 5 Experimental Section

### Patient Samples

Human OA synovial tissues were obtained from patients who underwent knee arthroplasty, and normal synovial tissues were collected from young patients who underwent amputation surgery. After being fixed, dehydrated, embedded, and sectioned, the specimens were processed for H&E staining, IF staining, and TEM examination. This study protocol was approved by the Ethics Committee of Xiangya Hospital, Central South University (No.202110186), and complete written consent was obtained before the operative procedure.

### Cell Line

RAW 264.7 cells were purchased from the Chinese Academy of Sciences Cell Bank for Type Culture Collection (Shanghai, China). RAW 264.7 cells were cultured at 37 °C in DMEM medium with 10% FBS and 1% 100 U mL^−1^ of penicillin–streptomycin. M1‐polarized macrophages and M2‐polarized macrophages were obtained by treating RAW 264.7 cells with 50 ng mL^−1^ of LPS or 20 ng mL^−1^ of IL‐4 for 24 h. RAW 264.7 without stimulation was represented as M0 macrophages.

### Animals

All animal trials in this study were carried out on male SD rats (8–12 weeks old) purchased from Slake Jingda Experimental Animal Co., Ltd (Changsha, China). Animals were housed in a specific pathogen‐free environment at a standard temperature of 22 ± 2 °C and relative humidity of 55% (45%–70%) in a 12:12 h light/dark cycle with free food and water intake. All animal studies were conducted according to the requirements of the national act regarding the use of experimental animals (China) and complied with the guidelines evaluated and approved by the Animal Ethics Committee of Xiangya Hospital, Central South University (No.2022111128).

### Synthesis and Characterization of CS‐BR

CS (100 mg, 0.0015 mmol; Sigma–Aldrich, St. Louis, USA), EDCI (11.5 mg, 0.06 mmol; Aladdin, Shanghai, China), 4‐Dimethylaminopyridine (DMAP, 7.33 mg, 0.06 mmol; Aladdin, Shanghai, China) and EDA (3.91 mg, 0.05 mmol; Aladdin, Shanghai, China) were dissolved in N, N‐dimethylformamide and performed for 24 h. The reaction mixtures were dialyzed against ultrapure water, CS‐EDA was obtained by lyophilized. Subsequently, BR (23.39 mg, 0.04 mmol; Tokyo Chemical Industry Co., Ltd., Tokyo, Japan), EDCI (11.5 mg, 0.06 mmol) and DMAP (7.33 mg, 0.06 mmol) were dissolved in dimethyl sulfoxide (DMSO; Macklin, Shanghai, China). After stirring at 30 °C for 30 min, CS‐EDA dissolved in 3:1 (v/v) DMSO/H_2_O solution (3 mL) was added to the mixture, and the reaction was allowed to proceed with stirring for 48 h at 30 °C under a nitrogen gas. The mixtures were dialyzed using ultrapure water to remove the organic solvent, and the dialysate was centrifuged (10 000 × *g* 10 min 4 °C) to separate the unreacted BR, the resulting precipitate was discarded, and the supernatant was collected. After lyophilization, the material was characterized by ^1^H NMR, FTIR and gel permeation chromatography (GPC) instrument.

The PEGylated BR (PEG‐BR) block was synthesized via the introduction of poly (ethylene glycol) (PEG_2000_‐NH_2_) molecules to BR, as described in previous study.^[^ ^86^ ^]^ Briefly, BR (29.23 mg, 0.05 mmol) and EDCI (11.5 mg, 0.06 mmol) were dissolved in DMSO (3 mL). After stirring for 30 min at room temperature, mPEG_2000_‐NH_2_ (50 mg, 0.025 mmol; Ponsure, Shanghai, China) and trimethylamine (45 µL) were added, this was followed by mixing for 4 h under a nitrogen gas. After adding chloroform (20 mL), the organic solvents were washed with 0.1 M HCl (60 mL) and 0.1 M NaHCO_3_ (60 mL). Subsequently, PEG‐BR was obtained by drying and evaporating the organic layer under a vacuum.

### Preparation and Characterization of LCF‐CSBN

A stock solution of LCF (Selleck, Houston, USA) dissolved in DMSO was added dropwise into the CS‐BR solution or PEG‐BR solution, followed by stirring for 10 min and ultra‐sonicating for 5 min. The LCF‐CSBN or LCF‐PEGN were obtained by ultra‐filtration to remove the unencapsulated drugs and organic solvents.^[^ ^87^ ^]^ Subsequently, the hydrodynamic diameter and zeta potential of LCF‐CSBN were determined by DLS (Zetasizer LAB, Malvern Instruments, Malvern, UK). TEM (JEM‐2100Plus, Tokyo, Japan) was used to visualize the morphology of LCF‐CSBN. The CMC value of LCF‐CSBN was determined by the light scattering method described previously.^[^ ^86^ ^]^ Distinct nanomedicine formulations were formed by using different amounts of CS‐BR in PBS, and their sizes were then analyzed by DLS. Additionally, the particle size and PDI were measured daily using the Zetasizer LAB instrument for the stability assay. ROS‐mediated decomposition of LCF‐CSBN was assessed as follows, LCF‐CSBN was treated with PBS, H_2_O_2_ (5 mM; Aladdin, Shanghai, China), AAPH (100 mM; Aladdin, Shanghai, China), and NaOCl (1 mM; Aladdin, Shanghai, China) for 1 h, respectively. The particle size was measured by DLS, and the reaction was also monitored by determining the absorbance at 450 nm using a microplate reader (TECAN, Mannedorf, Switzerland). Finally, the TEM images were captured after incubation in the presence or absence of AAPH (100 mM) for 1 h. The BR portion in LCF‐CSBN after a 7‐day incubation with synovial fluid from OA patients at 37 °C was measured using a microplate reader (TECAN, Mannedorf, Switzerland) by recording the absorbance at 450 nm. The ROS‐responsive release behavior of LCF‐CSBN was evaluated using the dialysis method, and the released LCF was analyzed by high‐performance liquid chromatography (HPLC, Agilent, CA, USA).

### Cell Internalization and Endocytosis Pathways

LPS‐activated RAW 264.7 cells were seeded and cultured for 24 h. Then the culture supernatant was replaced by DiD‐PEGBN or DiD‐CSBN in serum‐free medium, and the cells were incubated for another 4 h. Next, cells were collected, centrifuged, and suspended in PBS. A flow cytometer (BD, Franklin Lakes, USA) was used to detect the fluorescence intensity of DiD quantitatively. The cellular uptake study of DiD‐CSBN in M0, M1, M2 macrophages, and activated fibroblasts by flow cytometer was conducted as mentioned above. Meanwhile, to perform a qualitative analysis of cellular uptake, LPS‐stimulated RAW 264.7 cells were seeded in glass‐bottomed dishes, treated as described above, washed with cold PBS, stained with DAPI, and observed on a laser scanning confocal microscope (LSCM, Zeiss 900, Oberkochen, Germany). Subsequently, the endocytosis pathways of LCF‐CSBN were explored by pretreating LPS‐activated RAW 264.7 cells with the following inhibitors (chlorpromazine, amiloride, methyl‐beta‐cyclodextrin, CS; Aladdin, Shanghai, China; or CD44 antibody; MCE, USA) for 1 h. Then, DiD‐CSBN was added into the culture medium, and the cells were incubated at 4 or 37 °C for another 4 h before flow cytometry analysis.

### Subcellular Localization of LCF‐CSBN

LPS‐activated RAW 264.7 cells were seeded in glass‐bottomed dishes and treated with DiD‐PEGBN or DiD‐CSBN for 4 h. Next, cells were rinsed with cold PBS and stained with 5 µM BODIPY TR CER complexed with BSA (Thermo Fisher Scientific, Waltham, USA), 100 nM MitoTracker Green (Beyotime, Shanghai, China), or 2 mM ER Tracker Green (Thermo Fisher Scientific, Waltham, USA) for localization assays of the Golgi apparatus, mitochondria, and the endoplasmic reticulum, respectively. Additionally, LPS‐activated RAW 264.7 cells were incubated with DiD‐PEGBN or DiD‐CSBN for 1 or 4 h, washed and stained with 150 nM Lyso‐Tracker Green (Thermo Fisher Scientific, Waltham, USA) for 30 min. Finally, the subcellular localization of the nanomedicines was visualized using LSCM. The Pearson's colocalization coefficient was processed and calculated using Image Pro software.

### Intracellular ROS Scavenging

DCFH‐DA (MCE, USA) was used as a tracker for intracellular ROS measurements.^[^ ^34^ ^]^ RAW 264.7 cells were pretreated with LPS for 24 h and then immediately incubated with PBS, LCF, LCF‐PEGBN, CSBN, or LCF‐CSBN (2.7 µM LCF) for 3 h. Next, the cells were stained with 10 µM DCFH‐DA at 37 °C for 30 min, and stained with hoechst for 10 min before being photographed using a fluorescent microscopy (Leica, Wetzlar, Germany).

### In Vitro Biological Activity

LPS‐activated RAW 264.7 cells were seeded in glass‐bottomed dishes and incubated with PBS, LCF, LCF‐PEGBN, CSBN, or LCF‐CSBN (2.7 µM LCF) for 24 h. Afterward, the expression levels of GOLPH3 and GM130 were investigated by IF staining. Moreover, the morphologies of the Golgi apparatus in the treated cells were visualized by TEM to evaluate the structural changes. In brief, the treated cells were fixed, permeabilized, and stained with an anti‐GOLPH3 primary antibody (1:500, 67777‐1‐Ig, Proteintech, China) or anti‐GM130 primary antibody (1:100, ab52649, Abcam, UK), followed by the corresponding secondary antibodies.

The primary macrophages were derived from BMDMs isolated from the femurs of C57BL/6 mice aged 6–8 weeks.^[^ ^88^ ^]^ For pro‐inflammatory macrophage activation, BMDMs were treated with LPS (50 ng mL^−1^) for 24 h. Subsequently, the cells underwent three PBS washes and were treated with corresponding agents in fresh medium for an additional 24 h. The collected cells were subjected to analysis with polarization markers using flow cytometry. Cells were harvested and suspended in 1% bovine serum albumin‐PBS buffer at a concentration of 1 × 10^6^ cells mL^−1^. Next, 0.1 mL of the cell suspension was incubated with F4/80 (eBioscience, USA), CD86 (eBiosciences, USA), and CD206 (BioLegend, USA) conjugated antibodies in the dark at 4 °C for 30 min. After being washed with PBS three times, the labeled cells were resuspended in 0.2 mL of PBS and analyzed using FlowJo software.

LPS‐activated RAW 264.7 cells were seeded in 12‐well plates and treated with PBS, LCF, LCF‐PEGBN, CSBN, or LCF‐CSBN (2.7 µM LCF) for 24 h. Cell supernatants were collected to measure the content of PGE2, LTB4, TNF‐α, IL‐1 β, IL‐6, iNOS, and IL‐10 using ELISA kits (Ruixin, Shanghai, China) according to the manufacturer's instructions. To detect changes in cell subtypes after exposure to different treatment conditions, cells were stained with an anti‐iNOS antibody (1:800, ab210823, Abcam, UK) and anti‐CD206 antibody (1:1000, ab64693, Abcam, UK), followed by the corresponding secondary antibodies. Cells were lysed and total RNA was extracted using the Trizol Reagent (TaKaRa, Osaka, Japan). The cDNA was amplified using the TaKaRa reverse transcription reagents and qRT‐PCR analysis was performed using All‐in‐One qPCR Mix Kit (GeneCopoeia, China) on ABI Quant Studio 3 (Applied Biosystems, Waltham, MA). All primer sequences of target genes are shown in Table S2. And the data were presented as fold changes in comparison with endogenous controls.

To verify the drug‐repolarization mechanism, LPS‐activated RAW 264.7 cells were seeded in glass‐bottomed dishes and treated with PBS or LCF for 24 h, followed by treatment with 4‐hydroxy‐2‐nonenal (HNE) or linoleic acid (LA) for the rescue experiment. The control for the COX‐2 and 5‐LOX inhibition experiment was conducted as follows, LPS‐activated RAW 264.7 cells were treated with PBS, LCF, celecoxib (CXB), Zileuton, or S‐2474 for 24 h. Subsequently, the cells were then stained with anti‐iNOS antibody and anti‐CD206 antibody, followed by incubation with the corresponding secondary antibodies. Moreover, LPS‐activated RAW 264.7 cells were treated with PBS or LCF for 24 h, and the cells were incubated with anti‐FGFR1 or anti‐p‐FGFR1, which were then stained with Alexa Fluor 488.

### RNA Sequencing

LPS‐activated RAW 264.7 cells were treated with PBS, LCF, or LCF‐CSBN (equivalent of 2.7 µM LCF) for 24 h. RNA sequencing was performed based on previously published methods.^[^ ^89^ ^]^ Briefly, total RNA was extracted using the TRIzol reagent (Invitrogen, CA, USA) according to the manufacturer's protocol. Then the sequencing library was constructed using the NEBNext UltraTM RNA library Prep Kit for Illumina (NEB, USA) according to the manufacturer's instructions. Differential expression analysis between groups was performed using the DESeq2 R package. DEGs were defined as having a fold change ≥2 and p‐value ≤0.05. Heatmaps were generated using the heatmap package. Gene ontology (GO) enrichment analysis, KEGG pathway analysis, and GSEA were performed using clusterProfiler R package. For each group, 3 duplicates were collected for RNA‐Seq analysis.

### Lipidomics

LPS‐activated RAW 264.7 cells were treated with PBS, LCF, or LCF‐CSBN (equivalent of 2.7 µM LCF) for 24 h. The treated cells were collected and homogenized on ice, then the obtained lipids were extracted and dried under nitrogen stream. Subsequently, the lipids were reconstituted in isopropanol/methanol (1:1), then analyzed using high‐performance liquid chromatography electrospray ionization mass spectrometry (HPLC‐ESI‐MS/MS), performed on prelude SPLC + TSQ Quantiva LC‐MS/MS system.

### IA Retention Assay

Three days after IA injection of MIA (2.0 mg per knee; Sigma–Aldrich, USA), the right hind limbs of rats were shaved and received IA injection of DiD solution, DiD‐PEGBN, or DiD‐CSBN. Serially acquired fluorescence images of each joint were captured by an IVIS (Perkin Elmer, Waltham, USA) for 28 days. The radiant efficiency of the knee joints within a fixed anatomical region of interest (ROI) was determined using Living Image software, and a quantitative analysis of the area under the curve (AUC) based on the radiant efficiency of ROI was performed.

### IF Staining

To study the fluorescence distribution of DiD‐labeled nanomedicines, MIA rats were received IA injection with DiD solution, DiD‐PEGBN, DiD‐CSBN, or DiD‐CSBN pretreated with CD44 antibody. The right knee joints were collected and prepared sections 3 days after injection. After removing the embedding agent with PBS, sections were stained with anti‐CD68, anti‐iNOS, or BODIPY TR CER complexed to BSA at 4 °C overnight. The fluorescence microscope (Leica, Wetzlar, Germany) was used to observed the distributions of fluorescence in synovium of MIA rats.

For the phenotypic reprogramming ability assay and the protein expression study, MIA rats and ACLT+pMMx rats were received the indicated administration with different formulations. Knee joints were collected and prepared sections. Then the sections were stained with anti‐CD68, anti‐iNOS, anti‐CD206, anti‐GOLPH3, anti‐GM130, anti‐FGFR1, or anti‐p‐FGFR1 at 4 °C overnight, which were then captured by fluorescence microscope (Leica, Wetzlar, Germany).

### ROS Scavenging Ability of LCF‐CSBN In Vivo

Three days post MIA injection, rats were received a single IA injection of saline, LCF, CSBN, or LCF‐CSBN. A single injection of L‐012 solution at dose of 75 mg kg^−1^ was injected 28 days after MIA rats receiving different treatments. Bioluminescent images and relative amount of ROS in the OA knee joints were obtained via IVIS (Perkin Elmer, Waltham, USA).

### MIA Induced OA Rat Model

Thirty‐five 8‐week‐old SD rats were randomly divided into five groups. Twenty‐eight of the animals were subjected to MIA IA injection. Briefly, MIA (2.0 mg per knee; Sigma‐Aldrich, USA) was intra‐articularly injected into right knee of SD rat to induce OA. Rats without MIA IA injection or subsequent treatment served as controls. Three days after IA injection of MIA, a total volume of 40 µL of saline, LCF, CSBN, or LCF‐CSBN (equivalent of 40 µg of LCF) was intra‐articularly injected into the right knee of each OA rats.

### ACLT+pMMx Induced OA Rat Model

Thirty‐five 12‐week‐old SD rats were randomly divided into five groups. Among these, twenty‐eight animals were subjected to ACLT+pMMx surgery, as previously described.^[^ ^64^ ^]^ Briefly, rats were anesthetized; the right knee joint was then shaved, cleaned, and disinfected with 75% ethyl alcohol. A medial parapatellar approach of the right knee joint skin and soft tissue was used, and the patella was dislocated laterally to fully expose the joint cavity. The knee was placed in full flexion followed by transection of the anterior medial meniscotibial ligament; the anterior part of the medial meniscus was then removed using a surgical scissor. After that, the anterior cruciate ligament (ACL) was carefully transected using a micro‐surgical scalpel. Subsequently, an anterior drawer test was performed to confirm the total transection of the ACL. Ultimately, the joint capsule and its surrounding skin were sutured entirely. Rats in sham group were anesthetized and underwent identical skin and soft tissue incisions, patellar dislocation, and knee joint exposure, excluding damage to the medial meniscus and ACL. Four weeks after the establishment of surgically induced OA model, the OA rats received IA injections, following the same protocol as the rats in the MIA induced OA model, with injections administered on days 28 and 56 after surgery.

### Pain‐Related Behavior Assessment

The mechanical allodynia test and spontaneous locomotor activity were conducted to assess pain‐related behaviors in animals. The mechanical allodynia test was performed weekly using an electronic von Frey aesthesiometer (IITC, Woodland Hills, CA, USA) as previously described.^[^ ^90^ ^]^ Briefly, the rats were placed in transparent plastic cubicles on a mesh floored table and were allowed to acclimate for 15 min before the test. The plantar surface of the hind paw was stimulated with ascending force intensities of von Frey filaments. Positive responses, including brisk paw withdrawal, licking, or shaking, were meticulously recorded; the instrument automatically tallied the number of positive responses for each stimulus. For each rat, this test was performed three times with a time interval of at least 5 min between two adjacent stimuli. The final threshold value was obtained by calculating the mean value of three readings. Spontaneous locomotor activity was assessed using the PhenoRack system (ViewPoint, Lyon, France) as previously described.^[^ ^91^ ^]^ Rats were individually housed in large cages (50 cm long × 50 cm wide ×45 cm high) and given free access to food and water, underwent a 24 h habituation period. Infrared cameras recorded their continuous activities during this period, and the Videotrack software (Viewpoint, Lyon, France) automatically generated locomotor activity parameters, such as the number and duration of climbing attempts. All assessments were performed by the same investigator blinded to the study groups and identification of animals.

### µCT Analysis

Knee joint samples were collected and fixed for 72 h with 4% paraformaldehyde (PFA) and were then scanned using µCT (Skyscan 1176, Skyscan, Aartselaar, Belgium) with a high resolution of 9 µm at 50 kV/200 µA. The NRecon v1.6 and CTAn v1.13.8.1 software was used for data reconstruction. Scanned images from each group were subjected to the same thresholds to allow for 3D structural remodeling. Osteophyte formation was assessed using 3D analysis in CTAn.

### Histological Analysis, IHC Staining, and TUNEL Assay

Rats were sacrificed after the final behavior assessment, and right knee joints were collected. Samples were fixed in 4% PFA for 72 h, decalcified in EDTA for 6 weeks, and then embedded in paraffin. Serial sagittal sections of knee joints were cut every 5 µm from the medial compartments. The sections were stained with H&E and Safranine O‐Fast green stain for histological analysis. Respectively, synovitis scoring and OARSI scoring were performed by two independent experts to evaluate synovial inflammation and articular cartilage destruction. For IHC staining, after appropriate antigen retrieval, slides were incubated with primary antibodies (i.e., anti‐TNF‐α, anti‐IL‐1β, anti‐IL‐6, anti‐CGRP, anti‐NGF, anti‐COL II, anti‐ACAN, anti‐MMP13, anti‐ADAMTS5, anti‐N‐SMase, anti‐COX‐2, anti‐LOX‐5, anti‐CD68 and anti‐CD206) at 4 °C overnight, followed by binding with biotinylated secondary antibodies and 3,3′‐ diaminobenzidine (DAB) color developer. The TUNEL assay was carried out according to the manufacturer's instructions (Beyotime, China).

### Safety Evaluation

To investigate the in vivo safety of LCF‐CSBN, MIA rats and ACLT+pMMx rats were treated as detailed in the therapeutic efficacy part of the study. Treatment was administered by IA injection once every 4 weeks, and rats were sacrificed 4 weeks after the last treatment. Blood samples and major organs (i.e., heart, liver, lung, kidney, and spleen) were collected for serum enzyme analysis and H&E staining, respectively. To assess the in vitro safety of LCF‐CSBN against M1 macrophages, cells were treated with LCF or LCF‐CSBN at concentrations of LCF equivalents ranging from 0.0098−2.5 µg mL^−1^ for 24 h. Subsequently, CCK‐8 reagents (NCM Biotech, China) were added to the wells, followed by measuring the optical density (OD) at 450 nm using the multifunction microplate reader (BioTek Epoch, USA) according to the manufacturer's instructions.

### Statistical Analysis

All data were presented as the mean ± standard deviation. Statistical analyses were performed using a student's two‐sided t test (for comparisons of two groups), or one‐way (for comparisons between multiple groups of one variable) or two‐way analysis of variance (for comparisons between multiple groups of two different variables) (ANOVA) with Tukey's post hoc test. All statistical analyses were performed using Graph Pad Prism version 8.0 software. Differences were considered statistically significant at * *p* < 0.05, ** *p* < 0.01, *** *p* < 0.001, **** *p* < 0.0001.

## Conflict of Interest

The authors declare no conflict of interest.

## Supporting information



Supporting Information

## Data Availability

The data that support the findings of this study are available from the corresponding author upon reasonable request.

## References

[1] K. Ganeshan , A. Chawla , Annu. Rev. Immunol. 2014, 32, 609. 24655299 10.1146/annurev-immunol-032713-120236 PMC5800786

[2] A. Shapouri‐Moghaddam , S. Mohammadian , H. Vazini , M. Taghadosi , S. A. Esmaeili , F. Mardani , B. Seifi , A. Mohammadi , J. T. Afshari , A. Sahebkar , J. Cell. Physiol. 2018, 233, 6425. 10.1002/jcp.26429 PMC13482560 29319160

[3] H. Zhang , C. Lin , C. Zeng , Z. Wang , H. Wang , J. Lu , X. Liu , Y. Shao , C. Zhao , J. Pan , S. Xu , Y. Zhang , D. Xie , D. Cai , X. Bai , Ann. Rheum. Dis. 2018, 77, 1524. 29991473 10.1136/annrheumdis-2018-213450

[4] L. Kuang , J. Wu , N. Su , H. Qi , H. Chen , S. Zhou , Y. Xiong , Du X. , Q. Tan , J. Yang , M. Jin , F. Luo , J. Ouyang , B. Zhang , Z. Wang , W. Jiang , L. Chen , S. Chen , Z. Wang , P. Liu , L. Yin , F. Guo , C. Deng , D. Chen , C. Liu , Y. Xie , Z. Ni , L. Chen , Ann. Rheum. Dis. 2020, 79, 112. 31662319 10.1136/annrheumdis-2019-215696

[5] W. H. Robinson , C. M. Lepus , Q. Wang , H. Raghu , R. Mao , T. M. Lindstrom , J. Sokolove , Nat. Rev. Rheumatol. 2016, 12, 580. 27539668 10.1038/nrrheum.2016.136 PMC5500215

[6] S. Yoshida , K. Nishitani , H. Yoshitomi , S. Kuriyama , S. Nakamura , T. Fujii , M. Saito , Y. Kobori , A. Murakami , K. Murata , H. Ito , H. Ueno , S. Matsuda , Arthritis Rheumatol. 2023, 75, 950. 36530127 10.1002/art.42424

[7] S. K. Wculek , G. Dunphy , I. Heras‐Murillo , A. Mastrangelo , D. Sancho , Cell Mol. Immunol. 2022, 19, 384. 34876704 10.1038/s41423-021-00791-9 PMC8891297

[8] A. Hooftman , C. G. Peace , D. G. Ryan , E. A. Day , M. Yang , A. F. Mcgettrick , M. Yin , E. N. Montano , L. Huo , J. E. Toller‐Kawahisa , V. Zecchini , T. Ryan , A. Bolado‐Carrancio , A. M. Casey , H. A. Prag , A. Costa , S. G. De Los , M. Ishimori , D. J. Wallace , S. Venuturupalli , E. Nikitopoulou , N. Frizzell , C. Johansson , A. Von Kriegsheim , M. P. Murphy , C. Jefferies , C. Frezza , L. O'Neill , Nature 2023, 615, 490. 36890227 10.1038/s41586-019-0000-0 PMC10411300

[9] M. Li , Y. Yang , L. Xiong , P. Jiang , J. Wang , C. Li , J. Hematol. Oncol. 2023, 16, 80. 37491279 10.1186/s13045-023-01478-6 PMC10367370

[10] P. Romani , I. Brian , G. Santinon , A. Pocaterra , M. Audano , S. Pedretti , S. Mathieu , M. Forcato , S. Bicciato , J. B. Manneville , N. Mitro , S. Dupont , Nat. Cell Biol. 2019, 21, 338. 30718857 10.1038/s41556-018-0270-5

[11] M. Thery , M. Pende , Nat. Cell Biol. 2019, 21, 301. 30718858 10.1038/s41556-019-0289-2

[12] J. I. Sbodio , S. H. Snyder , B. D. Paul , Proc. Natl. Acad. Sci. USA 2018, 115, 780. 29317536 10.1073/pnas.1717877115 PMC5789946

[13] Z. Jiang , Z. Hu , L. Zeng , W. Lu , H. Zhang , T. Li , H. Xiao , Free Radic. Biol. Med. 2011, 50, 907. 21241794 10.1016/j.freeradbiomed.2011.01.011

[14] C. E. Chalfant , S. Spiegel , J. Cell Sci. 2005, 118, 4605. 16219683 10.1242/jcs.02637

[15] K. Hanada , K. Kumagai , S. Yasuda , Y. Miura , M. Kawano , M. Fukasawa , M. Nishijima , Nature 2003, 426, 803. 14685229 10.1038/nature02188

[16] S. W. Hicks , C. E. Machamer , Biochim. Biophys. Acta 2005, 1744, 406. 15979510 10.1016/j.bbamcr.2005.03.002

[17] H. Sun , S. Sun , G. Chen , H. Xie , S. Yu , X. Lin , J. Qian , C. Mao , H. Peng , H. Chen , X. Chen , Y. Li , C. Liu , J. Shi , B. Zhu , L. Guo , Q. Li , P. Huang , Y. Wei , X. Huang , M. Liu , Z. Cui , Q. Zhang , J. Zhou , C. Li , K. Wang , Cell Death Dis. 2021, 12, 324. 33771984 10.1038/s41419-021-03616-9 PMC7998020

[18] B. T. Bikman , S. A. Summers , J. Clin. Invest. 2011, 121, 4222. 22045572 10.1172/JCI57144 PMC3204836

[19] H. Wen , D. Gris , Y. Lei , S. Jha , L. Zhang , M. T. Huang , W. J. Brickey , J. P. Ting , Nat. Immunol. 2011, 12, 408. 21478880 10.1038/ni.2022 PMC4090391

[20] A. Castoldi , L. B. Monteiro , B. N. van Teijlingen , D. E. Sanin , N. Rana , M. Corrado , A. M. Cameron , F. Hassler , M. Matsushita , G. Caputa , G. R. Klein , J. Buscher , J. Edwards‐Hicks , E. L. Pearce , E. J. Pearce , Nat. Commun. 2020, 11, 4107. 32796836 10.1038/s41467-020-17881-3 PMC7427976

[21] S. Wang , B. Li , V. Solomon , A. Fonteh , S. I. Rapoport , D. A. Bennett , Z. Arvanitakis , H. C. Chui , P. M. Sullivan , H. N. Yassine , Mol. Neurodegener. 2022, 17, 42. 35705959 10.1186/s13024-022-00549-5 PMC9202185

[22] T. Xiong , K. Yang , T. Zhao , H. Zhao , X. Gao , Z. You , C. Fan , X. Kang , W. Yang , Y. Zhuang , Y. Chen , J. Dai , Adv. Sci. (Weinh) 2023, 10, e2205997. 36646515 10.1002/advs.202205997 PMC9982579

[23] X. Li , X. Wang , Q. Liu , J. Yan , D. Pan , L. Wang , Y. Xu , F. Wang , Y. Liu , X. Li , M. Yang , Adv. Healthcare Mater. 2021, 10, e2100883. 10.1002/adhm.202100883 34137218

[24] S. T. Sanjay , W. Zhou , M. Dou , H. Tavakoli , L. Ma , F. Xu , X. Li , Adv. Drug Deliv. Rev. 2018, 128, 3. 28919029 10.1016/j.addr.2017.09.013 PMC5854505

[25] A. J. Gormley , C. D. Spicer , R. Chandrawati , Adv. Drug Deliv. Rev. 2021, 174, 628. 34022270 10.1016/j.addr.2021.05.022

[26] L. H. Tostanoski , C. M. Jewell , Adv. Drug Deliv. Rev. 2017, 114, 60. 28392305 10.1016/j.addr.2017.03.005 PMC6262758

[27] D. L. R. J. Rios , A. Tirella , A. Gennari , I. J. Stratford , N. Tirelli , Adv. Healthcare Mater. 2017, 6, 4. 10.1002/adhm.201601012 27990775

[28] J. Luo , P. Zhang , T. Zhao , M. Jia , P. Yin , W. Li , Z. R. Zhang , Y. Fu , T. Gong , ACS Nano 2019, 13, 3910. 30938986 10.1021/acsnano.8b06924

[29] H. Li , P. Zhang , J. Luo , D. Hu , Y. Huang , Z. R. Zhang , Y. Fu , T. Gong , ACS Nano 2019, 13, 9386. 31375027 10.1021/acsnano.9b04166

[30] H. Li , C. Deng , Y. Tan , J. Dong , Y. Zhao , X. Wang , X. Yang , J. Luo , H. Gao , Y. Huang , Z. R. Zhang , T. Gong , Acta Biomater. 2022, 146, 357. 35577045 10.1016/j.actbio.2022.05.014

[31] T. W. Sedlak , M. Saleh , D. S. Higginson , B. D. Paul , K. R. Juluri , S. H. Snyder , Proc. Natl. Acad. Sci. USA 2009, 106, 5171. 19286972 10.1073/pnas.0813132106 PMC2664041

[32] K. Ishikawa , X. Xie , Y. Osaki , A. Miyawaki , K. Numata , Y. Kodama , Sci. Adv. 2023, 9, eadh4787. 37285441 10.1126/sciadv.adh4787 PMC10246902

[33] Y. Lee , K. Sugihara , M. R. Gillilland , S. Jon , N. Kamada , J. J. Moon , Nat. Mater. 2020, 19, 118. 31427744 10.1038/s41563-019-0462-9 PMC6923573

[34] H. Huang , S. Zheng , J. Wu , X. Liang , S. Li , P. Mao , Z. He , Y. Chen , L. Sun , X. Zhao , A. Cai , L. Wang , H. Sheng , Q. Yao , R. Chen , Y. Z. Zhao , L. Kou , Adv. Sci. (Weinh) 2024, 11, e2400713. 38593402 10.1002/advs.202400713 PMC11165524

[35] J. J. Dinicolantonio , M. F. Mccarty , J. H. O'Keefe , Open Heart 2018, 5, e000914. 30364545 10.1136/openhrt-2018-000914 PMC6196942

[36] S. X. Chen , F. Xue , Y. Kuang , S. Chen , D. Sheng , H. Chen , Biomaterials 2021, 269, 120533. 33228991 10.1016/j.biomaterials.2020.120533

[37] C. Ospelt , M. Kurowska‐Stolarska , M. Neidhart , B. A. Michel , R. E. Gay , S. Laufer , S. Gay , Ann. Rheum. Dis. 2008, 67, 524. 17666446 10.1136/ard.2007.071589

[38] S. K. Kulkarni , V. P. Singh , Curr. Rheumatol. Rep. 2008, 10, 43. 18457611 10.1007/s11926-008-0008-7

[39] T. Dusabimana , J. Je , S. P. Yun , H. J. Kim , H. Kim , S. W. Park , Cell Death Dis. 2023, 14, 458. 37479687 10.1038/s41419-023-05975-x PMC10361983

[40] X. Li , J. Yu , L. Gong , Y. Zhang , S. Dong , J. Shi , C. Li , Y. Li , Y. Zhang , H. Li , Free Radic. Biol. Med. 2021, 165, 243. 33493554 10.1016/j.freeradbiomed.2021.01.028 PMC7825924

[41] B. B. Liang , Q. Liu , B. Liu , H. G. Yao , J. He , C. F. Tang , K. Peng , X. X. Su , Y. Zheng , J. Y. Ding , J. Shen , Q. Cao , Z. W. Mao , Angew. Chem. Int. Ed. Engl. 2023, 62, e202312170. 37710398 10.1002/anie.202312170

[42] R. Huang , X. Wang , Y. Zhou , Y. Xiao , Bone Res. 2017, 5, 17019. 29263936 10.1038/boneres.2017.19 PMC5645773

[43] L. Zhang , X. Chen , P. Cai , H. Sun , S. Shen , B. Guo , Q. Jiang , Adv. Mater. 2022, 34, 2202715. 10.1002/adma.202202715 35671349

[44] M. Y. Kwon , C. Wang , J. H. Galarraga , E. Pure , L. Han , J. A. Burdick , Biomaterials 2019, 222, 119451. 31480001 10.1016/j.biomaterials.2019.119451 PMC6746338

[45] K. B. Japiassu , F. Fay , A. Marengo , Y. Louaguenouni , C. Cailleau , S. Denis , D. Chapron , N. Tsapis , T. L. Nascimento , E. M. Lima , E. Fattal , J. Control Release 2022, 352, 15. 36209941 10.1016/j.jconrel.2022.10.006

[46] M. Liu , Y. Chen , Y. Guo , H. Yuan , T. Cui , S. Yao , S. Jin , H. Fan , C. Wang , R. Xie , W. He , Z. Guo , Nat. Commun. 2022, 13, 2179. 35449133 10.1038/s41467-022-29872-7 PMC9023483

[47] Q. Yao , R. Chen , V. Ganapathy , L. Kou , J. Control Release 2020, 328, 407. 32882272 10.1016/j.jconrel.2020.08.054

[48] S. Kang , Y. Nakanishi , Y. Kioi , D. Okuzaki , T. Kimura , H. Takamatsu , S. Koyama , S. Nojima , M. Nishide , Y. Hayama , Y. Kinehara , Y. Kato , T. Nakatani , T. Shimogori , J. Takagi , T. Toyofuku , A. Kumanogoh , Nat. Immunol. 2018, 19, 561. 29777213 10.1038/s41590-018-0108-0

[49] Y. N. Zhao , Z. D. Liu , T. Yan , T. X. Xu , T. Y. Jin , Y. S. Jiang , W. Zuo , K. Y. Lee , L. J. Huang , Y. Wang , Acta Pharmacol. Sin. 2024, 45, 988. 38279043 10.1038/s41401-024-01226-7 PMC11053141

[50] L. Wang , W. Luo , S. Zhang , J. Zhang , L. He , Y. Shi , L. Gao , B. Wu , X. Nie , C. Hu , X. Han , C. He , B. Xu , G. Liang , Cardiovasc. Res. 2024, 120, 1385. 38842387 10.1093/cvr/cvae131

[51] C. K. Glass , J. L. Witztum , CellCell 2001, 104, 503. 10.1016/s0092-8674(01)00238-0 11239408

[52] X. P. Zhong , R. Guo , H. Zhou , C. Liu , C. K. Wan , Immunol. Rev. 2008, 224, 249. 18759932 10.1111/j.1600-065X.2008.00647.x PMC3342643

[53] A. Romanauska , A. Kohler , Dev. Cell 2021, 56, 2562. 34407429 10.1016/j.devcel.2021.07.018 PMC8480995

[54] A. Romanauska , E. Stankunas , M. Schuldiner , A. Kohler , Nat. Commun. 2024, 15, 10486. 39622802 10.1038/s41467-024-54811-z PMC11612446

[55] M. Ouimet , H. N. Ediriweera , U. M. Gundra , F. J. Sheedy , B. Ramkhelawon , S. B. Hutchison , K. Rinehold , C. van Solingen , M. D. Fullerton , K. Cecchini , K. J. Rayner , G. R. Steinberg , P. D. Zamore , E. A. Fisher , P. Loke , K. J. Moore , J. Clin. Invest. 2015, 125, 4334. 26517695 10.1172/JCI81676 PMC4665799

[56] B. Tu , R. Fang , Z. Zhu , G. Chen , C. Peng , R. Ning , Inflamm. Res. 2023, 72, 955. 36995411 10.1007/s00011-023-01720-4

[57] T. Yu , M. Gao , P. Yang , D. Liu , D. Wang , F. Song , X. Zhang , Y. Liu , J. Cell. Physiol. 2019, 234, 4217. 30132863 10.1002/jcp.27185

[58] F. Zhou , J. Mei , X. Han , H. Li , S. Yang , M. Wang , L. Chu , H. Qiao , T. Tang , Acta Pharm. Sin. B 2019, 9, 973. 31649847 10.1016/j.apsb.2019.01.015 PMC6804452

[59] K. C. Shin , I. Hwang , S. S. Choe , J. Park , Y. Ji , J. I. Kim , G. Y. Lee , S. H. Choi , J. Ching , J. P. Kovalik , J. B. Kim , Nat. Commun. 2017, 8, 1087. 29057873 10.1038/s41467-017-01232-w PMC5651811

[60] I. A. Jones , R. Togashi , M. L. Wilson , N. Heckmann , C. J. Vangsness , Nat. Rev. Rheumatol. 2019, 15, 77. 30498258 10.1038/s41584-018-0123-4 PMC6390843

[61] M. C. Bruno , M. C. Cristiano , C. Celia , N. D'Avanzo , A. Mancuso , D. Paolino , J. Wolfram , M. Fresta , ACS Nano 2022, 16, 19665. 36512378 10.1021/acsnano.2c06393

[62] M. Alves‐Simoes , Osteoarthr. Cartil. 2022, 30, 802.

[63] T. Hayami , M. Pickarski , Y. Zhuo , G. A. Wesolowski , G. A. Rodan , L. T. Duong , Bone 2006, 38, 234. 16185945 10.1016/j.bone.2005.08.007

[64] C. B. Hamilton , M. A. Pest , V. Pitelka , A. Ratneswaran , F. Beier , B. M. Chesworth , Osteoarthr. Cartil. 2015, 23, 1178. 10.1016/j.joca.2015.03.001 25771150

[65] Y. D. Xu , X. C. Liang , Z. P. Li , Z. S. Wu , J. Yang , S. Z. Jia , R. Peng , Z. Y. Li , X. H. Wang , F. J. Luo , J. J. Chen , W. X. Cheng , P. Zhang , Z. G. Zha , R. Zeng , H. T. Zhang , Biomaterials 2024, 306, 122483. 38330742 10.1016/j.biomaterials.2024.122483

[66] C. Xue , J. Tian , Z. Cui , Y. Liu , D. Sun , M. Xiong , N. Yi , K. Wang , X. Li , Y. Wang , H. Xu , W. Zhang , Q. Liang , Bioact. Mater. 2024, 33, 545. 38162513 10.1016/j.bioactmat.2023.10.032 PMC10755683

[67] J. I. Odegaard , R. R. Ricardo‐Gonzalez , M. H. Goforth , C. R. Morel , V. Subramanian , L. Mukundan , E. A. Red , D. Vats , F. Brombacher , A. W. Ferrante , A. Chawla , Nature 2007, 447, 1116. 17515919 10.1038/nature05894 PMC2587297

[68] Q. Zhuo , W. Yang , J. Chen , Y. Wang , Nat. Rev. Rheumatol. 2012, 8, 729. 22907293 10.1038/nrrheum.2012.135

[69] S. Mitsutake , T. Date , H. Yokota , M. Sugiura , T. Kohama , Y. Igarashi , FEBS Lett. 2012, 586, 1300. 22465662 10.1016/j.febslet.2012.03.032

[70] C. Shah , G. Yang , I. Lee , J. Bielawski , Y. A. Hannun , F. Samad , J. Biol. Chem. 2008, 283, 13538. 18359942 10.1074/jbc.M709950200 PMC2376236

[71] J. Han , E. Li , L. Chen , Y. Zhang , F. Wei , J. Liu , H. Deng , Y. Wang , Nature 2015, 524, 243. 26147081 10.1038/nature14557

[72] D. Huang , J. Gui , X. Chen , R. Yu , T. Gong , Z. Zhang , Y. Fu , ACS Appl. Mater. Interfaces 2022, 14, 51776. 36350778 10.1021/acsami.2c15881

[73] M. T. Tarrago‐Trani , B. Storrie , Adv. Drug Deliv. Rev. 2007, 59, 782. 17669543 10.1016/j.addr.2007.06.006 PMC2134838

[74] G. Wang , A. S. Norton , D. Pokharel , Y. Song , R. A. Hill , Nanomedicine 2013, 9, 366. 23041411 10.1016/j.nano.2012.09.002

[75] R. E. Lawrence , R. Zoncu , Nat. Cell Biol. 2019, 21, 133. 30602725 10.1038/s41556-018-0244-7

[76] J. Uson , S. C. Rodriguez‐Garcia , R. Castellanos‐Moreira , T. W. O'Neill , M. Doherty , M. Boesen , H. Pandit , P. I. Moller , V. Vardanyan , L. Terslev , W. U. Kampen , M. A. D'Agostino , F. Berenbaum , E. Nikiphorou , I. A. Pitsillidou , J. de la Torre‐Aboki , L. Carmona , E. Naredo , Ann. Rheum. Dis. 2021, 80, 1299. 34035002 10.1136/annrheumdis-2021-220266 PMC8458067

[77] R. Xie , H. Yao , A. S. Mao , Y. Zhu , D. Qi , Y. Jia , M. Gao , Y. Chen , L. Wang , D. A. Wang , K. Wang , S. Liu , L. Ren , C. Mao , Nat. Biomed. Eng. 2021, 5, 1189. 34608279 10.1038/s41551-021-00785-y

[78] Y. Wei , L. Luo , T. Gui , F. Yu , L. Yan , L. Yao , L. Zhong , W. Yu , B. Han , J. M. Patel , J. F. Liu , F. Beier , L. S. Levin , C. Nelson , Z. Shao , L. Han , R. L. Mauck , A. Tsourkas , J. Ahn , Z. Cheng , L. Qin , Sci. Transl. Med. 2021, 13, eabb3946. 33441426 10.1126/scitranslmed.abb3946 PMC8027922

[79] Y. Wei , L. Yan , L. Luo , T. Gui , B. Jang , A. Amirshaghaghi , T. You , A. Tsourkas , L. Qin , Z. Cheng , Sci. Adv. 2021, 7, eabe6374. 33827816 10.1126/sciadv.abe6374 PMC8026133

[80] S. Li , W. Zheng , W. Deng , Z. Li , J. Yang , H. Zhang , Z. Dai , W. Su , Z. Yan , W. Xue , X. Yun , S. Mi , J. Shen , X. Luo , L. Wang , Y. Wu , W. Huang , Adv. Sci. (Weinh) 2024, 11, e2403227. 38704731 10.1002/advs.202403227 PMC11234466

[81] F. Yuan , L. D. Quan , L. Cui , S. R. Goldring , D. Wang , Adv. Drug Deliv. Rev. 2012, 64, 1205. 22433784 10.1016/j.addr.2012.03.006 PMC3572768

[82] D. J. Hunter , S. Bierma‐Zeinstra , Lancet 2019, 393, 1745. 31034380 10.1016/S0140-6736(19)30417-9

[83] J. N. Katz , K. R. Arant , R. F. Loeser , JAMA, J. Am. Med. Assoc. JAMA, J. Am. Med. Assoc. 2021, 325, 568.

[84] A. Thomson , C. Hilkens , Front. Immunol. 2021, 12, 678757. 34211470 10.3389/fimmu.2021.678757 PMC8239355

[85] E. Sanchez‐Lopez , R. Coras , A. Torres , N. E. Lane , M. Guma , Nat. Rev. Rheumatol. 2022, 18, 258. 35165404 10.1038/s41584-022-00749-9 PMC9050956

[86] Y. Lee , H. Kim , S. Kang , J. Lee , J. Park , S. Jon , Angew. Chem. Int. Ed. Engl. 2016, 55, 7460. 27144463 10.1002/anie.201602525

[87] Y. Zhou , F. Tong , W. Gu , S. He , X. Yang , J. Li , Y. D. Gao , H. Gao , Acta Pharm. Sin. B 2022, 12, 1416. 35530138 10.1016/j.apsb.2021.12.001 PMC9072251

[88] D. Han , F. Wang , Q. Jiang , Z. Qiao , Y. Zhuang , Q. An , Y. Li , Y. Tang , C. Li , D. Shen , Adv. Sci. (Weinh) 2024, 11, e2406124. 39264272 10.1002/advs.202406124 PMC11558124

[89] Y. Zhu , L. Cao , M. Yuan , X. Chen , X. Xie , M. Li , C. Yang , X. Wang , Z. Ma , Adv. Sci. (Weinh) 2024, 11, e2404396. 39248388 10.1002/advs.202404396 PMC11538678

[90] S. R. Chaplan , F. W. Bach , J. W. Pogrel , J. M. Chung , T. L. Yaksh , J. Neurosci. Methods 1994, 53, 55. 7990513 10.1016/0165-0270(94)90144-9

[91] V. Matagne , E. Borloz , Y. Ehinger , L. Saidi , L. Villard , J. C. Roux , Neurobiol. Dis. 2021, 149, 105235. 33383186 10.1016/j.nbd.2020.105235

