# Correction to “Sequential Targeting Chondroitin Sulfate‐Bilirubin Nanomedicine Attenuates Osteoarthritis via Reprogramming Lipid Metabolism in M1 Macrophages”

**DOI:** 10.1002/advs.202507227

**Published:** 2025-05-26

Deng C, Xiao Y, Zhao X, et al. Sequential Targeting Chondroitin Sulfate‐Bilirubin Nanomedicine Attenuates Osteoarthritis via Reprogramming Lipid Metabolism in M1 Macrophages. *Advanced science*, 2025 Mar;12(9):e2411911.

In Figure 5E, the co‐immunostaining images of cell staining for the PBS, LCF, and LCF‐PEGBN groups were incorrectly presented due to improper panel preparation during ImageJ processing. We have since verified the original data and replaced the images in Figure 5E (PBS, LCF, and LCF‐PEGBN) with the correct versions, as presented below.


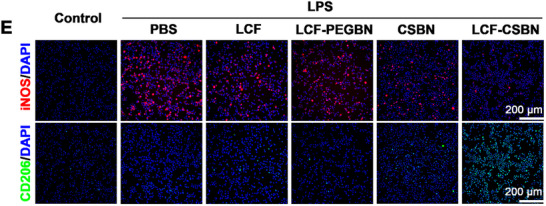


This correction does not affect the overall findings and conclusions of this paper. We apologize for this error.

