# Supplementary material for: Sequential Targeting Chondroitin Sulfate‐Bilirubin Nanomedicine Attenuates Osteoarthritis via Reprogramming Lipid Metabolism in M1 Macrophages
Source: Adv Sci (Weinh). 2025 Jan 10;12(9):2411911. doi: 10.1002/advs.202411911 (PMC11884591; doi:10.1002/advs.202411911)
Supplement: Supplementary file 1 — Supporting Information [file ADVS-12-2411911-s001.docx]

**Supporting Information**

**Sequential Targeting Chondroitin Sulfate-Bilirubin Nanomedicine Attenuates Osteoarthritis via Reprogramming Lipid Metabolism in M1 Macrophages**

*Caifeng Deng, Yongbing Xiao, Xuan Zhao, Hui Li, Yuxiao Chen, Kelong Ai, Ting Jiang, Jie Wei,* Xiaoyuan Chen,** *Guanghua Lei,* and Chao Zeng**

**
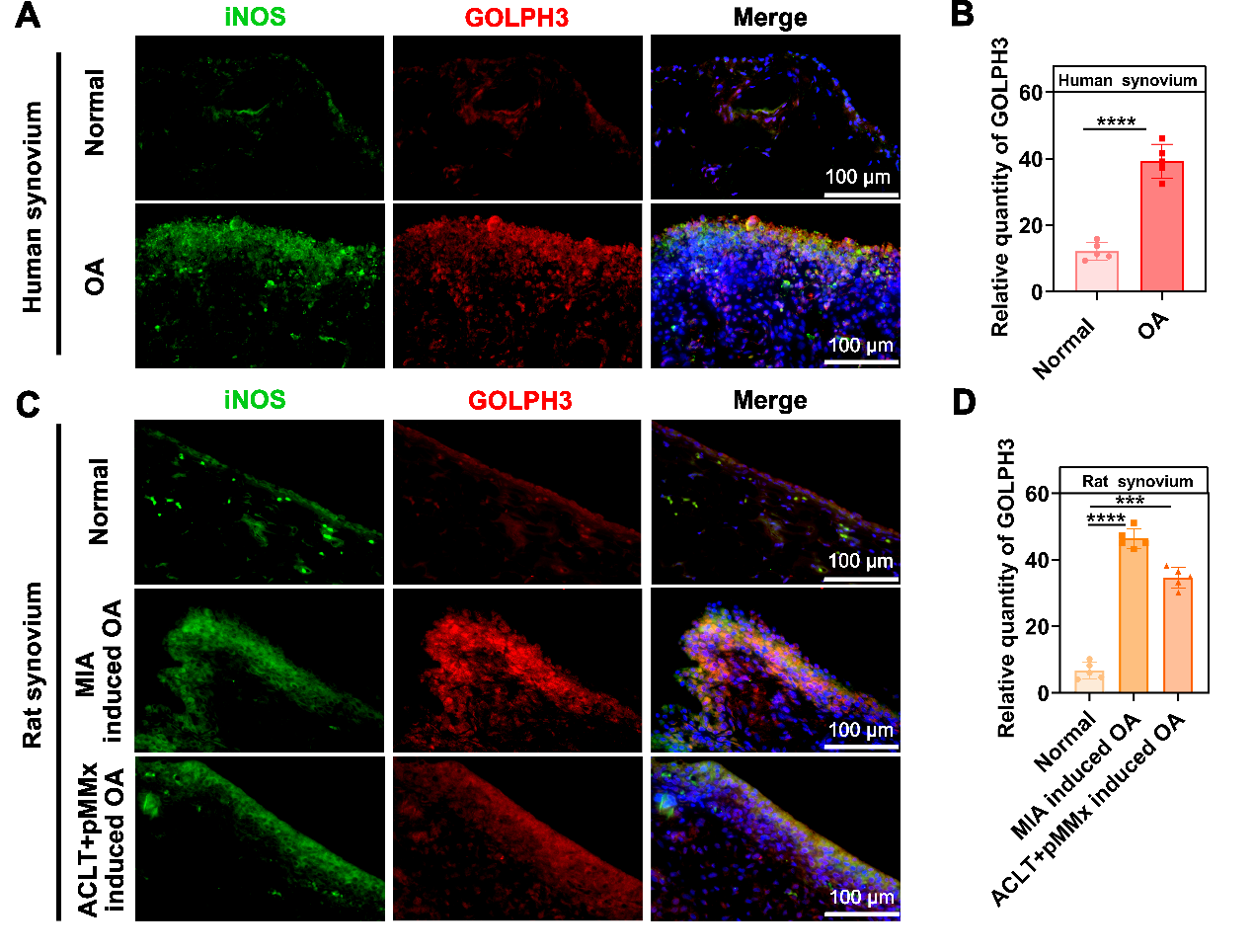
**

**Figure S1.** M1 macrophages in OA synovium express high levels of GOLPH3. A,B) Representative coimmunostaining images of GOLPH3 in synovial M1 macrophages from healthy controls and OA patients (A), and the relative quantity of GOLPH3 (B), synovial M1 macrophages were stained with iNOS antibody (*n* = 5, mean ± SD). C,D) Representative coimmunostaining images of GOLPH3 in synovial M1 macrophages from normal and OA rats (C), and the relative quantity of GOLPH3 (D), synovial M1 macrophages were stained with iNOS antibody (*n* = 5, mean ± SD). ********P* < 0.001, *********P* < 0.0001, as determined by student’s two-sided t test (B) or one-way ANOVA with Tukey’s post hoc test (D).


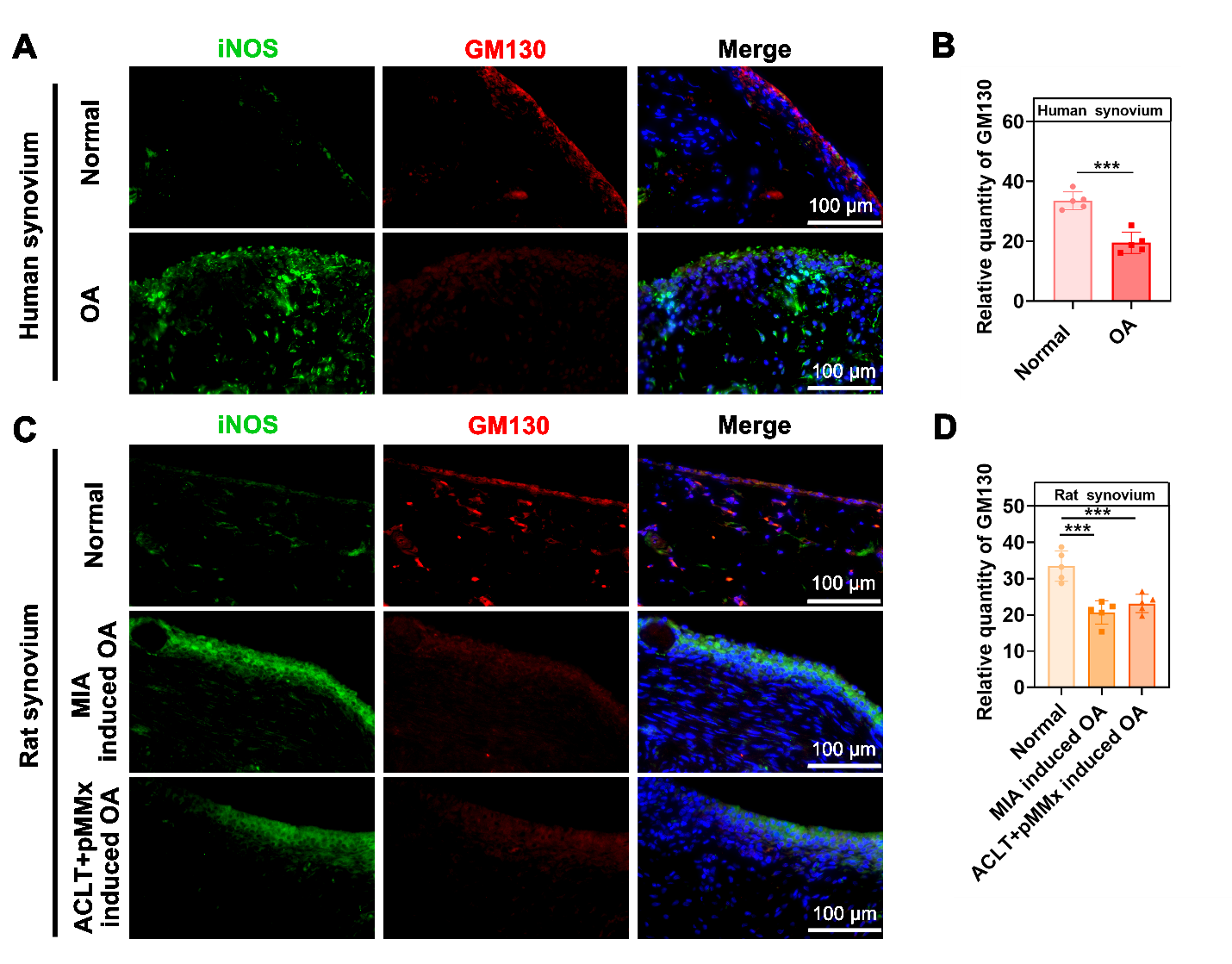


**Figure S2.** M1 macrophages in OA synovium express low levels of GM130. A,B) Representative coimmunostaining images of GM130 in synovial M1 macrophages from healthy controls and OA patients (A), and the relative quantity of GM130 (B), synovial M1 macrophages were stained with iNOS antibody (*n* = 5, mean ± SD). C,D) Representative coimmunostaining images of GM130 in synovial M1 macrophages from healthy and OA rats (C), and the relative quantity of GM130 (D), synovial M1 macrophages were stained with iNOS antibody (*n* = 5, mean ± SD). ********P* < 0.001, as determined by student’s two-sided t test (B) or one-way ANOVA with Tukey’s post hoc test (D).


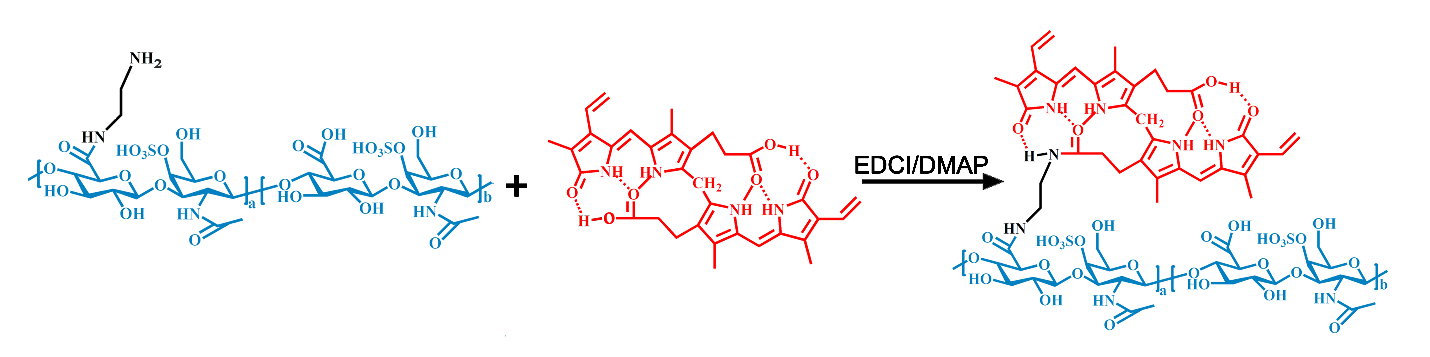


**Figure S3.** Synthetic route of chondroitin sulfate-bilirubin (CS-BR) conjugation.

**
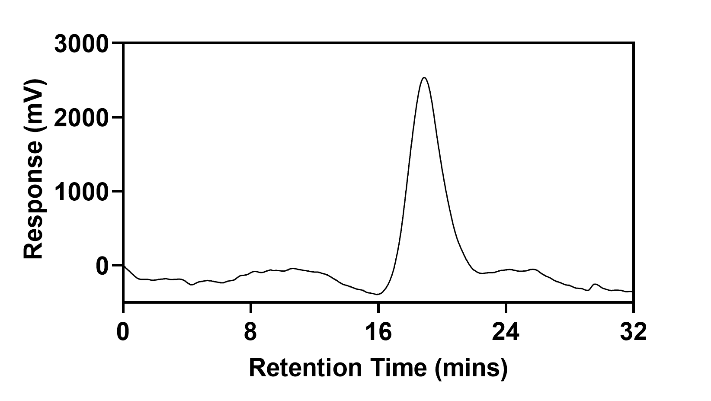
**

**Figure S4.** The gel permeation chromatography curve of CS-BR conjugation.


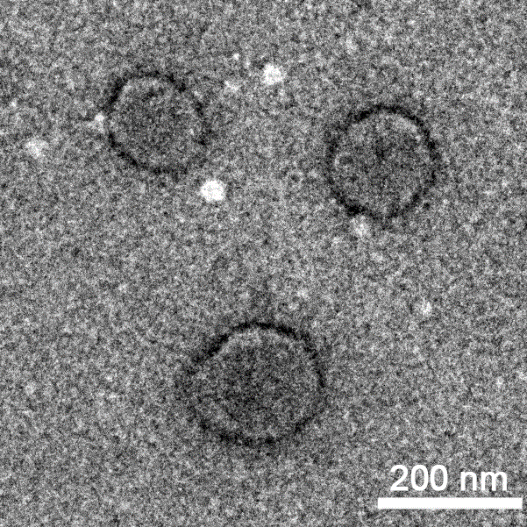


**Figure S5.** Representative TEM image of LCF-PEGBN.

**
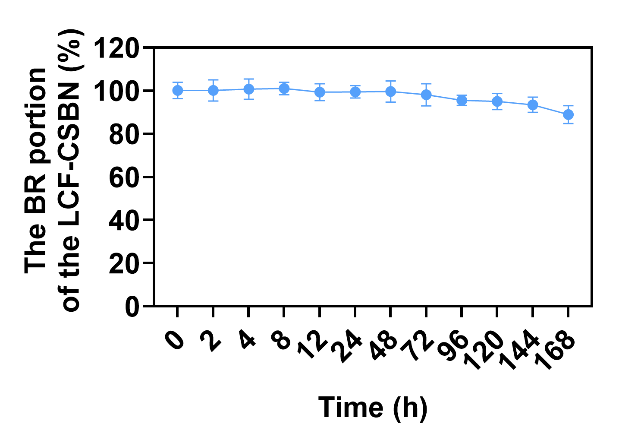
**

**Figure S6.** Changes in BR portion of LCF-CSBN incubated with synovial fluid from OA patients for 7 days at 37 °C (*n* = 3, mean ± SD).

**
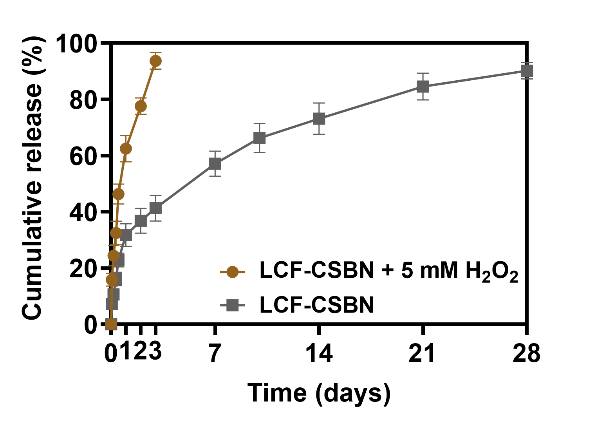
**

**Figure S7**. Release profiles of LCF-CSBN in PBS with or without 5 mM of H_2_O_2_ at 37 °C (*n* = 3, mean ± SD).

**
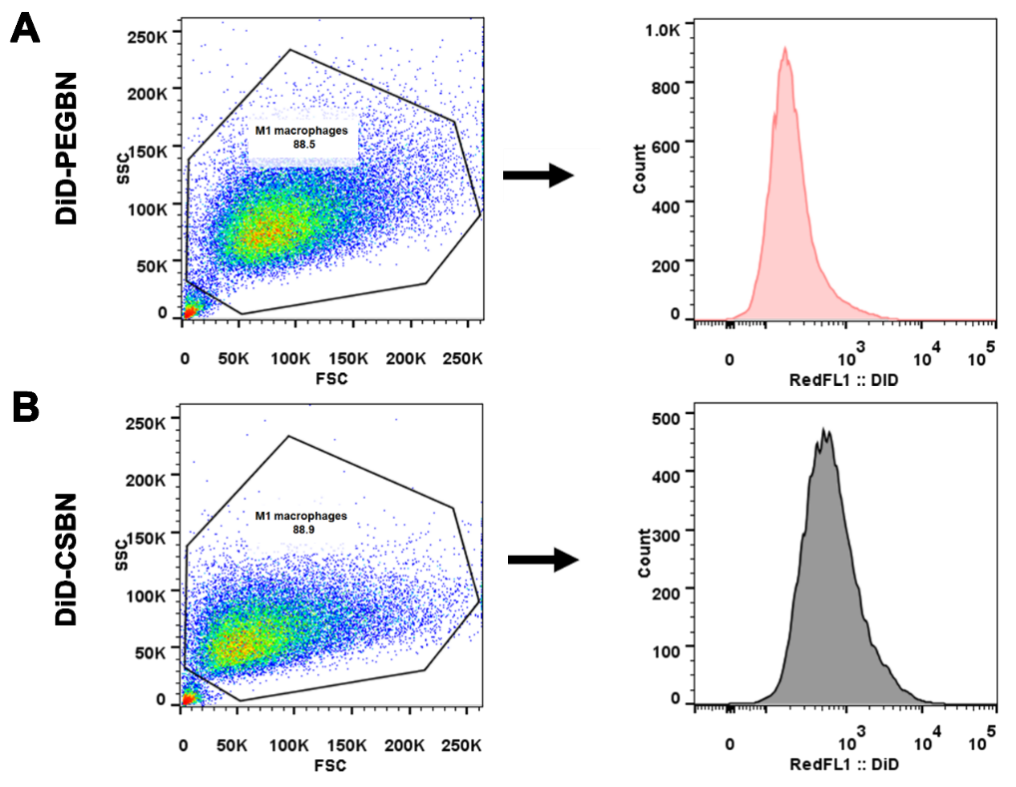
**

**Figure S8.** Flow cytometry gating strategy for cellular uptake assay in M1 macrophages. A,B) Flow cytometry gating strategy for DiD-PEGBN (A) and DiD-CSBN (B), a forward/side scatter dot plot (left) was used to gate the main cell population, and the gated cells were then analyzed by RedFL1: DiD and count staining patterns (right).


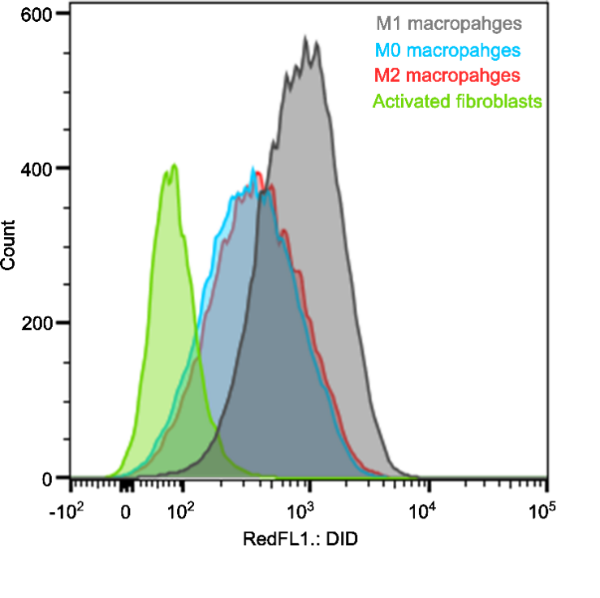


**Figure S9.** Flow cytometric histogram images of cellular uptake of DiD-CSBN in M0, M1, M2 macrophages, and activated fibroblasts.

**
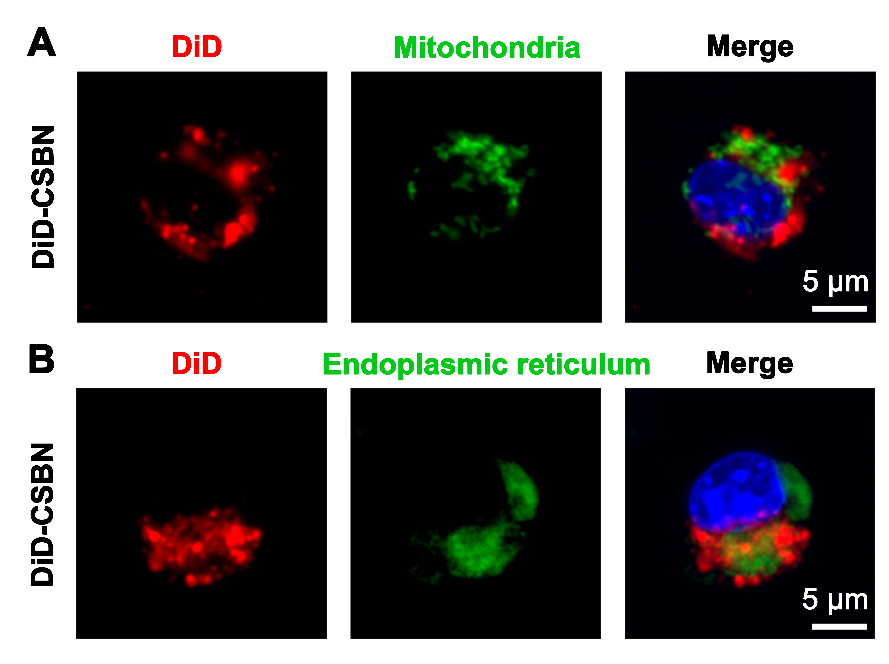
**

**Figure S10.** A,B) Representative colocalization images of DiD-CSBN with mitochondria (A) or the endoplasmic reticulum (B) in M1 macrophages.


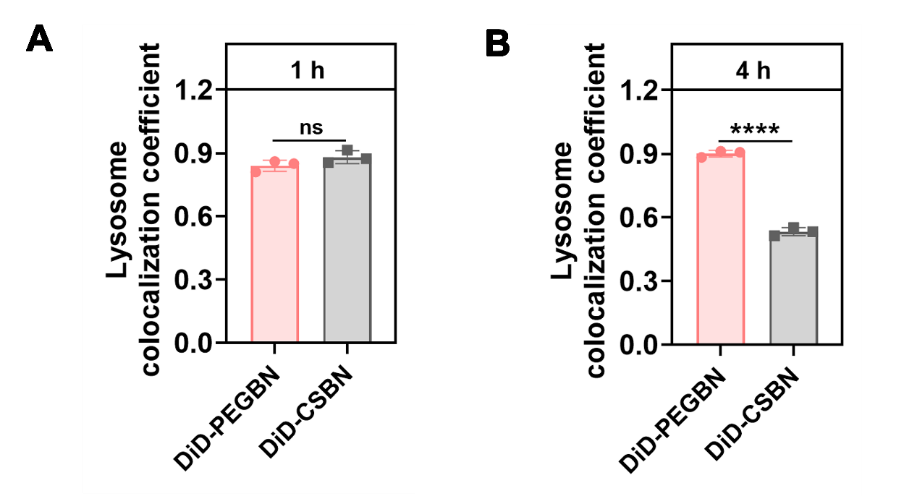


**Figure S11.** Colocalization ratios of nanomedicines with lysosomes. A,B) Colocalization coefficient of DiD-labeled nanomedicines with lysosomes in M1 macrophages at 1 h (A) and 4 h (B) measured by Pearson’s correlation coefficient (*n* = 3, mean ± SD). ns = no significance, *********P* < 0.0001, as determined by student’s two-sided t test (A and B).


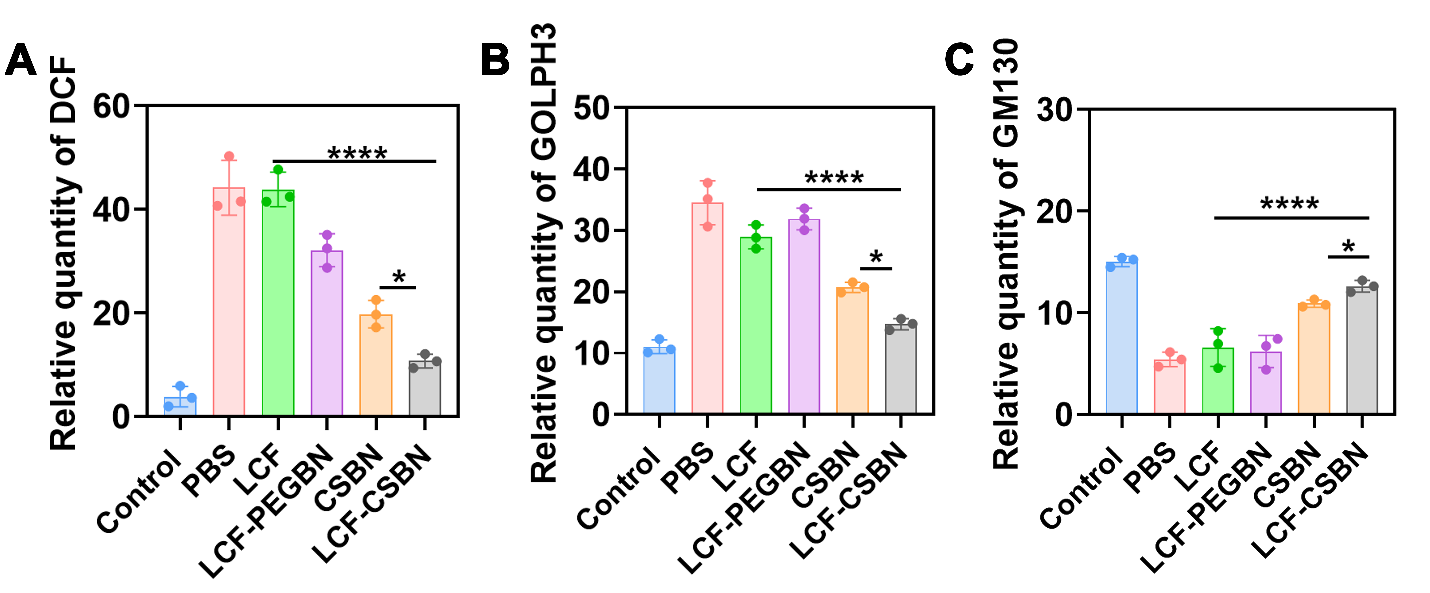


**Figure S12.** Quantitative analysis of DCF, GOLPH3, and GM130. A-C) Relative quantity of DCF (A), GOLPH3 (B), and GM130 (C) in M1 macrophages from different groups (*n* = 3, mean ± SD). ******P* < 0.05, *********P* < 0.0001, as determined by one-way ANOVA with Tukey’s post hoc test (A-C).


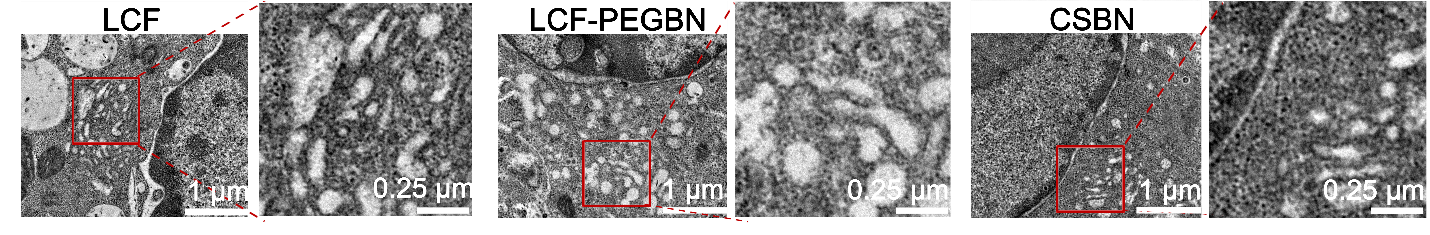


**Figure S13.** Representative TEM images of the Golgi apparatus in M1 macrophages receiving different treatments. Morphological changes of Golgi apparatus in M1 macrophages after treatment with LCF, LCF-PEGBN, and CSBN, red box indicates the Golgi apparatus.


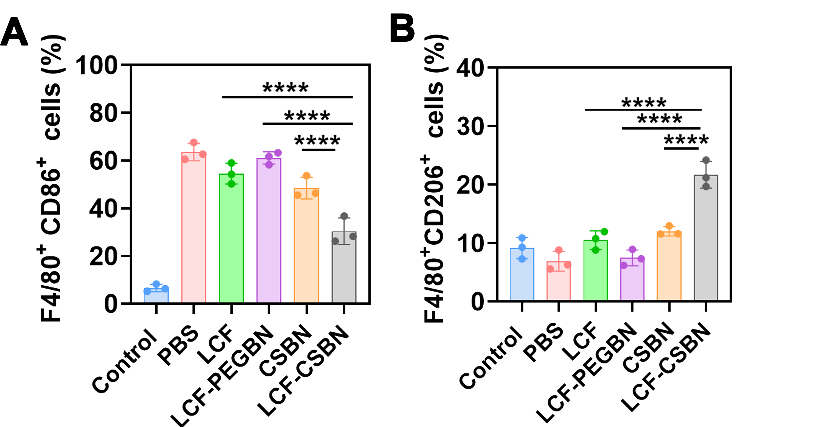


**Figure S14.** LCF-CSBN repolarizes M1 macrophages to M2 phenotype in LPS-stimulated BMDMs. A,B) Quantification analysis of M1 macrophages (A) and M2 macrophages (B) in LPS-stimulated BMDMs from different groups by flow cytometry assay (*n* = 3, mean ± SD). *********P* < 0.0001, as determined by one-way ANOVA with Tukey’s post hoc test (A and B).


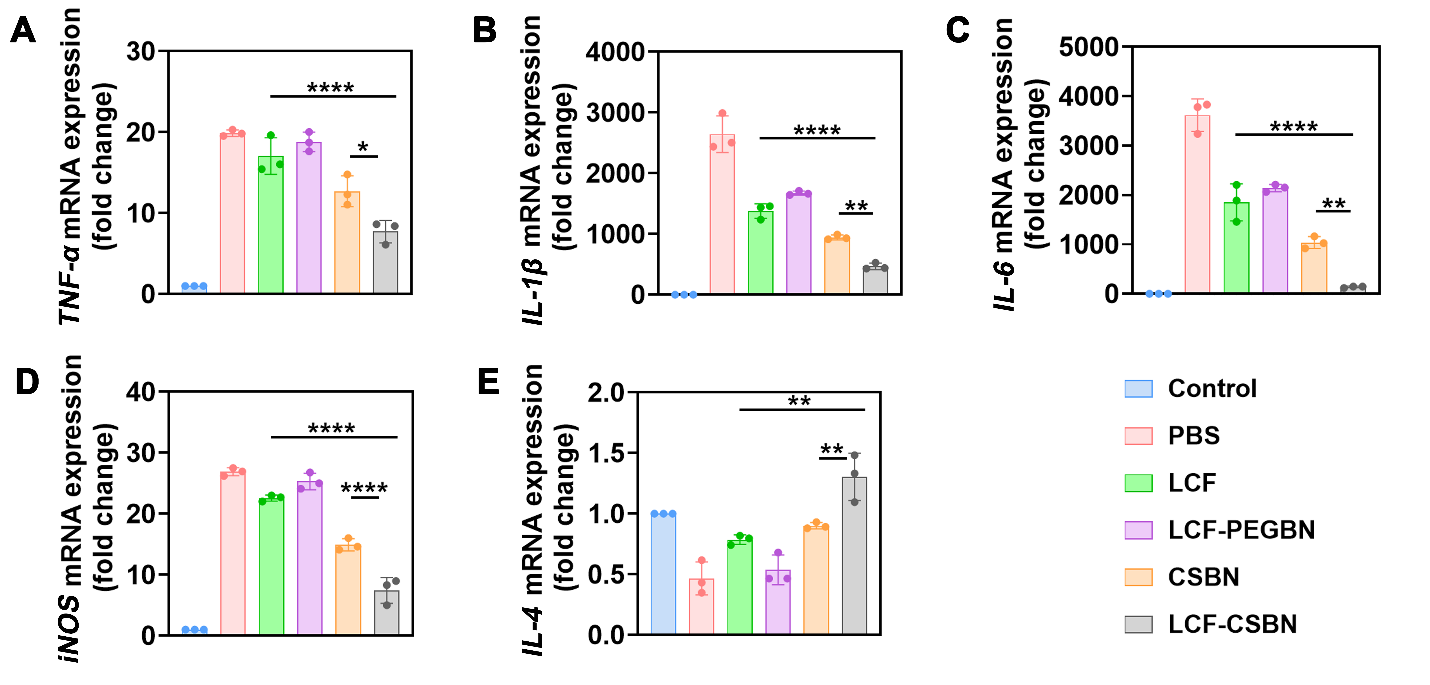


**Figure S15.** mRNA expressions of inflammatory cytokines in M1 macrophages after different treatments. A-E) Relative mRNA expressions of *TNF-α* (A), *IL-1β* (B), *IL-6* (C), *iNOS* (D) and *IL-4* (E) (*n* = 3, mean ± SD), ******P* < 0.05, *******P* < 0.01, *********P* < 0.0001, as determined by one-way ANOVA with Tukey’s post hoc test (A-E).


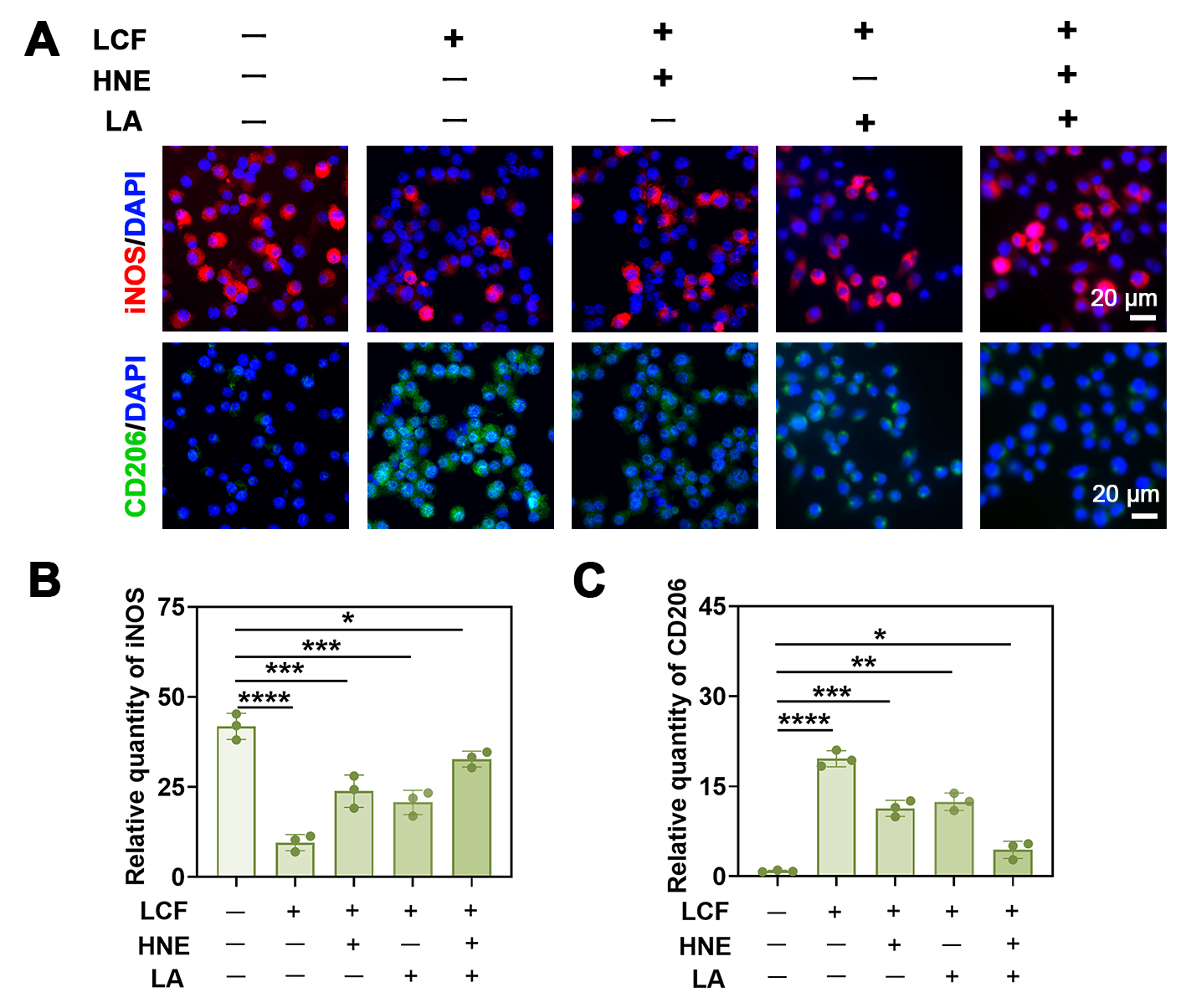


**Figure S16.** A) Representative coimmunostaining images of iNOS (M1 phenotype) and CD206 (M2 phenotype) in macrophages from the rescue experiment for the inhibited COX-2 or 5-LOX by using 4-hydroxy-2-nonenal (HNE) or linoleic acid (LA), following LCF treatment. B,C) Semi-quantitative and statistical analyses of the relative quantity of iNOS (B) and CD206 (C) (*n* = 3, mean ± SD), ******P* < 0.05, *******P* < 0.01, ********P* < 0.001, *********P* < 0.0001, as determined by one-way ANOVA (multiple comparisons) with Tukey’s post hoc test (B and C).


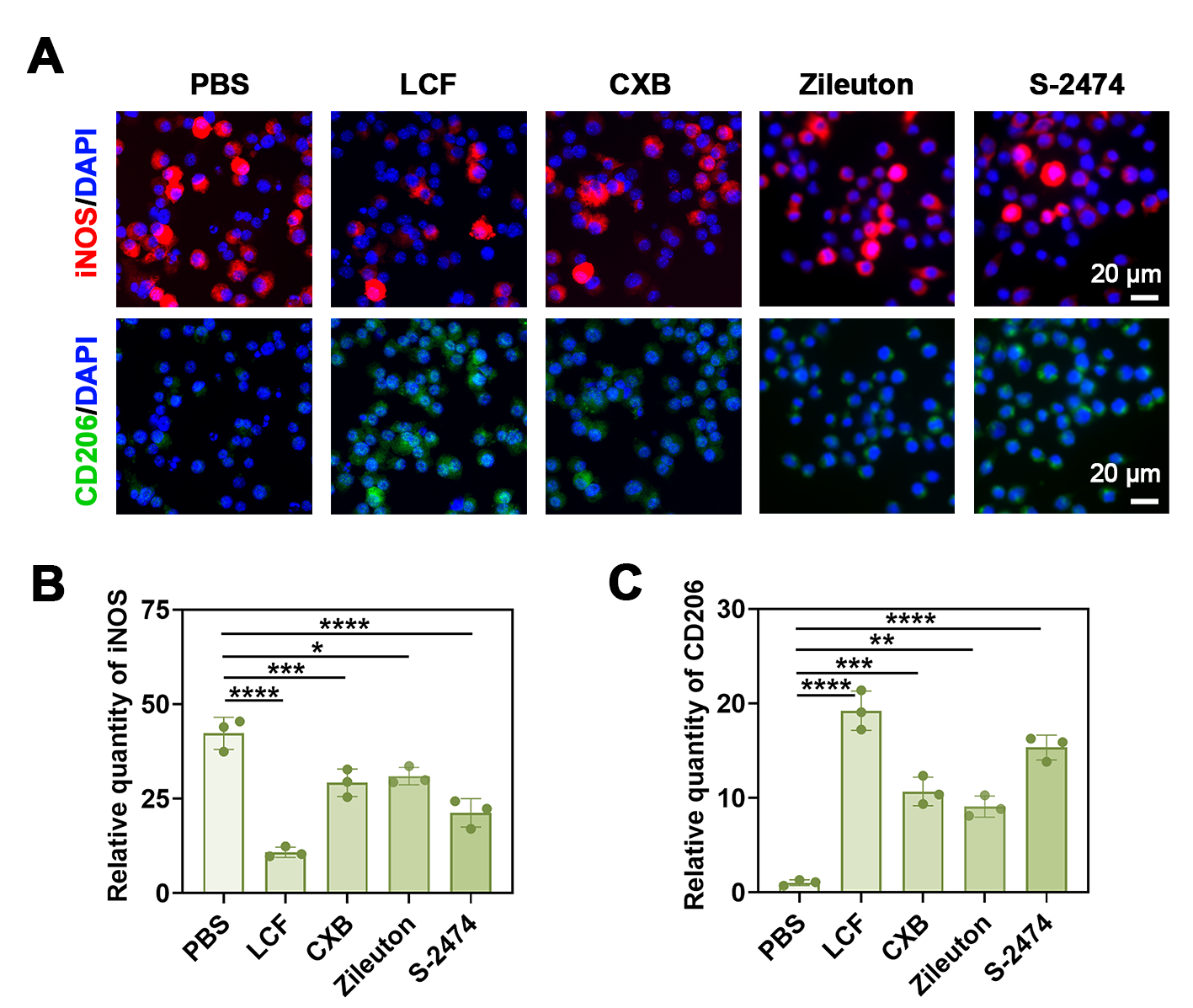


**Figure S17.** A) Representative coimmunostaining images of iNOS (M1 phenotype) and CD206 (M2 phenotype) demonstrating the repolarization efficiency in M1 macrophages following treatment with PBS, LCF, celecoxib (CXB), Zileuton, or S-2474. B,C) Semi-quantitative and statistical analyses of the relative quantity of iNOS (B) and CD206 (C) (*n* = 3, mean ± SD), ******P* < 0.05, *******P* < 0.01, ********P* < 0.001, *********P* < 0.0001, as determined by one-way ANOVA (multiple comparisons) with Tukey’s post hoc test (B and C).


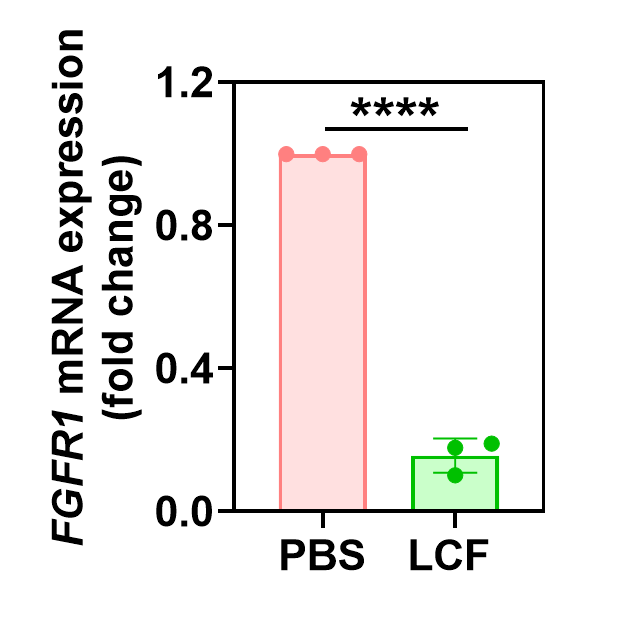


**Figure S18.** Relative mRNA expressions of *FGFR1* in M1 macrophage after treatment with PBS or LCF (*n* = 3, mean ± SD), *********P* < 0.0001, as determined by student’s two-sided t test.


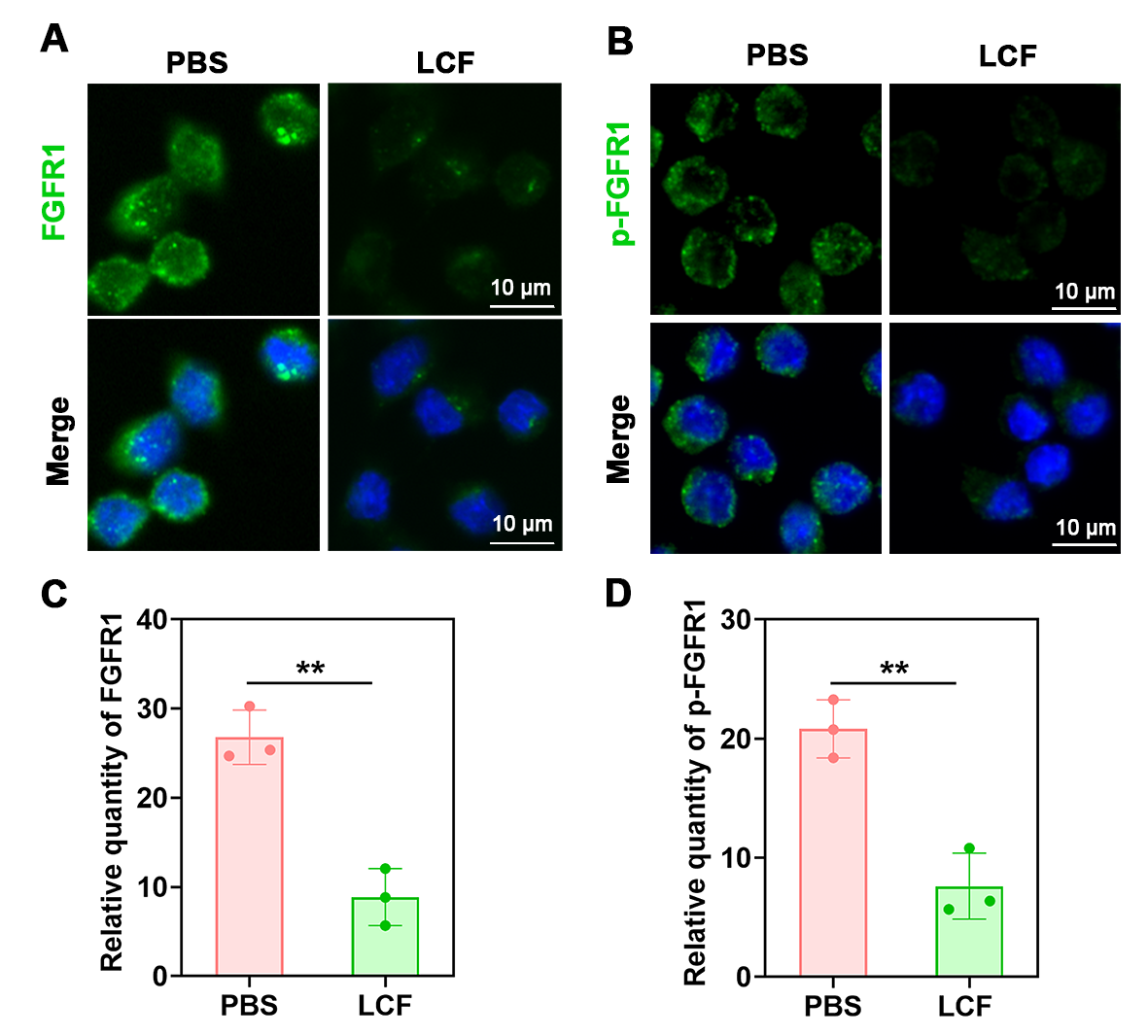


**Figure S19.** A,B) Representative fluorescence images of FGFR1 (A) and p-FGFR1 (B) in M1 macrophages following treatment with PBS or LCF. C,D) Semi-quantitative and statistical analyses of the relative quantity of FGFR1 (C) and p-FGFR1 (D) (*n* = 3, mean ± SD), *******P* < 0.01, as determined by student’s two-sided t test (C and D).


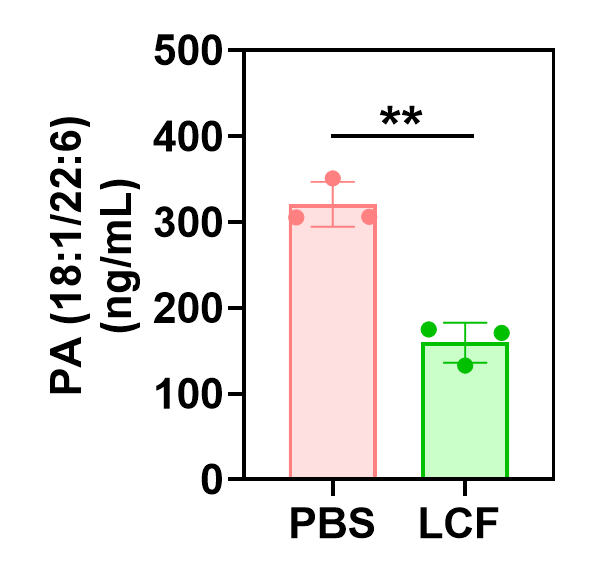


**Figure S20.** The levels of PA (18:1/22:6) in M1 macrophages treated with PBS and LCF were measured by targeted HPLC-ESI-MS/MS (*n* = 3, mean ± SD), *******P* < 0.01, as determined by student’s two-sided t test.


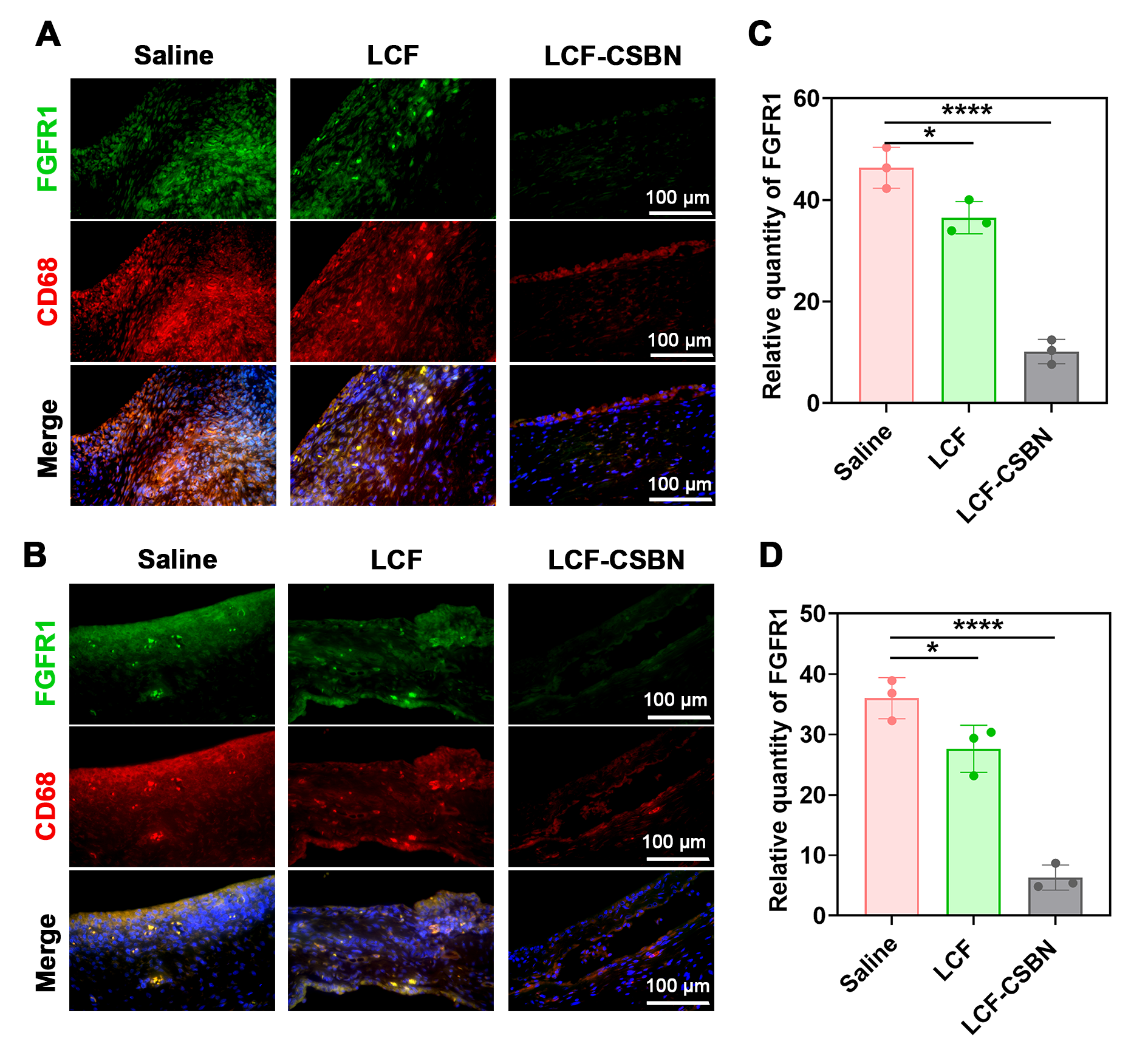


**Figure S21.** A,B) Representative coimmunostaining images of FGFR1 in synovial macrophages from different groups of MIA rats (A) and ACLT+pMMx rats (B), synovial macrophages were stained with CD68 antibody (red). C,D) Semi-quantitative and statistical analyses of the relative quantity of FGFR1 in synovial macrophages from MIA rats (C) and ACLT+pMMx rats (D) (*n* = 3, mean ± SD), ******P* < 0.05, *********P* < 0.0001, as determined by one-way ANOVA (multiple comparisons) with Tukey’s post hoc test (C and D).


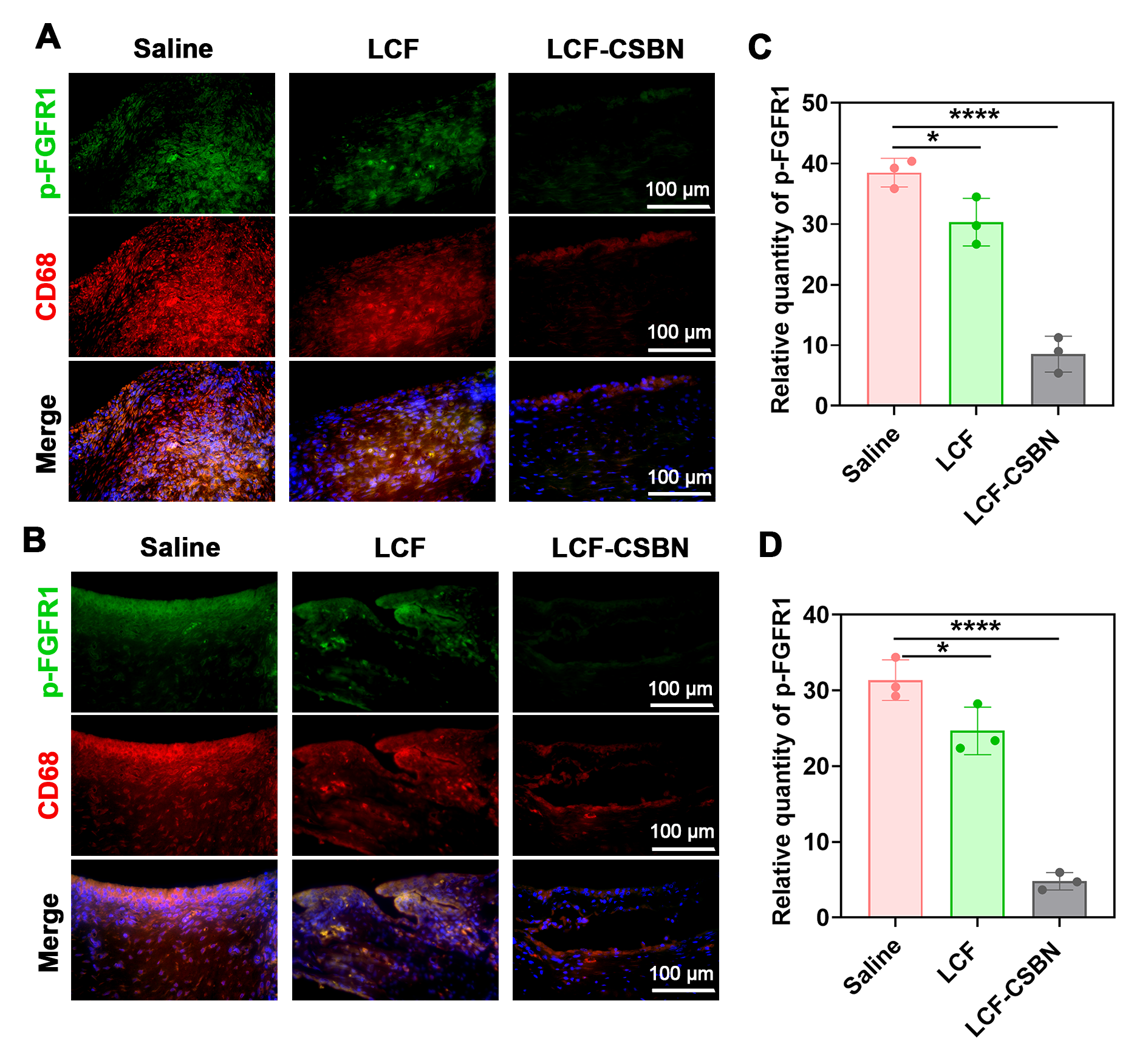


**Figure S22.** A,B) Representative coimmunostaining images of p-FGFR1 in synovial macrophages from different groups of MIA rats (A) and ACLT+pMMx rats (B), synovial macrophages were stained with CD68 antibody (red). C,D) Semi-quantitative and statistical analyses of the relative quantity of p-FGFR1 in synovial macrophages from MIA rats (C) and ACLT+pMMx rats (D) (*n* = 3, mean ± SD), ******P* < 0.05, *********P* < 0.0001, as determined by one-way ANOVA (multiple comparisons) with Tukey’s post hoc test (C and D).


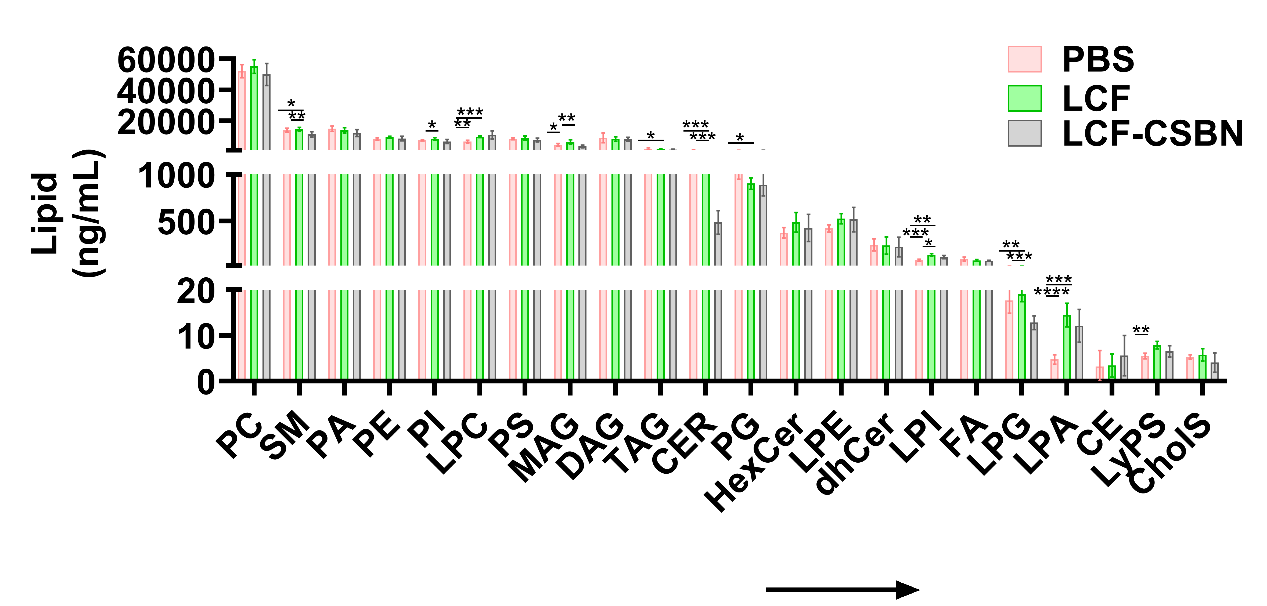


**Figure S23.** Quantitative analysis of lipid in M1 macrophages from different groups. Targeted lipidomics on M1 macrophages in PBS group, LCF group, and LCF-CSBN. Content of lipid classes includes PC, SM, PA, PE, PI, LPC, PS, MAG, DAG, TAG, CER, PG, HexCer, LPE, dhCer, LPI, FA, LPG, LPA, CE, LyPS, and CholS (*n* =3, mean ± SD). ******P* < 0.05, *******P* < 0.01, ********P* < 0.001, *********P* < 0.0001, as determined by one-way ANOVA with Tukey’s post hoc test.


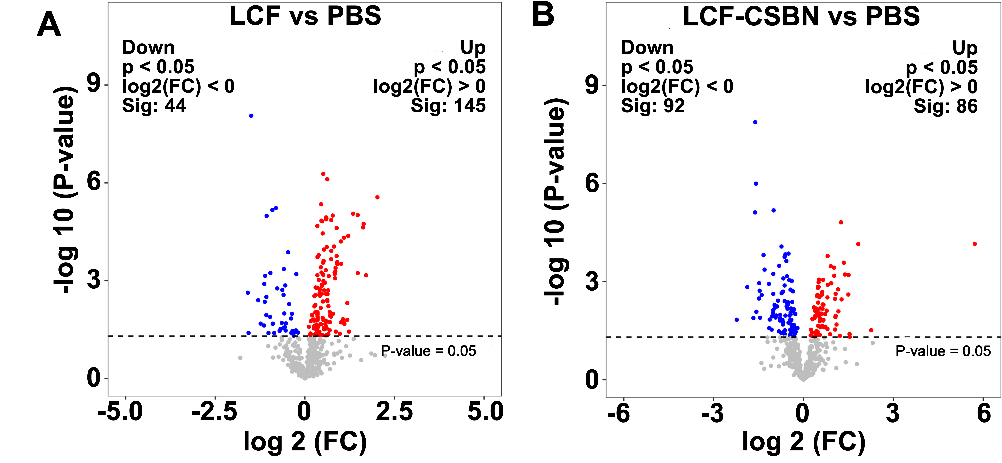


**Figure S24.** Volcano maps of regulated lipid species in different pairwise comparisons. A) Significantly upregulated (red) and downregulated (blue) lipid species between LCF group and PBS group. B) Significantly upregulated (red) and downregulated (blue) lipid species between LCF-CSBN group and PBS group.


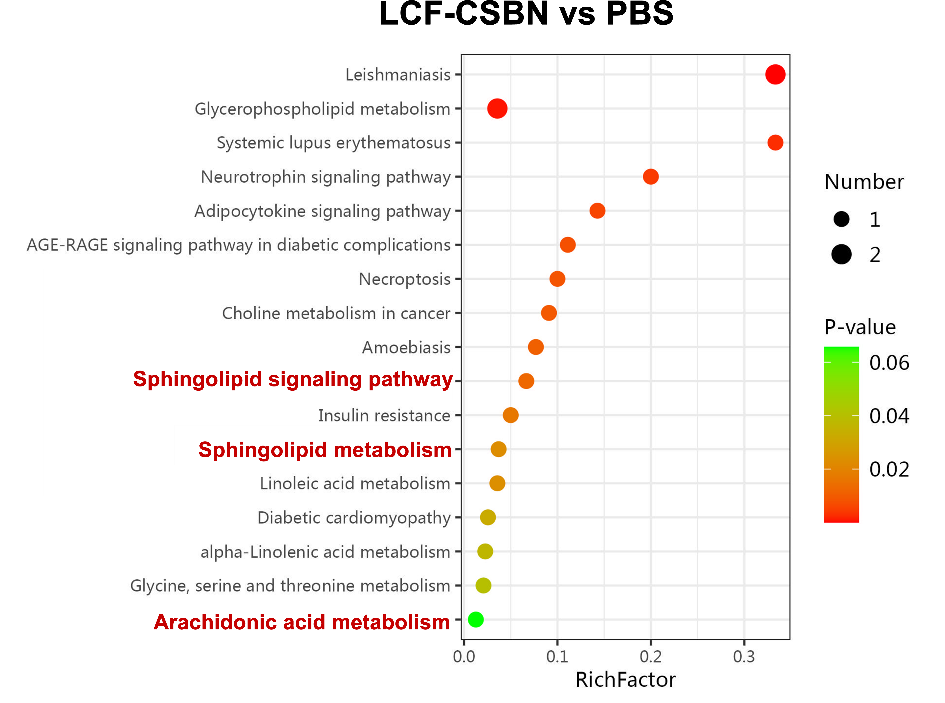


**Figure S25.** LCF-CSBN significantly regulates sphingolipid and AA metabolism in M1 macrophages. The top 20 KEGG pathways of significantly downregulated lipid species between LCF-CSBN group and PBS group.


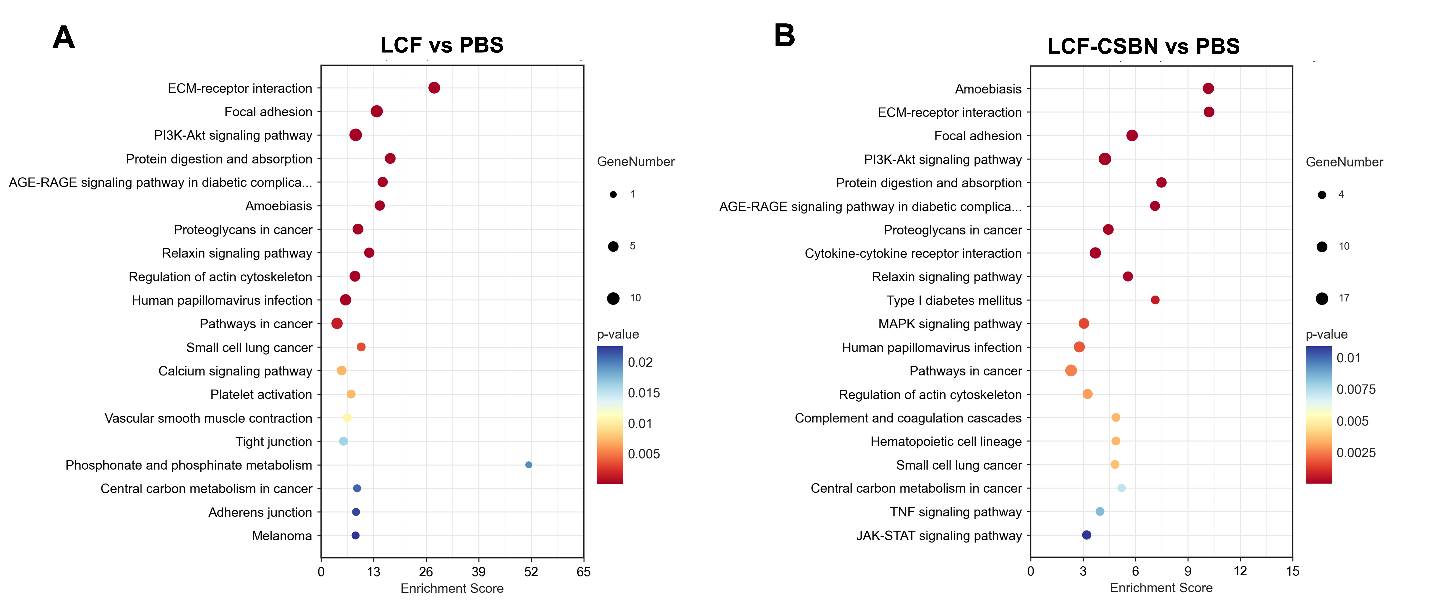


**Figure S26.** Analysis of the DEGs enrichment within the KEGG pathway. A) The top 20 KEGG pathways of downregulated DEGs in M1 macrophages between LCF group and PBS group. B) The top 20 KEGG pathways of downregulated DEGs in M1 macrophages between LCF-CSBN group and PBS group.


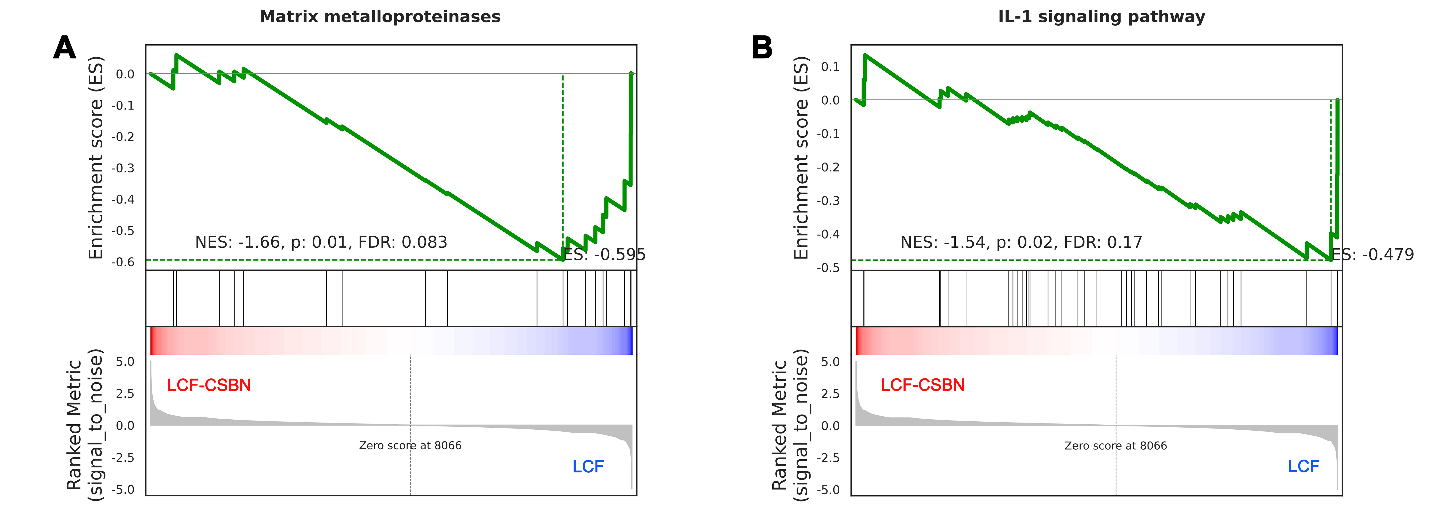


**Figure S27.** Representative enrichment plots of GSEA. A,B) GSEA showcasing downregulation of matrix metalloproteinases (A) and IL-1 signaling pathway (B) in M1 macrophages treated with LCF-CSBN.


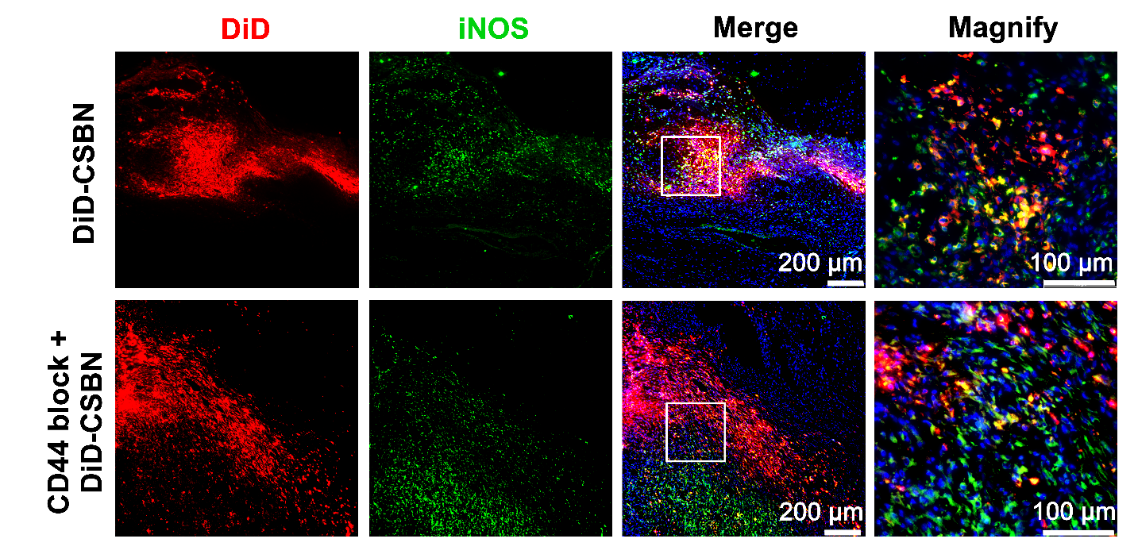


**Figure S28.** LCF-CSBN targets synovial M1 macrophages via CD44 receptor *in vivo.* Representative coimmunostaining images showing the distribution of DiD-CSBN (red) with or without CD44 block in synovial M1 macrophages of MIA rats, synovial M1 macrophages were stained with iNOS antibody (green).


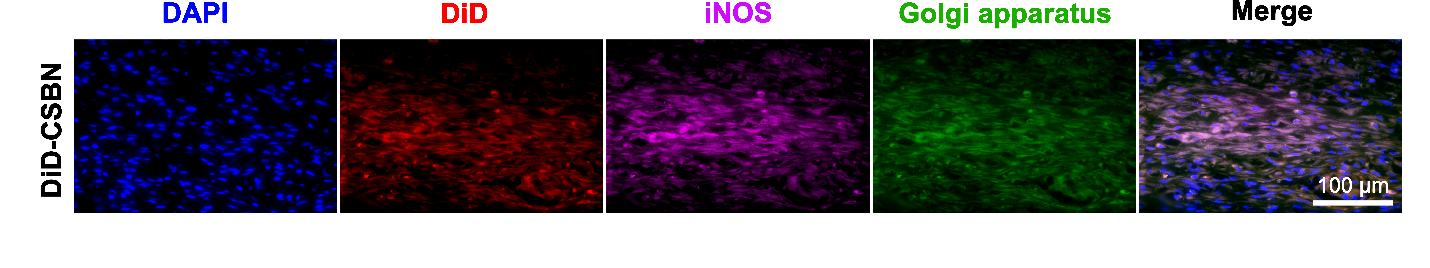


**Figure S29.** LCF-CSBN accumulates in Golgi apparatus of synovial M1 macrophages of MIA rats. Representative coimmunostaining images of DiD-CSBN, iNOS and BODIPY TR CER complexed to BSA in synovium of MIA rats, M1 macrophages and Golgi apparatus were stained with iNOS antibody (magenta) and BODIPY TR CER complexed to BSA antibody (green), respectively.


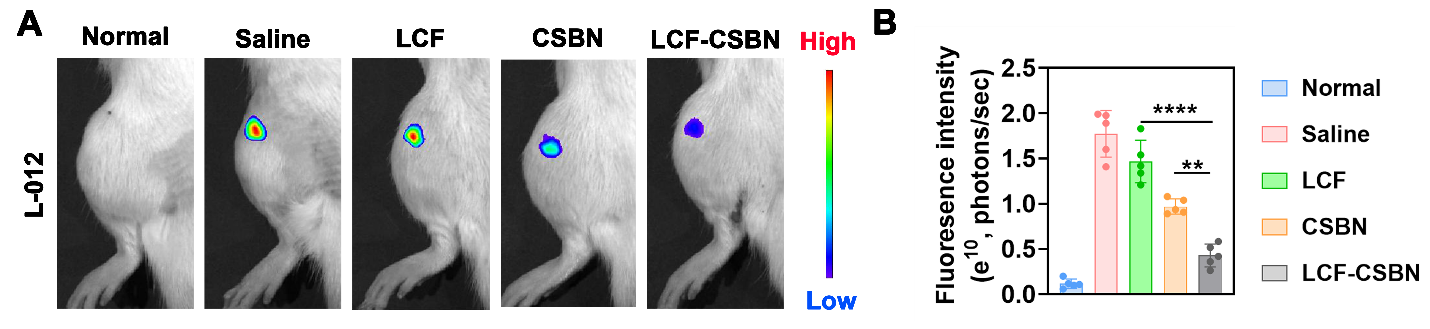


**Figure S30.** LCF-CSBN reduces ROS levels in knee joints of MIA rats. A,B) The levels of ROS in knee joints of MIA rats after different treatments, as shown by the IVIS images (A) and corresponding L-012 intensities of ROS (B). *******P* < 0.01, *********P* < 0.0001, as determined by one-way ANOVA with Tukey’s post hoc test (B).


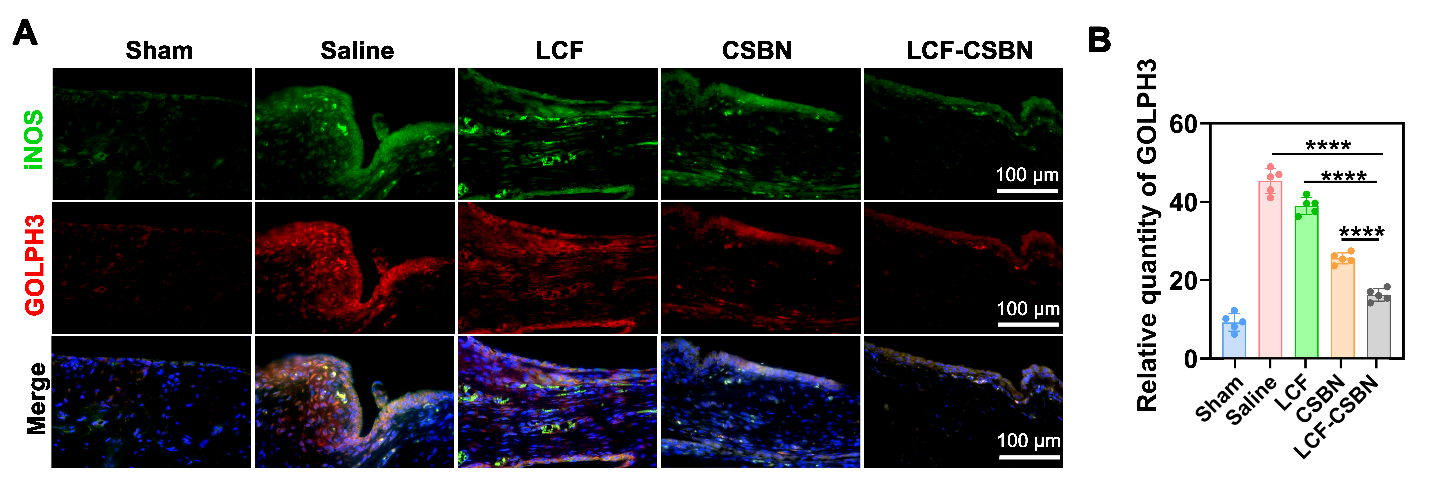


**Figure S31.** LCF-CSBN reduces the expression of GOLPH3 in synovial M1 macrophages of ACLT+pMMx rats. A,B) Representative coimmunostaining images of GOLPH3 in synovial M1 macrophages from different groups (A), and the relative quantity of GOLPH3 (B), synovial M1 macrophages were stained with iNOS antibody (*n* = 5, mean ± SD). *********P* < 0.0001, as determined by one-way ANOVA (multiple comparisons) with Tukey’s post hoc test (B).


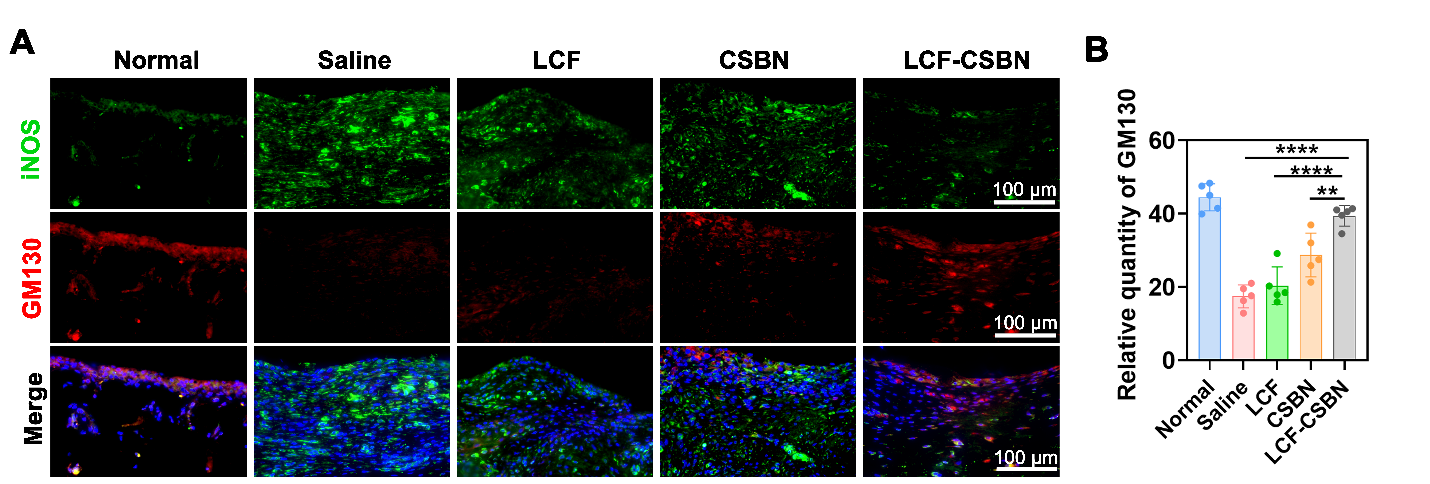


**Figure S32.** LCF-CSBN upregulates the expression of GM130 in synovial M1 macrophages of MIA rats. A,B) Representative coimmunostaining images of GM130 in synovial M1 macrophages from different groups (A), and the relative quantity of GM130 (B), synovial M1 macrophages were stained with iNOS antibody (*n* = 5, mean ± SD). *******P* < 0.01, *********P* < 0.0001, as determined by one-way ANOVA with Tukey’s post hoc test (B).


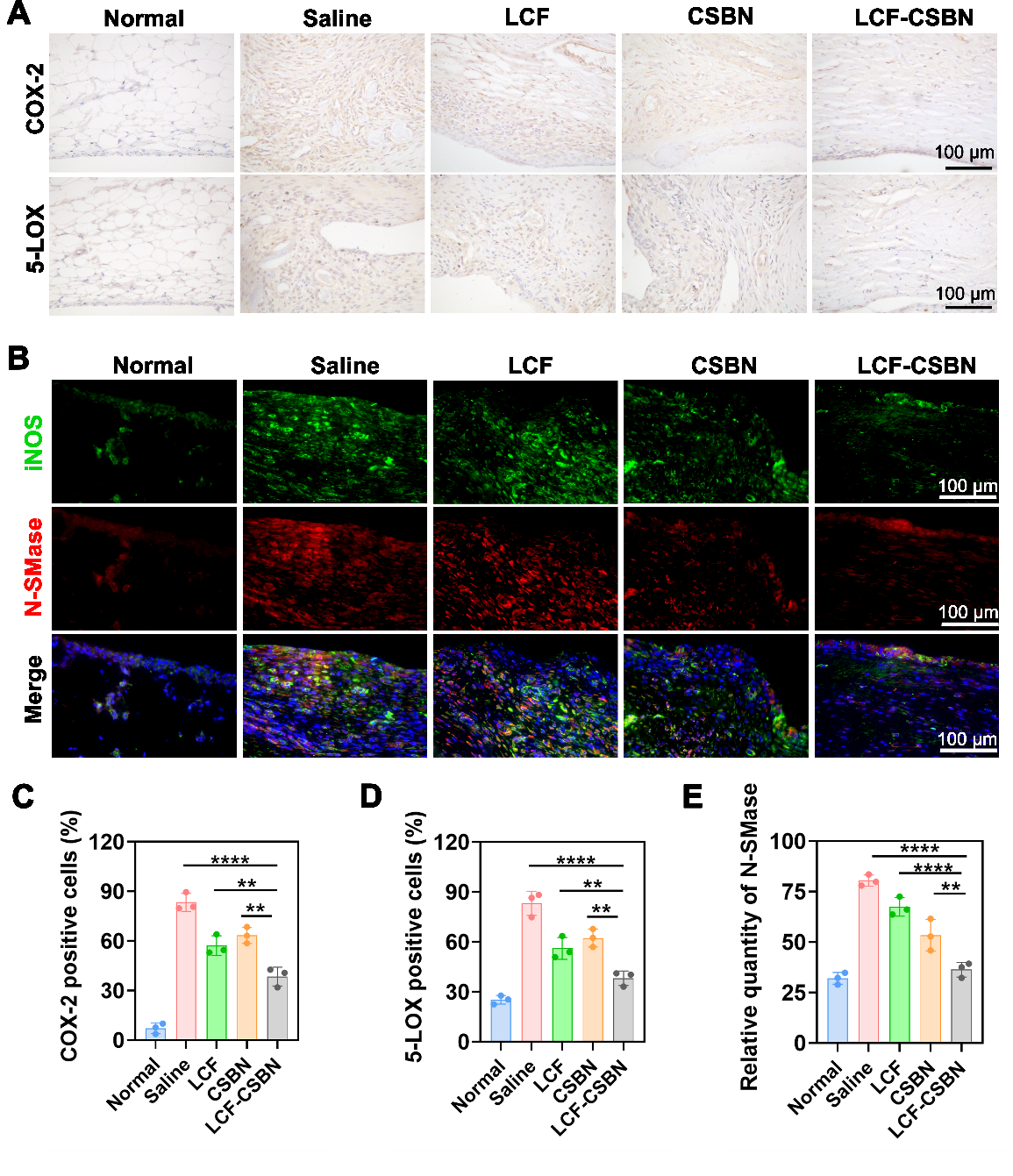


**Figure S33.** LCF-CSBN reprograms sphingolipid and arachidonic acid metabolism *in vivo.*

A) Representative IHC staining images of COX-2 and 5-LOX in the synovium of MIA rats from different groups. B) Representative coimmunostaining images of N-SMase in synovial M1 macrophages of MIA rats from different groups, synovial M1 macrophages were stained with iNOS antibody. C-E) Semi-quantitative and statistical analyses of the positive cells of COX-2 (C) and 5-LOX (D), as well as relative quantity of N-SMase (E) (*n* = 3, mean ± SD). *******P* < 0.01, *********P* < 0.0001, as determined by one-way ANOVA with Tukey’s post hoc test (C-E).


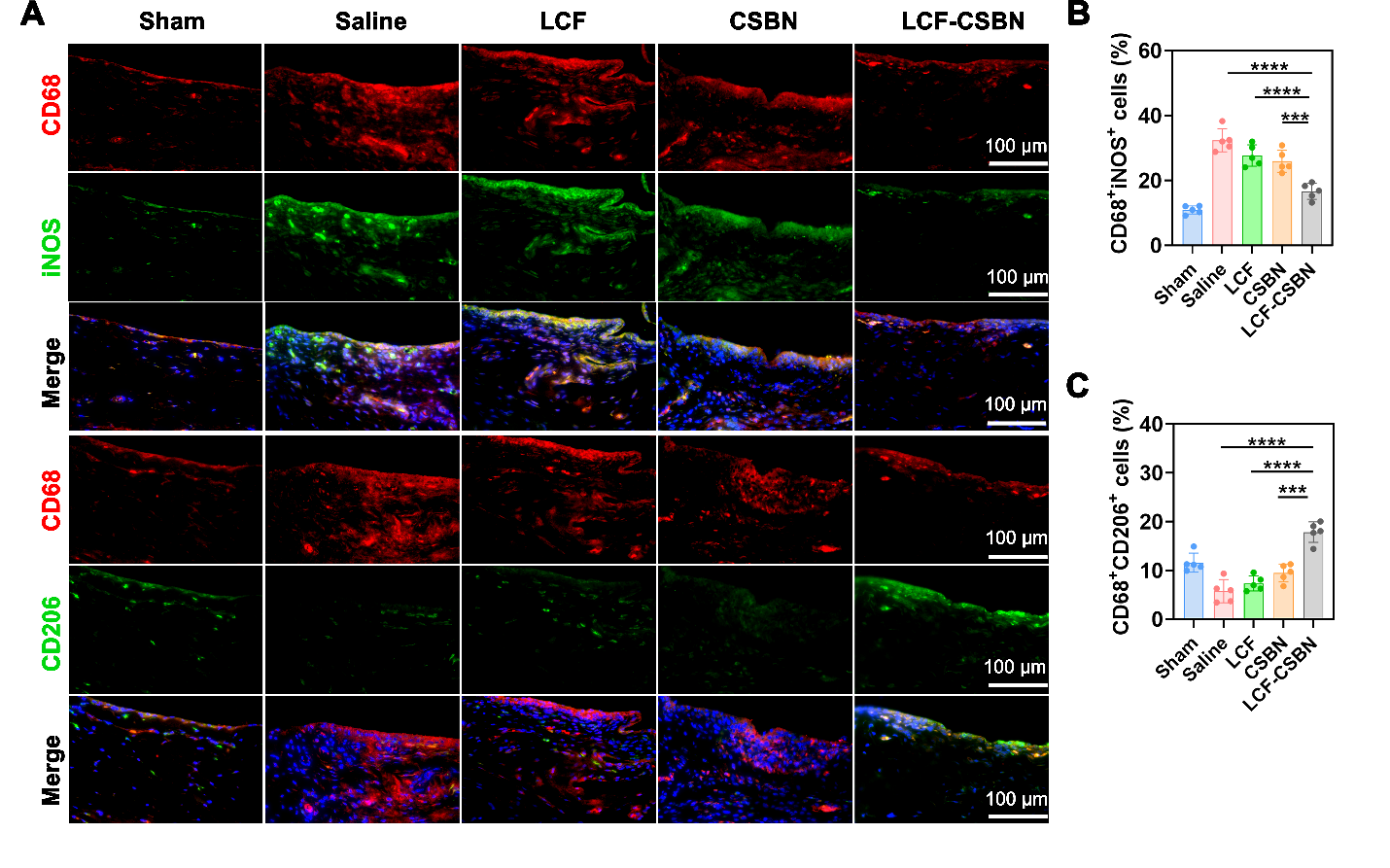


**Figure S34.** LCF-CSBN transforms M1 macrophages into M2 phenotype in synovium of ACLT+pMMx rats. A) Representative coimmunostaining images of CD68 and iNOS or CD206 in rat synovium indicating the repolarization efficiency in synovial macrophages from different groups, M1 and M2 macrophages were stained with iNOS (green) and CD206 (green), respectively. B,C) Quantitative analysis of M1 (B) and M2 (C) macrophages in synovium from different groups, respectively (*n* = 5, mean ± SD). ********P* < 0.001, *********P* < 0.0001, as determined by one-way ANOVA with Tukey’s post hoc test (B and C).


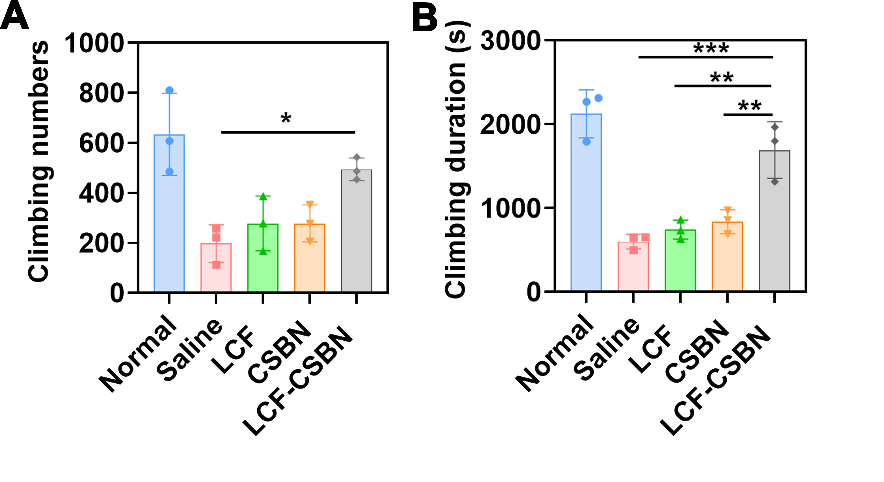


**Figure S35.** Quantification of spontaneous locomotor activity. A,B) The numbers of climbing attempts (A) and their duration (B) of MIA rats from different groups (*n* = 3, mean ± SD). ******P* < 0.05, *******P* < 0.01, ********P* < 0.001, as determined by one-way ANOVA with Tukey’s post hoc test (A and B).


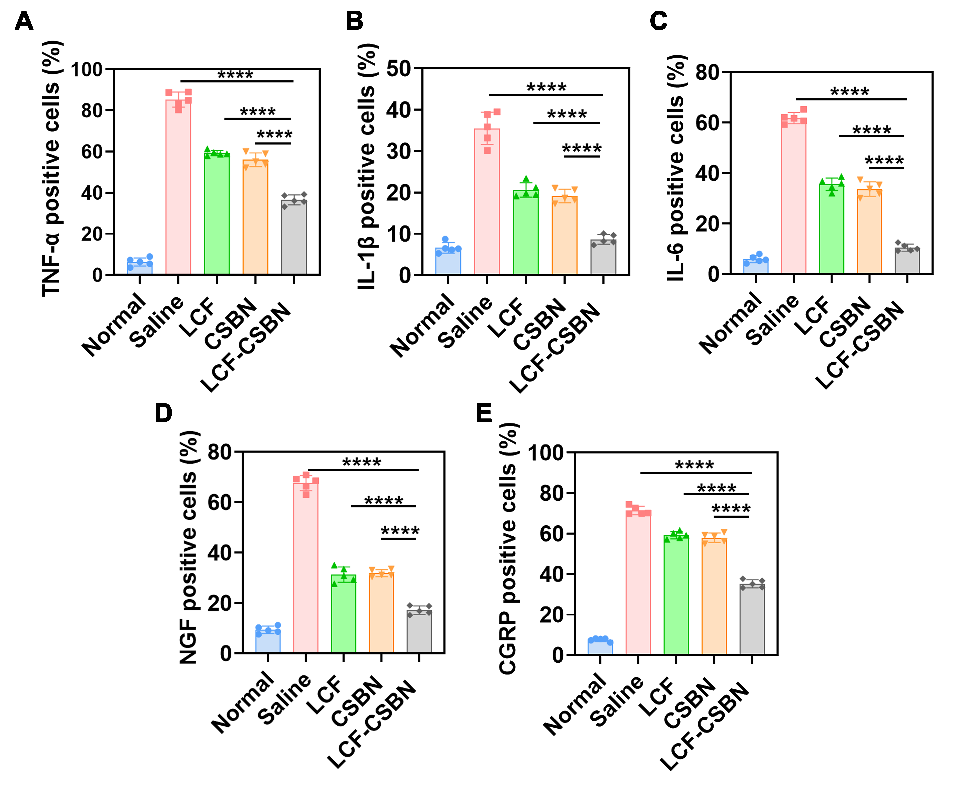


**Figure S36.** Quantitative analysis of the expressions of proinflammatory factors and pain sensitizers in synovium of MIA rats. A-E) Quantification of TNF-α positive cells (A), IL-1β positive cells (B), IL-6 positive cells (C), NGF positive cells (D), and CGRP positive cells (E) in synovium of MIA rats from different groups (*n* = 5, mean ± SD). ******** *P* < 0.0001, as determined by one-way ANOVA with Tukey’s post hoc test (A-E).


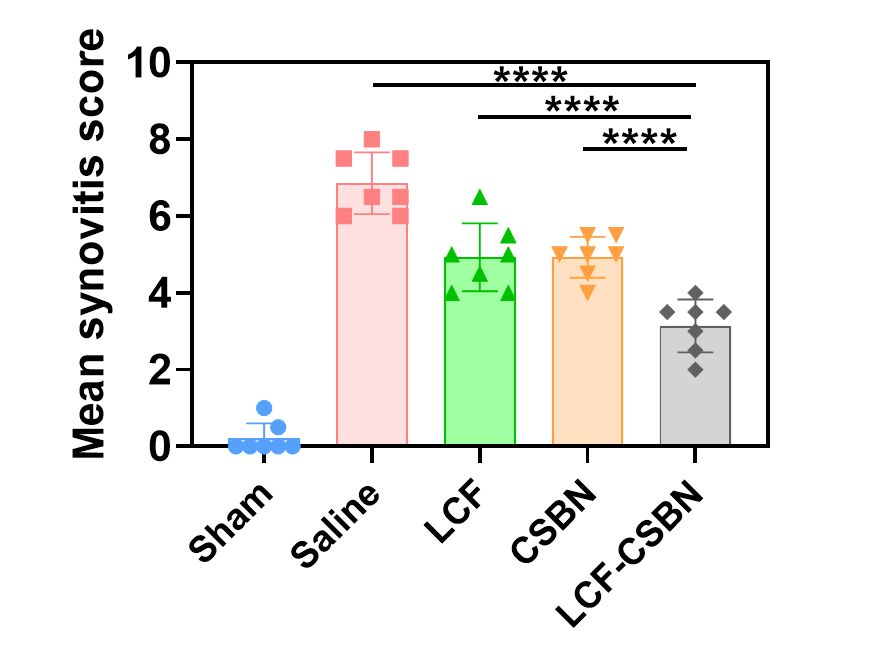


**Figure S37.** LCF-CSBN decreases synovitis score in synovium of ACLT+pMMx rats. Quantification of synovitis score in synovium of ACLT+pMMx rats from different groups (*n* = 7, mean ± SD). *********P* < 0.0001, as determined by one-way ANOVA with Tukey’s post hoc test.


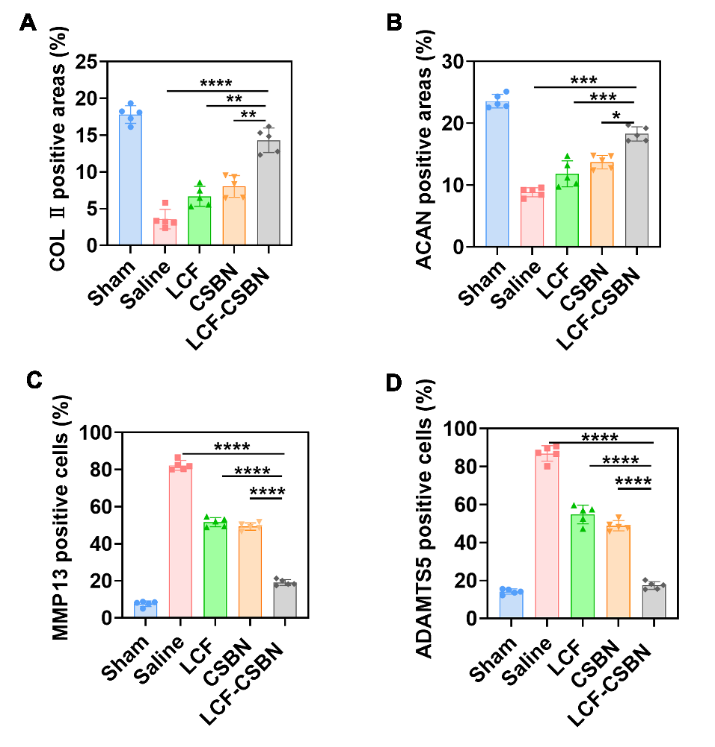


**Figure S38.** Quantitative analysis of the expressions of synthetic factors and catabolic factors in cartilage of ACLT+pMMx rats. A-D) Quantification of COL Ⅱ positive areas (A), ACAN positive areas (B), MMP13 positive cells (C), and ADAMTS5 positive cells (D) in cartilage of ACLT+pMMx rats from different groups (*n* = 5, mean ± SD). ******P* < 0.05, *******P* < 0.01, ********P* < 0.001, *********P* < 0.0001, as determined by one-way ANOVA with Tukey’s post hoc test (A-D).


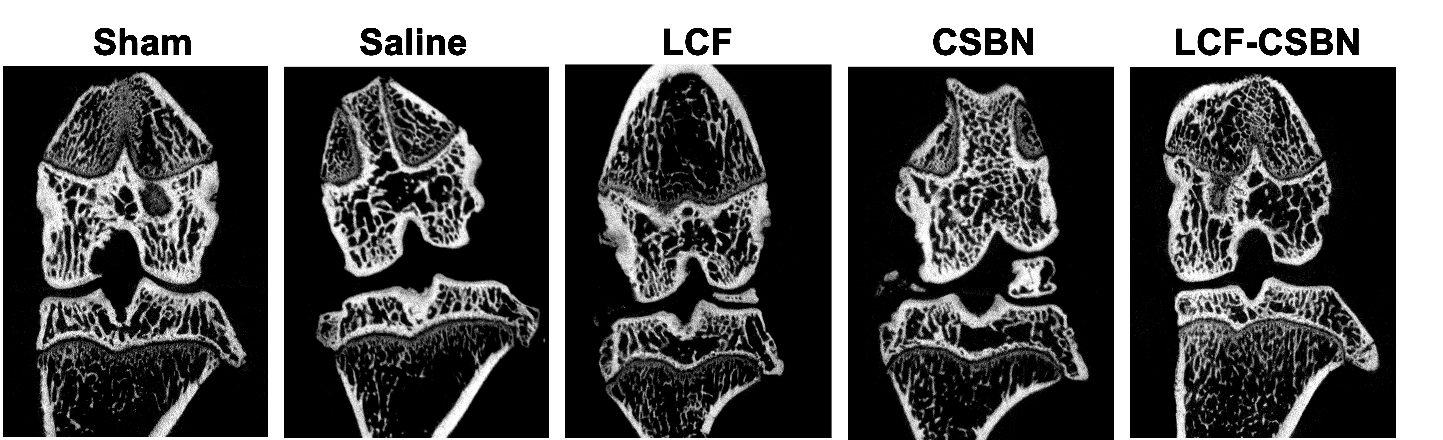


**Figure S39.** Representative 2D µCT images of knee joints in different groups.


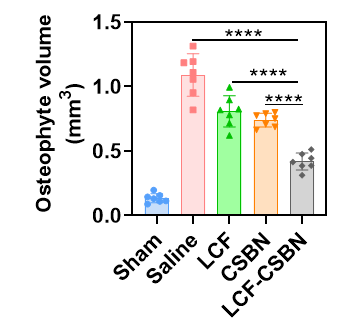


**Figure S40.** LCF-CSBN reduces osteophyte formation in ACLT+pMMx rats. Quantitative analysis of osteophyte volume of knee joints of ACLT+pMMx rats from different groups (*n* = 7, mean ± SD), *********P* < 0.0001, as determined by one-way ANOVA with Tukey’s post hoc test.


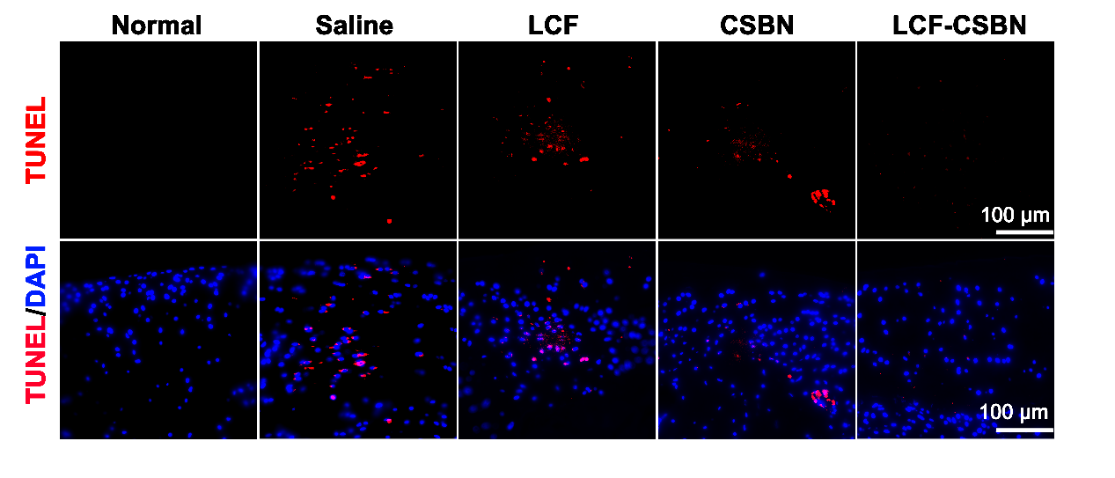


**Figure S41.** TUNEL staining in cartilage of MIA rats from different groups.


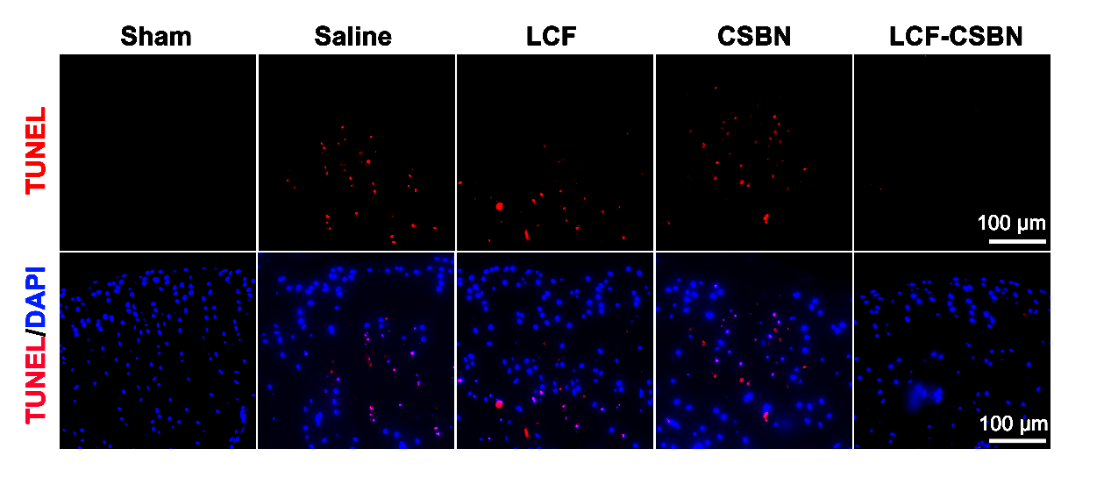


**Figure S42.** TUNEL staining in cartilage of ACLT+pMMx rats from different groups.


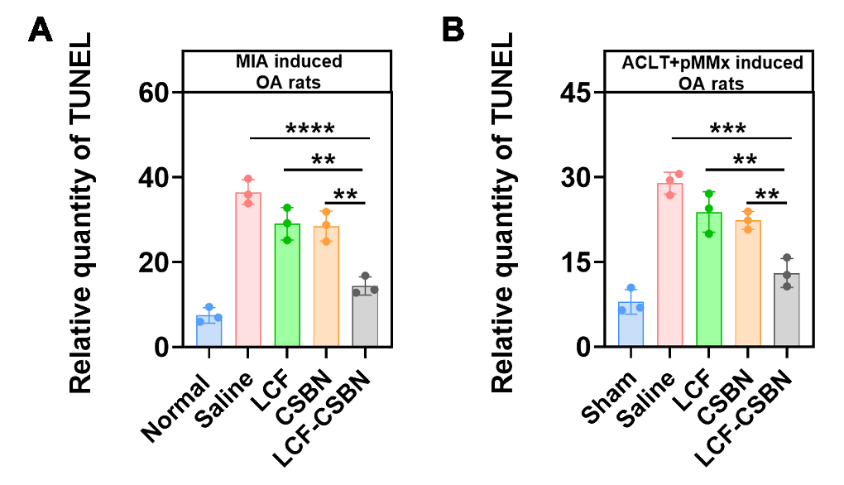


**Figure S43.** Quantitative analysis of TUNEL in cartilage of OA rats. A,B) The relative quantity of TUNEL in the cartilage of MIA rats (A) and ACLT+pMMx rats (B) from different groups (*n* = 3, mean ± SD). *******P* < 0.01, ********P* < 0.001, *********P* < 0.0001, as determined by one-way ANOVA with Tukey’s post hoc test (A and B).


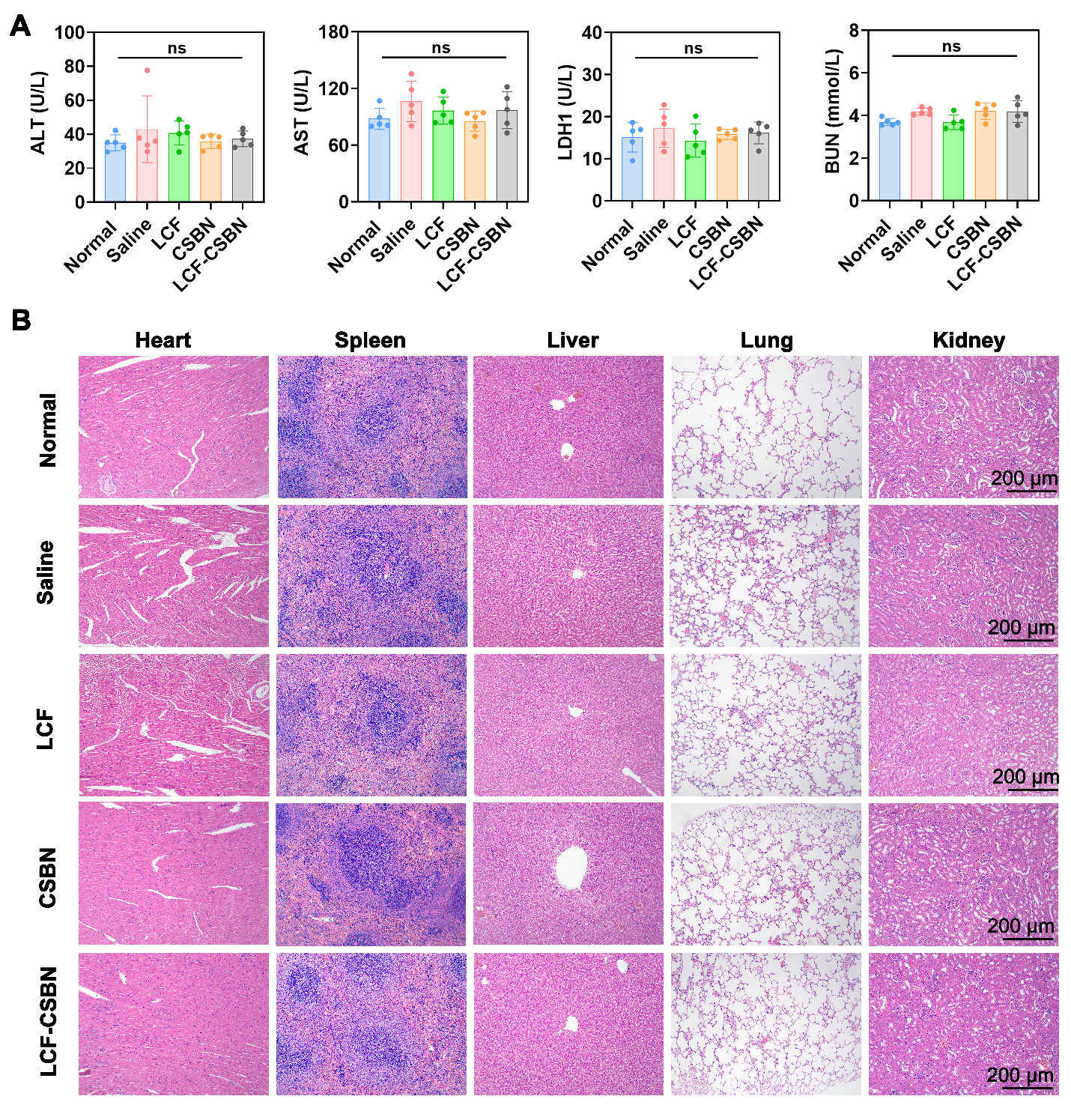


**Figure S44.** Safety evaluation of a single intra-articular injection of LCF-CSBN in MIA rats. A) The serum levels of ALT, AST, LDH1, and BUN in MIA rats from different groups (*n* = 5, mean ± SD). B) H&E staining of the major organs (heart, spleen, liver, lung, and kidney) was processed 4 weeks after rats receiving the indicated treatments. ns = no significance, as determined by one-way ANOVA with Tukey’s post hoc test (A).


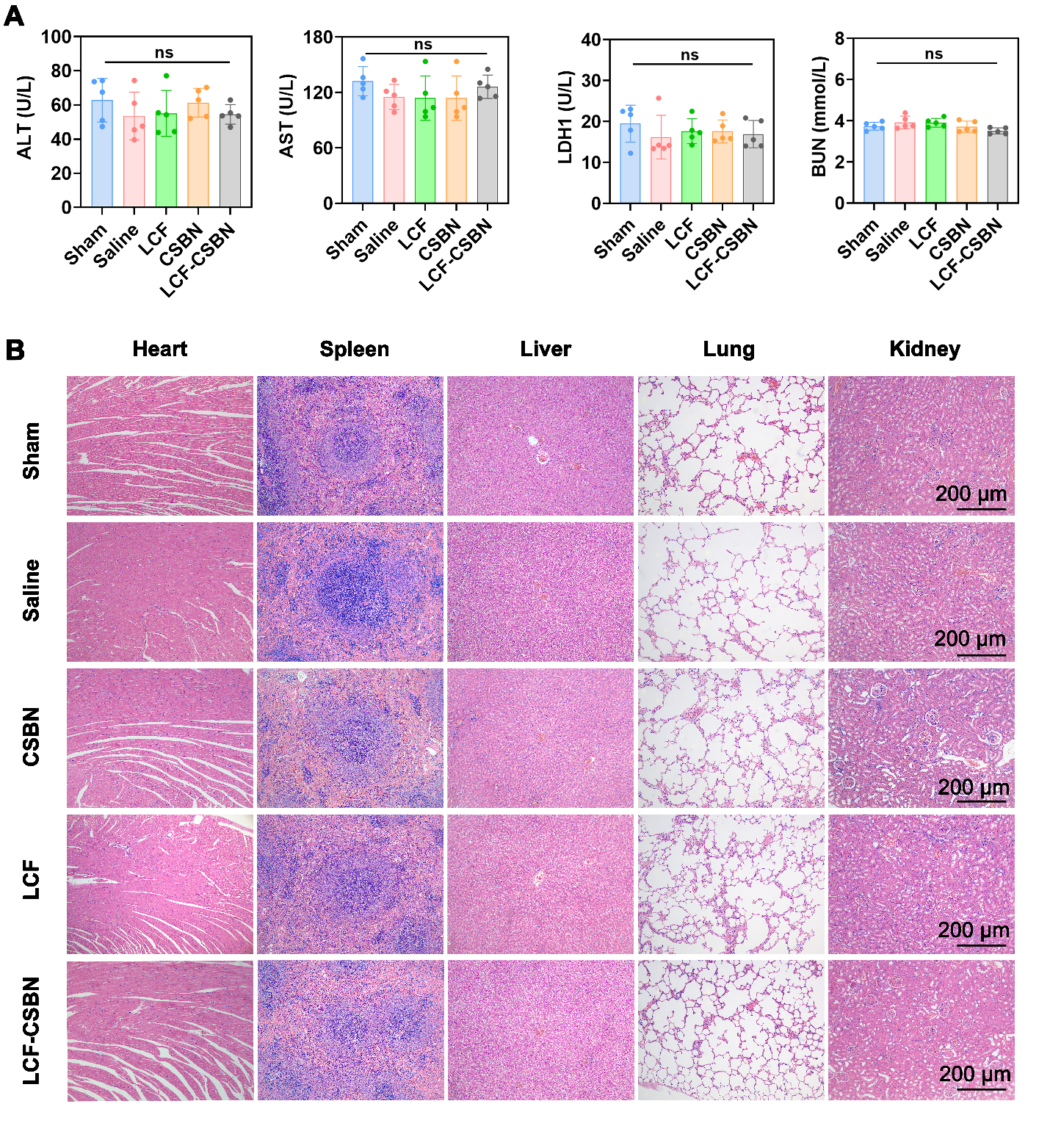


**Figure S45.** Long-term safety evaluation of repeated intra-articular injections of LCF-CSBN in ACLT+pMMx rats. A) The serum levels of ALT, AST, LDH1, and BUN in ACLT+pMMx rats from different groups (*n* = 5, mean ± SD). B) H&E staining of the major organs (heart, spleen, liver, lung, and kidney) was processed 8 weeks after rats receiving the indicated treatments. ns = no significance, as determined by one-way ANOVA with Tukey’s post hoc test (A).


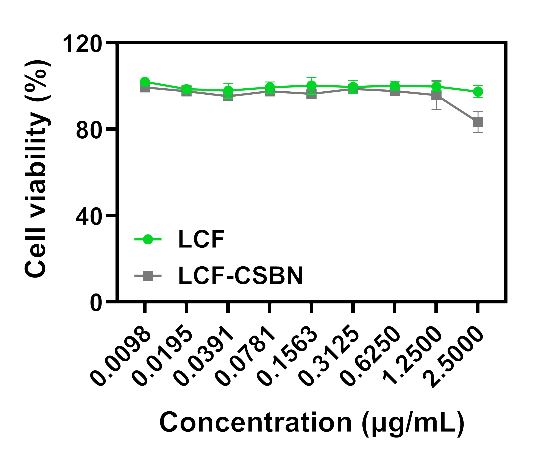


**Figure S46.** Cytotoxicity of LCF and LCF-CSBN in M1 macrophages was measured by CCK-8 assay (*n* = 3, mean ± SD).

**Table S1.** Physicochemical characteristics of LCF-PEGBN and LCF-CSBN

|  | Size  [nm] | PDI | | Zeta potential [mV] | Drug loading  [%] | Encapsulation efficiency [%] |
| --- | --- | --- | --- | --- | --- | --- |
| LCF-PEGBN | 157 ± 3.61 | | 0.256 ± 0.021 | -15.3 ± 2.70 | 4.31 ± 0.82 | 91.3 ± 5.32 |
| LCF-CSBN | 163 ± 3.29 | | 0.176 ± 0.019 | -35.3 ± 3.30 | 4.75 ± 0.76 | 94.3 ± 2.74 |

**Table S2.** Primer sequences used for qRT-PCR

| Gene | Forward primer | Reverse primer |
| --- | --- | --- |
| mouse TNF-α | GCTCCCTCTCATCAGTTCCA | GCTTGGTGGTTTGCTACGAC |
| mouse IL-1β | GCCAACAAGTGGTATTCTCCA | TGCCGTCTTTCATCACACAG |
| mouse IL-6 | CTTCACAAGTCGGAGGCTTAAT | AGTGCATCATCGTTGTTCATAC |
| mouse iNOS | GGGAGCCACAGCAATATAGG | TCAGCCTCATGGTAAACACG |
| mouse IL-4 | ATCATCGGCATTTTGAACGAGGTC | ACCTTGGAAGCCCTACAGACGA |
| mouse β-actin | GGCTGTATTCCCCTCCATCG | CCAGTTGGTAACAATGCCATGT |
